# Rhinosinusitis: Evidence and experience – 2024

**DOI:** 10.1016/j.bjorl.2025.101595

**Published:** 2025-05-20

**Authors:** Fabrizio Ricci Romano, Wilma Terezinha Anselmo-Lima, Eduardo Macoto Kosugi, Eulalia Sakano, Fabiana Cardoso Pereira Valera, Marcus Lessa, Renato Roithmann, Shirley Pignatari, Alexandre Wady Debes Felippu, Camila Degen Meotti, Carolina Cincurá Barreto, Dirceu Solé, Ekaterini Simões Goudouris, Fábio Chigres Kuschnir, Fabio de Rezende Pinna, Faradiba Sarquis Serpa, Gabriela Ricci Lima Luz Matsumoto, Gustavo Subtil Magalhães Freire, João Ferreira Mello, José Laerte Boechat, Leonardo Lopes Balsalobre Filho, Marcel Menon Miyake, Marcio Nakanishi, Marco Aurélio Fornazieri, Mariana Dalbo Contrera Toro, Miguel Soares Tepedino, Norma de Paula Motta Rubini, Olavo de Godoy Mion, Ricardo Landini Lutaif Dolci, Richard Louis Voegels, Roberto Eustáquio Guimarães, Sérgio Duarte Dortas, Thiago Freire Pinto Bezerra, Vanessa Ramos Pires Dinarte, Edwin Tamashiro, Otávio Bejzman Piltcher

**Affiliations:** a Faculdade de Medicina da Universidade de São Paulo (FMUSP), São Paulo, SP, Brazil; b Faculdade de Medicina de Ribeirão Preto, Universidade de São Paulo (FMRP-USP), Ribeirão Preto, SP, Brazil; c Escola Paulista de Medicina, Universidade Federal de São Paulo (EPM-UNIFESP), São Paulo, SP, Brazil; d Faculdade de Ciências Médicas da Universidade Estadual de Campinas, Universidade Estadual de Campinas (UNICAMP), Campinas, SP, Brazil; e Faculdade de Medicina da Universidade Federal da Bahia (UFB), Salvador, BA, Brazil; f Universidade Luterana do Brasil, Canoas, RS, Brazil; g Instituto Felippu de Otorrinolaringologia, São Paulo, SP, Brazil; h Faculdade de Medicina da Universidade Federal do Rio Grande do Sul (FAMED-UFRGS), Porto Alegre, RS, Brazil; i Universidade Federal do Rio de Janeiro, Rio de Janeiro, RJ, Brazil; j Faculdade de Ciências Médicas, Universidade do Estado do Rio de Janeiro, Rio de Janeiro, RJ, Brazil; k Faculdade de Medicina da Universidade de São Paulo (USP), São Paulo, SP, Brazil; l Escola de Ciências da Santa Casa de Misericórdia de Vitória, Vitória, ES, Brazil; m Hospital Universitário de Brasília, Universidade de Brasília (HUB-UnB), Brasília, DF, Brazil; n Faculdade de Medicina, Universidade Federal Fluminense (UFF), Niterói, RJ, Brazil; o Faculdade de Ciências Médicas da Santa Casa de São Paulo, São Paulo, SP, Brazil; p Faculdade de Medicina da Universidade de Brasília (FM/UnB), Brasília, DF, Brazil; q Universidade Estadual de Londrina (UEL), Londrina, PR, Brazil; r Faculdade de Medicina da Universidade Federal de Minas Gerais (UFMG), Belo Horizonte, MG, Brazil; s Faculdade de Medicina de Petrópolis (FMP/UNIFASE), Petrópolis, RJ, Brazil; t Universidade Federal de Pernambuco (UFPE), Recife, PE, Brazil; u Hospital das Clínicas da Faculdade de Medicina de Marília (HCFamema), Marilia, SP, Brazil

**Keywords:** Rhinosinusitis consensus

## Abstract

It has been 10-years since the publication of Rhinosinusitis: evidence and experience, and since then a lot has changed in our understanding of the disease. Advances in pathophysiology, endotyping and new treatments such as biologics brought a new era in the management of our patients. This new guideline, developed jointly by ABR and ABORL-CCF, with the help of ASBAI presents an updated, evidence-based approach to the different forms of rhinosinusitis that aims to improve the diagnosis and treatment of this complex disease. The document covers a wide range of topics, including clear definitions of the different stages of acute sinusitis. It also introduces a new term called Prolonged Acute Viral Rhinosinusitis. Reviews phenotypes and endotypes of chronic rhinosinusitis, recommending methods for clinical and laboratory investigation, clinical and surgical treatment. We also discuss in detail fungal sinusitis and pediatric sinusitis. The objective of this updated Consensus is to clarify some already established and recent concepts, highlighting the importance of an accurate diagnosis to promote treatment approaches that reflect the best practices based on solid evidence. Therefore, we seek not only to improve the results of patients care, but also to guide thealth professionals through a clinical panorama that is in constant transformation.

## Highlights

• Prolonged Acute Viral Rhinosinusitis is a cold lasting more than 7–10 days, with slow and progressive improvement, reinforcing its typically viral origin.
• Antibiotics should be used only in severe, unequivocally diagnosed ABRS. For moderated cases, the “watchful waiting/delayed prescription”, is recommended.
• Suspicion of orbital complication should be investigated with contrast CT and hospitalization under intravenous antibiotic therapy. Consider surgery.
• Surgery plays a very important role in the management of CRS. Either curative or providing control of the disease combined with medical treatment.
• Biologics are a good treatment option for some patients with severe CRSwNP refractory to standard treatment.

## Introduction

In the dynamic field of Otorhinolaryngology, a comprehensive understanding of rhinosinusitis continues evolving, reflecting the confluence of constant new research, advancing technologies and well-established clinical practices. Recognizing the need to keep the medical community updated with the latest evidence and practices, a group of experts came together to review and update the latest Brazilian “Consensus on Rhinosinusitis 2017”. This meeting of Brazilian leaders in research and clinical practice, culminated in the publication of: “Rhinosinusitis: Evidence and Experiences ‒ 2024”. This document is an important tool for all professionals involved in the diagnosis and treatment of rhinosinusitis, providing a solid basis for grounded and effective clinical practice. The main objective of this updated Consensus is to clarify some already stablished and recent concepts, highlight the importance of an accurate diagnosis and promote treatment approaches that reflect the best practices based on solid evidence. Therefore, we seek not only to improve the results of patients care, but also guide health professionals through a clinical panorama that is in constant transformation.

### Definition of rhinosinusitis

Rhinosinusitis is defined as an inflammatory process of the mucosa of the nose and paranasal sinuses. This inflammation may be due to infectious and/or non-infectious causes. Rhinosinusitis can be classified according to its frequency and the duration of the inflammatory process.^1^, ^2^

#### Acute Rhinosinusitis (ARS)

It is characterized by symptoms which last up to 12-weeks. The diagnosis is based on the presence of 2 or more symptoms: Nasal obstruction; Anterior or posterior rhinorrhea; Facial pain or pressure; Reduction or loss of smell (or cough in pediatric patients).

There is no need for radiological or endoscopic for diagnosis. In pediatric patients, the alteration of smell criterion is replaced by coughing.^3^, ^4^

#### Chronic Rhinosinusitis (CRS)

It is defined by symptoms that last more than 12-weeks. The patient must present at least two of the following symptoms: Nasal obstruction; Anterior or posterior rhinorrhea; Facial pain or pressure; Reduction or loss of smell (or cough in pediatric patients).

In addition, there must be evidence of inflammation in the nasal endoscopy and/or computed tomography.^3^, ^4^ In this document, we group CRS according to the Presence (CRSwNP) or absence of Nasal Polyps (CRSsNP).

#### Recurrent Acute Rhinosinusitis (RARS)

4 or more episodes of ABRS, with periods of normality between crises. Note that we are only considering bacterial ARS (ABRS), therefore being necessary to differentiate from episodes of common colds, for which therapy is usually based only on therapy is usually based on symptomatic medications, Evidence of normality between crises is necessary to differentiate between RARS and CRS.

It is worth noting that these symptoms time-based classification is arbitrary and does not consider the underlying pathophysiology. In general, acute rhinosinusitis is predominantly of infectious origin, while CRS represents a more complex and multifactorial process. Just as ARS can be caused by different agents, CRS is a generic term that covers several diseases with distinct pathophysiology and mechanisms, which should be correctly identified to obtain the best treatment.

## Acute rhinosinusitis

The diagnosis of ARS should be focused on differentiating between viral and bacterial etiology, which guides the choice of treatment. Following this therapeutic reasoning, the terms common cold, and Viral ARS (AVRS) will be considered synonymous in this document. It is estimated that adults may present two to five episodes of AVRS, and school-age children, seven to ten common colds per year.^5^ The high incidence of ARS, associated with frequent seeking of medical care in these episodes, makes ARS one of the most common diseases diagnosed in an outpatient setting, accounting for 2%–10% of otorhinolaryngological and primary care consultations.^6^ Although the majority of cases are self-limited, these infections have a significative impact on patients’ lives, many ending up taking a large number of medications often unnecessary and associated with several adverse effects. The distinction between viral and bacterial conditions and cost-benefit assessment of the varied therapeutic options becomes essential in terms of collective and individual health. The importance of this theme should be discussed with patients.

### Differentiation between viral and bacterial acute rhinosinusitis

#### Natural history of acute viral rhinosinusitis

Acute rhinosinusitis is a self-limited disease, being essentially viral in the first 7- to 10-days, with a clear improvement after the fifth daylf there is no significant worsening of symptoms or signs of complications, symptoms that last for more than 10-days are still predominantly viral. In some situations, viral symptoms can last more than 3-weeks. It is estimated that only 0.5%–2% of AVRS evolve into bacterial infection in adults, and 5 %–13 % in children.^7^, ^8^, ^9^ Viral diseases have a well-defined temporal evolution, with abrupt worsening in the first and subsequent days, followed by gradual improvement that can vary from a few days to about 3–4-weeks. Rhinorrhea, sneezing, sore throat and headache start at the beginning of the process. Nasal obstruction, malaise, myalgia and chills usually appear in the next days. At the end, cough appears. This temporal sequence illustrates three stages of the disease (AVRS): first, symptoms of the surface on primary sites (nose) and its drainage (throat); second, deeper inflammation into the tissues and body’s response to infection (viremia); and third, the affected tissues begin to reestablish.^9^, ^10^

In children, observe a biphasic distribution of symptoms: in the first three days, fever, sleep disturbance, irritability, adynamic behavior and headache prevails; from the 4th day, runny nose, cough, sore throat and lack of appetite dominate the symptoms, which may persist for up to 2-weeks.^11^ Sometimes recovery from AVRS may be slow taking up to 3–4-weeks for a complete resolution.

The Brazilian Academy of Rhinology (ABR) recommends that conditions of Acute Viral Rhinosinusitis (AVRS) lasting more than 7- to 10-days, with a slow and progressive improvement, be classified as Prolonged Acute Viral Rhinosinusitis (PAVRS). This terminology reinforces the concept that even prolonged episodes are typically of viral origin.

In conclusion, the natural history of acute viral rhinosinusitis exhibits a clear pattern of symptom evolution, characterized by an intense peak in the first few days, followed by gradual improvement that usually spans several more days but can last up to 3 to 4-weeks. The high intensity of symptoms in the initial days is not indicative of a bacterial infection and should not be used as a criterion for prescribing antibiotics. Additionally, the duration of the condition alone should not be considered, as viral infections can be prolonged while typically demonstrating gradual improvement.

#### Acute Bacterial Rhinosinusitis (ABRS)

When the patient presents clinical worsening after the fifth day, or lack of improvement after the tenth day, one should consider the possibility of progression to secondary bacterial infection.^3^ No isolated symptom is pathognomonic of ABRS. It is estimated that purulent secretion has sensitivity of 77% and specificity of 54%; relapse of symptoms, 74% and 41%; congestion and obstruction nasal, 83% and 24%, respectively.^12^ A reflex of this low specificity is seen in the study of Rubin *et al*, where even among patients who presented the 3 main symptoms (rhinorrhea, nasal obstruction and facial pain) for more than 10 days, only 40%–50% had proven ABRS.^13^ Therefore, to increase the specificity of the ABRS diagnosis, EPOS2020 uses a pattern of unilaterality and severity, considering that, in addition to symptoms that last for more than 10-days, the patient must present at least 3 of the following 5 symptoms: yellowish discharge with unilateral predominance, intense local pain (usually unilateral), fever >38 °C, C-Reactive Protein (CRP) and/or increased Erythrocyte Sedimentation Rate (ESR), and double worsening (improvement followed by worsening).^4^ The ICAR-RS-2021 uses this same criterion of unilaterality and severity for diagnosing ARSB in children. In adults, the ICARRS-2021 only considers the duration and evolution of the symptoms for the diagnosis of ABRS, focusing mainly in the “double worsening” criterion. However, once the diagnosis of ARSB is made, the recommendation of the American guideline is to wait 1 more week before starting with antibiotics.^3^

It is worth remembering that many patients present comorbidities, such as allergic rhinitis, which can be exacerbated by viral infections, which could interfere with the complete resolution of the nasal symptoms, not necessarily being indicative of an evolution towards ARSB. Since the clinical picture of upper airway infections are usually very similar, etiological differentiation can be difficult before 10-days of symptoms.

According to the Brazilian Academy of Rhinology (ABR), the diagnosis of Acute Bacterial Rhinosinusitis (ABRS) should be suspected when the patient exhibits symptoms of ARS with one of the following criteria:

• **Significant worsening of symptoms: (increased intensity/severity and/or signs of unilateral involvement) after initial improvement during the first 5 days ‒ referred to as “double worsening”; or**
• **Persistence of initial intense symptoms without gradual improvement after 14-days.**

Increased intensity or severity is indicated when the patient experiences a worsening of symptoms ‒ particularly pain and rhinorrhea ‒ or the onset of fever after the normal peak of symptom worsening, which occurs in the first 2- to 3-days. This last described situation represents a relapse following an initial improvement in the patient’s condition. Signs of unilaterality include a change in symptom patterns, characterized by clear unilateral predominance, especially in pain and rhinorrhea, after a more diffuse (bilateral) onset that is typical of viral infections.

**Fig. 1:**
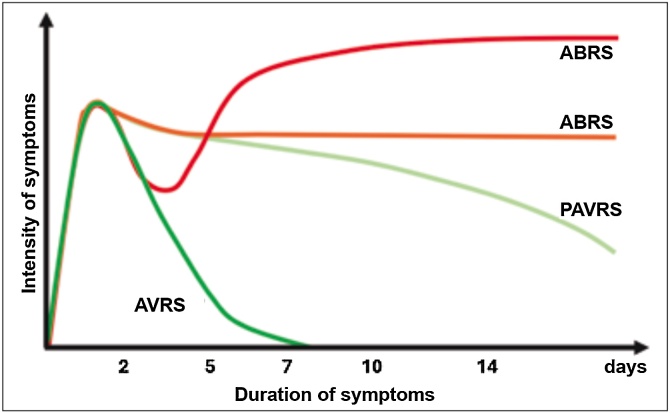
Duration and Evolution of the symptoms of Acute Rhinosinusitis. AVRS, Acute Viral Rhinosinusitis; PAVRS, Prolonged Viral Acute Rhinosinusitis; ABRS, Acute Bacterial Rhinosinusitis.

The Brazilian Academy of Rhinology (ABR) recommends that conditions of Acute Viral Rhinosinusitis (AVRS) lasting more than 7–10 days, with a slow and progressive improvement, to be classified as Prolonged Acute Viral Rhinosinusitis (PAVRS). This terminology reinforces the concept that even prolonged episodes are typically of viral origin (Fig. 1 ).

### Diagnostic criteria for acute rhinosinusitis

The diagnosis of ARS is essentially clinical, and can be made without radiological images or endoscopy, according to the Table 1. Besides that, isolated or combined clinical parameters are all of limited diagnostic value^12^ and do not predict response to antibiotic treatment.^14^

**Table 1:** Diagnosis of acute rhinosinusitis.

Disease	Criteria
Acute Rhinosinusitis (ARS)	Two or more symptoms for up to 12-weeks:
	Nasal obstruction/congestion
	Anterior/posterior rhinorrhea
	Facial pain/pressure
	Olfactory reduction (cough in children).
	No need for physical and radiological examination for diagnosis
Acute Viral Rhinosinusitis (AVRS)	Criteria for ARS
	Symptoms are more intense in the first 2–3 days.
	Gradual improvement, usually within 14-days.
Prolonged Acute Viral Rhinosinusitis (PAVRS)	Criteria for ARS;
	Symptoms are more intense in the first 2–3 days.
	Slower gradual improvement, which may last 3–4 weeks or more
Acute Bacterial Rhinosinusitis (ABRS)	Criteria for ARS,
	AND
	Significant worsening of the condition (increased intensity/severity and/or signs of unilaterality) after improvement in the first 5-days ‒ double worsening
	Or
	Maintenance of the initial intense condition without gradual improvement after 14-days.

Atypical or very different conditions from those described above should be referred to a specialist for further elucidation.

#### Symptoms

Purulent rhinorrhea, despite being classically associated with bacterial etiology, has not demonstrated to be an adequate predictor for ABRS, neither in adults or in children, as the prevalence of bacterial growth (puncture of the maxillary sinus or middle meatus cultures guided by endoscopy) ranged from 31% to 61.1%.^12^, ^15^, ^16^

Facial and dental pain on the other hand, especially when unilateral, showed to be better predictors symptoms.^12^ Anterior rhinoscopy, although providing only a limited view of the anterior region of the nasal cavities, is capable to reveal signs of inflammation, such as edema, hyperemia and rhinorrhea.^17^

The differential diagnosis between viral and bacterial sinus infection and their treatment should not be based on isolated symptoms, due to their low positive predictive value, but rather in the binomial “duration and evolution” of symptoms. It is always worth highlighting that the typical viral symptoms worsen abruptly in the first few days with gradual improvement after the fifth day. Completeness of improvement may take up to 3–4-weeks. Therefore, a lasting longer than 10-days symptomatology alone does not necessarily mean conversion to bacterial infection.

#### Complementary exams

##### Nasal endoscopy

Nasal endoscopy, preferably following vasoconstriction, allows adequate inspection of the middle meatus, sphenoethmoidal recess and rhinopharynx. When a more precise definition of the etiological agent is necessary, especially after previous empiric antibiotic therapy failure, nasal endoscopy can be useful. In addition to more precise inspection, it allows the collection of secretion for cultures in the middle meatus of patients with suspected ABRS.

##### Viral tests

Most cases of viral infections of the upper airways ends up without a specific etiological diagnosis. In recent decades, pandemics caused by Influenza viruses and more recently by SARS-CoV-2 (COVID-19) accelerated the development and access to rapid testing antigens and molecular tests (RT-PCR – Real Time Polymerase Chain Reaction). Molecular tests are more sensitive and specific, being considered the reference standard test for diagnosis of influenza viruses or COVID-19, however, the results may take hours or days to be delivered depending on the demand of the laboratory, which makes the rapid antigen tests more attractive since they run faster and not esistancly depends on laboratories. As a basic rule, positive results of rapid antigen test are enough for the diagnosis, however negative results when there is high clinical suspicion, requires a molecular testing.

Specifically for influenza and SARS-CoV-2, early detection allows the introduction of specific treatment, or even favors prevention of the spread of these viruses isolating positive cases. In addition to these 2 specific virus tests there are molecular tests called “respiratory virus panels” for multiple respiratory virus” that run concomitantly, with a single swab sample, such as for Influenza A and B and Coronavirus (including SARS-CoV-2), besides Rhinovirus, Adenovirus, Respiratory Syncytial Virus, Metapneumovirus, Parainfluenza and several others. Depending on the evolution and severity of the symptoms, these exams can be useful for decision making, and for epidemiological purposes. However, considering the lack of specific treatment and the self-limited nature of these diseases for most of viruses but Influenza virus or SARSCoV-2, there is no formal indication to routinely carry out this exam.

##### Culture

Nasopharyngeal or sinus cultures are not essential for the diagnosis of ABRS but can assist in choosing the antibiotic treatment in specific cases.^18^ One of the limitations of nasopharyngeal secretion collection is the possibility of contamination of the sample by colonizing bacteria, such as *Streptococcus pneumoniae* or *Haemophilus influenzae* and *Moraxella catarrhalis*; thus, positive culture does not always indicate infection. Yet, a recent study demonstrated that the identification of *Streptococcus pneumoniae* and *Haemophilus influenzae* in the rhinopharynx in patients with clinical diagnosis of ARS was associated with more positive impact of antibiotic treatment among children.^16^

Endoscopic guided middle meatus culture is a highly sensitive and accurate diagnostic method for ARS and may be more sensitive than antral lavages culture, given the fact that pathogenic bacteria are not usually identified in antral lavages. Endoscopy-guided culture is a viable and preferred method for determining effectiveness of antimicrobial agents and bacterial resistance profile.^19^

##### C-reactive protein and ESR

C-Reactive Protein (CRP) and Rate of Erythrocyte Sedimentation Rate (ESR) are inflammatory biomarkers that are increased in bacterial infections diseases. In doubtful cases, these tests can be performed in serum samples and used as a predictor of bacterial diseases. Nonetheless, their limited specificity indicate that routine use is not justified.^20^

##### Imaging exams

Imaging exams are not part of the arsenal diagnosis of uncomplicated ARS. These exams involve additional costs and exposure to radiation.

Common imaging findings in individuals with ARS include thickening of the sinus mucosa, air-fluid levels and blisters and, obstruction of the ostiomeatal complex.^21^ However, most patients with common colds also presents with sinuses opacification due to the presence of mucus into the paranasal sinuses, which makes these imaging observations inadequate to differentiate between viral and bacterial etiology.^22^ Furthermore, these findings can be seen in both, patients with ARS and in bedridden patients, in cases of trauma and even in asymptomatic patients.^23^ Computed tomography and/or magnetic resonance imaging are reserved for cases of RS complications and/or suspicion of other diagnoses (to rule out acute rhinosinusitis).

The ABR recommends that in typical and non-severe cases, the duration and evolution of customary symptoms be used to diagnose acute rhinosinusitis, without the need of complementary exams. Complementary measures can be considered in atypical cases or when there is doubt in the diagnosis, especially after treatment failure.

### Treatment of acute rhinosinusitis

The objective of treating a common cold and an acute viral rhinosinusitis is to provide relief of symptoms, reduce convalescence time and to avoid complications. This is a cost-effectiveness perspective. The benefits of an intervention must be higher than the costs and higher than the short-term and long-term risks or the just “waiting and see” strategy. Avoiding adverse effects from different treatments may be more relevant than generating an insignificant clinical impact.

A very large number of patients are need to be treated (NNT ‒ Number Needed to Treat) to prevent one additional bad outcome suggests low-efficacy of the treatment for this group of patients.

Prevention must be obtained by reinforcing hygiene measures, such as regular handwashing, wearing masks and social distancing when recommended.^24^, ^25^, ^26^, ^27^ Regarding immunity, breastfeeding plays a very important role, while probiotics are still navigating in the field of hypothetical and indirect evidence about how they would act on the human microbiome, or increasing defenses against viruses. This modality of therapy, as well as herbal medicines, minerals and vitamins, have been occupying a significant space among therapeutic options, all with the same purpose, but still lacking definitive scientific evidence to justify routine use in viral or bacterial infections of the upper airways.^28^, ^29^, ^30^, ^31^

For didactic purposes, this session will cover distinct therapeutic modalities and will classify their role and scientific evidence in viral (colds and AVRS) and bacterial etiology (ABARS). The studies that evaluated the effectiveness of different interventions in patients with post-viral ARS (according to the classification proposed by EPOS2020), will in this document, for practical purposes, be considered of viral etiology and, therefore, included in the first group. However, because many of the included patients had symptoms lasting more than 10-days (bacterial, according to the American patterns) the interpretation of the outcomes, may generate doubts whether the intervention targeted an ongoing viral inflammation or an already stablished bacterial infection. The idea is to reinforce that even potentially bacterial sinus infection can improve without antibiotics.

#### Antivirals

Regarding Influenza viruses, if suspected and diagnosed early (within the first 48-h), treatment with oseltamivir is indicated. This drug decreases viral replication, shortening the duration of the disease and reducing the risk of complications such as ABRS.^32^

The ABR recommends the use of oseltamivir in severe cases of Influenza with early diagnosis (within 48-h onset of symptoms) to prevent potential respiratory complications.

For SARS-CoV-2, new antiviral drugs have been developed, such as Paxlovid®, composed by antivirals nirmatrelvir and ritonavir. Currently, it is recommended for adults with confirmed, mild to moderate COVID-19, with high risk of progression to severe COVID-19, including hospitalization or death. It is not recommended for already hospitalized patients due to severe or critical COVID-19, nor for pre- or post-exposure prophylaxis.

There is a clear benefit of the drug in reducing the number and duration of hospitalizations, ICU admissions and emergency room visits, in addition to accelerating the time interval to negative RT-PCR for SARS-CoV-2.^32^, ^33^

Immunization for both diseases is recommended, since the reduction of cases and their severity ends up playing an important role in the prevention of acute viral and bacterial rhinosinusitis.

#### Antibiotics

##### VARS

There is no benefit of using antibiotics for clinical conditions resulting from viral infections even in the presence of purulent rhinorrhea, neither to control symptoms, or to avoid sinus complications.^34^, ^35^

It is always useful to reinforce that this type of medication is associated with more than 20% of adverse events such as allergic reactions, gastrointestinal changes, among others.^36^ Furthermore, attention should be paid to the alarming rates of bacterial resistance, which will be further discussed. Bacterial esistance is already considered a global challenge in terms of mortality. It is estimated that in the coming decades this will motivate deaths of millions of individuals around the world. Lastly, although hypothetical, changes in the gut microbiota may induce disorders of the immune system, triggering autoimmune, atopic and even behavioral disturbances.^37^, ^38^, ^39^

The ABR recommends AGAINST the use of antibiotics for AVRS or PAVRS.

##### ABRS

The latest publication of “Evidence and Experiences” (2015) and the ABORL-CCF position paper on the use of antibiotics (2018) brought a clear message that only an adequate selection of patients with ABRS would justify the prescription of antibiotics in order to shorten the duration of symptoms and for faster resolution of the disease. This position remains current. It is only by acting in this way that it would be possible to minimize the inevitable adverse effects of this class of medication in a disease with high percentage of spontaneous resolution, even when it is bacterial.^1^, ^7^

In recent years, the literature about this subject has not been greatly enriched in quantitative terms. However, so many published clinical trials, such as systematic reviews, including EPOS2020 and ICAR-RS-2021, reinforce the same position.^3^, ^4^, ^40^, ^41^, ^42^ In a recent study of pediatric population the authors highlight the importance of an objective criteria, such as microbiological identification (from nasopharynx), which could identify those individuals to which antibiotic treatment will have the expected impact.^16^ Otherwise, many patients diagnosed with ABRS will continue receiving antibiotics while only a few would actually benefit in terms of symptoms control and faster healing. Overall, for every 100 patients, only 5 will really benefit from the use of antibiotics based on the current clinical criteria. This number increases to 11 and 28 with more specific criteria.^16^, ^42^ In other words, there is scope and need to change this reality.

The major guidelines suggest that for mild and moderate cases of ABRS antibiotics should not be prescribed. When antibiotic prescription is restricted to patients with intense symptoms after 7–10-days (fever >38.3 °C and intense pain in the face and/or unilateral predominance) and/or diagnosed/suspected of complications, the benefits outweigh the adverse effects in addition to individualized assessments for immunocompromised patients.^7^, ^16^, ^42^

An alternative for mild to moderate ABRS cases is the “careful waiting and see/deferred prescription” strategy, which is a remote monitoring of the patient’s evolution. If the patient does not show improvement of this non-serious condition, or rather, if there is a significant worsening of the symptoms after 7-days of consultation, start treating with the prescribed antibiotic.^4^

There is no appropriate evidence to define the duration of antibiotic treatment for ABRS. The vast majority range empirically between 7–10 days. It is very important to note when comparing results of shorter treatments (5‒7-days), that it should be analyzed in a context of nonsevere or uncomplicated ABRS (inclusion criteria of these clinical trials).^43^ Regarding the choice of antibiotics for ABRS, the usual dosage of amoxicillin, alone or associated with clavulanic acid, remain as the first option. In cases at risk of carrying resistant Streptococcus pneumoniae, the dose can be increased to up to 4 g/day without increasing the amount of clavulanic acid.^44^ For patients with a nonsevere penicillin allergy, cephalosporins are recommended, and in cases of more severe allergy, macrolides can be prescribed.

There is a specific concern about quinolones as first-line medications in the treatment of bacterial infections of the airways, such as ABRS, due to the important adverse events associated with these medications. For this reason, there is an official recommendation from internationally recognized institutions that such medications should only be prescribed in cases of lack of an alternative in the treatment of these infections.^45^

In the Table 2, there is a list of antibiotics recommended by the Brazilian Academy of Rhinology (ABR) for the treatment of uncomplicated ABRS.^7^

**Table 2:** Recommended antibiotics for non-complicated ABRS according to the Brazilian Academy of Rhinology.

First choice antibiotics	Posology	Duration	Special considerations
Amoxicillin	500 mg 8/8 h	7 to 10 days	Preferred in patients without history of resistant bacteria, with no prior antibiotic use in the last 30-days.
	875 mg 12/12 h	7 to 10 days	
Amoxicillin/clavulanic acid	500/125 mg 8/8 h	7 to 10 days	Indicated for beta-lactamase-producing bacteria.
	875/125 mg 12/12 h	7 to 10 days	Diarrhea in 1%‒10%.

Options for penicillin allergy	Posology	Duration	Special considerations
Axetilcefuroxime	25 0 to 5 00 mg 12/12 h	7 to 10 days	Similar spectrum to amoxicillin/clavulanic acid, but poor for anaerobes.
Levofloxacin	500 mg 24/24 h	5 to 7 days	Quinolones for ABRS only when there is no other
Moxifloxacin	400 mg 24/24 h	5 to 7 days	Option, due to potential adverse events.
Clarithromycin	500 mg 12/12 h or 24/24 h slow-release presentation	7 to 10 days	Consider in suspected atypical germs or in patients allergic to beta-lactams.
			Contraindicated in concomitant use of statins.
Doxycycline	100 mg 12/12 h	7 to 10 days	Photosensitivity reaction

Options in case of failure	Posology	Duration	Special considerations
High dose amoxicillin	1000 mg 8/8 h	7 to 10 days	Exceptional conduct without evidence of clinical efficacy.
High dose amoxicillin with clavulanic acid	2000/125 mg 12/12 h	7 to 10 days	Observe increased gastrointestinal effects. Consider possible *S. pneumoniae* resistant.
Levofloxacin	750 mg 24/24 hs	5 to 7 days	Quinolones for ABRS only when there is no other
Moxifloxacin	400 mg 24/24 hs	5 to 7 days	Option, due to potential adverse events.
Clindamycin	300 mg 8/8, 300 mg 6/6 h, or 600 mg 8/8 h	7 to 10 days	Option if anaerobes or *S. aureus* are suspected. Take it with 300 mL of water due to the risk of esophageal injury. Caution due to the risk of pseudomembranous colitis and diarrhea due to *Clostridium difficile*.
Cefdinir	600 mg 24/24 h	10 days	Third-generation oral cephalosporin with action on *S. pneumoniae, H. influenzae* and *M. catarrhalis*. It does not have the same spectrum as ceftriaxone, option to axetilcefuroxime.
Ceftriaxone	1 a 2 g 24/24 h	7 days	Intramuscular.

It is worth mentioning that there is a global tendency to reduce the duration of antibiotic therapy, with maintenance of effectiveness. This strategy aims to minimize side effects and the generation of bacterial resistance. We consider “failure” of antibiotic therapy when there is no relevant clinical improvement after 48–72 h of treatment or when there is worsening at any time.

The ABR recommends that antibiotics should be used in unequivocally diagnosed ABRS and, only in the most severe presentations. For moderated cases, the “careful waiting and see/delayed prescription”, is indicated. If the decision is for antibiotic amoxicillin with or without clavulanic acid for 7- to 10-days is the first choice.

### Systemic corticosteroids

Although there is no precise information about the percentage rate of pediatric and adult patients who take corticosteroid for the treatment of colds and/or ARS, the overall sense is that prescriptions or even self-medication are very frequent. While for antibiotics there is a constant concern regarding the abusive consumption, corticosteroids do not receive as much attention, although their use can probably be even greater than antibiotics. There is little published evidence and/or campaigns warning of the risks of excessive use of these medications.

The ABR recommends AGAINST the use of intramuscular depot corticosteroids in all types of ARS.

#### AVRS

There are no studies evaluating the use of oral corticosteroids for the treatment of common colds.^3^, ^4^

The ABR does not recommend the routine use of oral corticosteroids for AVRS.

#### ABRS

In bacterial conditions with intense symptoms (especially pain), the use of oral corticosteroids associated with antibiotics provides greater control of pain and facial pressure, reducing the need of other symptomatic medications. Once again, it is worth noting, as already stated in the last “Evidence and Experiences”, that after two weeks no difference is found between patients who received and those who did not receive oral corticosteroids.^1^ A systematic review demonstrated that seven patients would need to be treated for one to obtain greater relief of symptoms; the same study call attention to the lack of studies specifically designed for this purpose, making it difficult to defend or contraindicate such treatment.^43^, ^46^, ^47^, ^48^ In view of this week evidence, the increasingly judicious use of oral corticosteroids is recommended in cases of ABRS, avoiding the routine use, even associated with antibiotics, as we see currently. Oral corticosteroids should be reserved only for bacterial infections with intense pain with no improvement after 48 h of antibiotics and topical measures.

The ABR recommends oral corticosteroids in cases of ABRS with severe pain that do not show satisfactory improvement after 48‒72-h of antibiotics and common analgesics and topical nasal therapies, for the shortest necessary time to improve the severity of pain.

#### Intranasal topical corticosteroids

##### AVRS

Although the use of intranasal corticosteroids is frequent in patients with typical viral conditions, the effect of this medication is modest, so much so that there is a contradictory recommendation between the EPOS2020 and the ICAR-RS-2021 regarding this class of drug.^3^, ^4^ The EPOS2020 considers that the modest effect (only 7patients show complete improvement with intranasal corticosteroids per 100 treated) would not justify the costs at the beginning of the condition, when facing an AVRS, therefore, it established a recommendation against intranasal corticosteroids in this situation, but considers beneficial and recommended it in PAVRS with intense symptoms.^4^ The ICAR-RS-2021 considers that the improvement (even modest) in the ARS symptoms (especially nasal congestion and rhinorrhea) and the reduction of the duration of disease, in addition to the very low risk of the drug, the benefit of doubled doses of intranasal corticosteroids is worth it, establishing a strong recommendation for its use in all types of ARS.

According to the ABR, intranasal corticosteroids in AVRS is an option that may benefit severe cases of AVRS.

##### ABRS

Intranasal corticosteroid with doubled dosage as monotherapy or in combination with antibiotics can benefit patients with ABRS, reducing symptoms and reducing the time of illness. Even in mild to moderate cases doubled dose intranasal corticosteroid monotherapy was superior to the isolated antibiotic. It is currently recommended by the American and European guidelines.^3^, ^4^

However, it is worth, once again, emphasizing that due to the favorable evolution of many rhinosinusitis cases without any treatment, it is reasonable to reserve it as an option for very symptomatic cases.

Without studies defining the ideal duration of treatment is best to try to use the minimum number of days necessary to relieve symptoms.^35^, ^49^

The ABR recommends the use of intranasal corticosteroids in double dose in ABRS as an option to antibiotics in mild cases, or in association with antibiotics in severe cases of ABRS. Its consumption must be restricted to the necessary time to improve symptoms.

#### Nasal saline solutions

Nasal saline solutions for symptomatic conditions of the upper airways, including colds/AVRS and ABRS, became a common prescription among health professionals. Currently, the use of high volume in these situations has been widespread, but the literature is poor to confirm that this modality of nasal lavage is superior to the others in the treatment of the ARS. In fact, studies that demonstrated effectiveness in treatment and prevention of URTIs with saline solutions often used low- volume devices.^50^, ^51^ Not to mention that high volume devices produce more adverse effects than those of low-volume, especially regarding the entry of liquid into the middle ear.^52^

Both EPOS2020 and ICAR-RS-2021 and more recently the Manual on Nasal Washings from the Brazilian Association of Otorhinolaryngology and Cervical Facial Surgery (ABORL-CCF)/Brazilian Academy of Rhinology (ABR) agreed regarding the lack of statistically significant evidence. Given the self-limiting nature of the disease this should be specially reminded.^3^, ^4^, ^53^ For this reason, they are considered as an option for the prevention and treatment of viral acute respiratory conditions, largely based on the potential beneficial effects on mucociliary function and improved results in quality of life. As much as with other therapeutic alternatives, well designed studies with control groups are needed to establish the real importance of these procedures.^54^, ^55^, ^56^

The ABR considers nasal irrigation optional in the treatment of ARS. It can be beneficial but must be well guided by the doctor regarding the device and tonicity that is most comfortable and adequate to each patient. The risk of penetration of liquid in the middle ear when high volume and high-pressure devices are chosen should be also considered.

#### Oral and intranasal decongestants

These are widely used medications in medical prescription and self-medication to control the rhinorrhea and nasal congestion associated with acute sinonasal inflammatory processes in this context, there are few novelties in terms of medical literature in recent years.

Intranasal decongestants provide a relief of congestion temporarily in children and adults. Considering once more that the ARS is usually a self limiting process, the potential systemic effects of these drugs, besides the risk of addiction in patients with a history of allergic rhinitis and chronic nasal obstruction, these are not medications that should be recommended as routine option. Regarding systemic decongestants, in our country they are commercially available in associations with antihistamines and analgesics, there may be some benefit in terms of short-term symptomatic relief.^35^, ^54^, ^55^, ^57^, ^58^, ^59^

Nasal decongestants are drugs that belong to the group of adrenergic stimulants or adrenomimetics, whose main action is vasoconstriction, producing rapid relief of nasal blockage. Among the systemic options, the most used compounds are pseudoephedrine and phenylephrine. By acting systemically, they can lead to side effects such as insomnia, headache, nervousness, anxiety, tremors, palpitations, urinary retention, increased blood pressure and other adverse effects.

Pseudoephedrine, which belongs to the family of amphetamines, should be used with caution due to its psychotropic action and potential cardiovascular side effects. In general, is not recommended for patients under four years of age, due to the greater risk of toxicity and, extended-release formulation doses of 120 mg are not recommended for children under 12-years of age. The phenylephrine, in turn, is largely inactivated when ingested orally, which potentially reduces its action.

In Brazil, systemic decongestants are only available in combination with H1 antihistamines first or second generation and/or analgesics.^60^

Intranasal decongestants such as oxymetazoline, xylometazoline and phenylephrine are α-adrenergic agonists which act as topical vasoconstrictors reducing the edema of the nasal mucosa. In the short term reduce nasal congestion/blockage, but their prolonged usage (for more than 3- to 5-days) can lead to dependence (drug-induced rhinitis).

The onset of action occurs within 10 min, and the duration of the effect, can last up to 12-h. These medications, as well as systemic decongestants, can cause important cardiovascular and central nervous system effects, being contraindicated in children under six years of age. They should also be avoided in the elderly, due to the higher incidence of side effects such as hypertension and urinary retention in this age group.

The ABR recommends that oral and intranasal decongestants as symptomatic medications should be used very judiciously and only for short periods of time, especially in patients at higher risk of adverse events.

#### Antihistamines

This is another class of medication that is frequently prescribed by health professionals or used as self-medication by patients for acute inflammatory and infectious conditions of the upper airway. Alone, they are not beneficial, but when associated with a decongestant, as mentioned above, may bring greater symptom relief than placebo for a short period of time. It is an option to be judged judiciously for adult use, perhaps with more prominent benefit if the patient also has allergic rhinitis as an underlying disease.^61^

There is no recommendation level for these medications in the ARS treatment.

#### Anti-inflammatory drugs and common analgesics

Both Non-Steroidal Anti-Inflammatory Drugs (NSAIDs) and the common painkillers (paracetamol and dipyrone) have analgesic potential but do not change the natural history of the disease. NSAIDs are drugs that carry potential serious adverse effects and should not be routinely recommended in the treatment of superior airway infections, giving preference to common analgesics.^3^, ^62^, ^63^

The ABR recommends common analgesics to the treatment of ARS.

#### Mucolytics

From a theoretical point of view, it is a medication to help in reducing the viscosity of secretions and thus facilitating the mucociliary function. However, so far, the available evidence is not sufficient for this class of medication be indicated or contraindicated in the treatment of AVRS or ABRS.^64^, ^65^

There is no level of recommendation for mucolytics in the treatment of ARS.

#### Herbal medicines

For more than 20-years, publications have been evaluating the effects of different herbal medicines on viral and bacterial infections of the respiratory tract. There is evidence that some phytomedicines such as Pelargonium sidoides influence reducing symptoms and time of illness. Although the results indicate a beneficial role in control of symptoms and duration of illness, criticisms exist regarding the design of the studies.^3^, ^4^, ^66^

There is no recommendation level for the use of these medications to treat ARS.

#### Probiotics and immunomodulators

Similarly, to the herbal medicines, although some studies have shown a role in preventing viral and bacterial infections, the quality of evidence does not allow us to recommend these medications as part of routine treatment or for preventive purpose of common colds, AVRS and ABRS.^4^, ^67^, ^68^

There is no recommendation level for the use of these classes of medications in the treatment of ARS.

### EVIDENCE and EXPERIENCE proposals for ARS (viral and bacterial)

• Paracetamol with or without decongestant, dipyrone and/or ibuprofen for the beginning of the inflammatory process aiming to relieve pain/general discomfort.
• Nasal saline solution from the onset of symptoms. Tonicity and frequency according to patients’ preferences. Maintenance of nasal irrigation if they feel comfortable and have symptoms improved.
• Intranasal corticosteroid in double dose, which can be used from the beginning of the symptoms (patient aware of the likely small effect), or after 7-days of persistent complaints and/or suspected bacterial infection.
• Persistent symptoms for more than 7-days, associated with worsening of the clinical picture (increasing intensity/severity and/or signs of unilaterality) after improvement in the first 5-days (double worsening), or associated with maintenance of initial intense symptoms without gradual improvement after 14-days; antibiotics may be of benefit. If after 48–72-h no improvement is noted or very intense symptoms persist, consider adding oral corticosteroids for 3–5 days in a single daily dose preferable in the morning (Table 2).

## Recurrent Acute Rhinosinusitis (RARS)

### Definition and investigation

The most accepted definition for Recurrent Acute Rhinosinusitis (RARS) is the occurrence of four or more episodes of acute rhinosinusitis per year, interspersed by asymptomatic periods between episodes. However, there are conceptual differences between the European and American^3^, ^4^:

a) EPOS2020: episodes of RSA must be post-viral or bacterial, while ICARRS-2021: consider any RSA episode (whether viral or bacterial) for the diagnosis of RARS.
b) EPOS2020: proof by endoscopy or CT scan of at least one episode of the RSA episodes is required, while ICAR-RS-202: requires endoscopic or tomographic examination confirming normality between episodes.

Therefore, the European guideline is more restrictive in the diagnosis of RARS, while the American guideline is more permissive, reflecting the differences of the health systems in each location.^3^, ^4^

ABR defines RARS as 4 or more episodes of ABRS per year, with periods of normality between crises. Only bacterial infections are considered for this diagnosis due to the necessary differentiation from frequent cold episodes, which usually presents a more benign daily life repercussion. Confirmation of normality between pisodes is necessary to differentiate RARS from CRS.

The literature on predisposing factors for RARS is still scarce. It is known that most episodes occur following a AVRS and that some patients have immunodeficiencies, mainly IgA deficiency or common variable immunodeficiency.^3^ A study with 94 children with RARS reported IgG deficiency in 78.7% and allergic rhinitis in 35.1% of the cases.^69^ Other possible predisposing factors include cystic fibrosis, ciliary dyskinesia and asthma. Some authors believe that anatomical factors could predispose to RARS, but the data are conflicting in the literature.^70^, ^71^, ^72^, ^73^

Therefore, it seems reasonable to investigate anatomical factors, allergic rhinitis and immunodeficiencies in patients with RARS. Further investigation may be indicated targeting specific aspects of each case (for example: investigation for cystic fibrosis and PCD in cases of concomitant infections of the lower airway). Often, part of this investigation, such as for respiratory allergies, is already performed during the follow-up of the patient, even before fulfilling the criteria of RARS. Regarding tomography, the best time to carry it out is between bouts.^3^

### Predisposing factors

#### Anatomical factors

Anatomy can play an important role in some patients with RARS, although some alterations can also be found in the general population. One Dutch study showed that 43% of the normal population presents changes on paranasal sinus tomography, and in 14% a significant change, evidenced by a Lund-Mackay score ≥4.

Other studies found lower percentages, but still significant, 17% and 7.4%, respectively.^74^, ^75^, ^76^

It is possible that anatomical variations are less diagnosed in children due to the low recommendation of imaging exams and endoscopies in this age group.^77^

A small sampling study compared anatomical differences between patients with RARS versus control group, suggesting that anatomical changes in the ostiomeatal complex could predispose to RARS, with important surgical implications. The authors observed a significant greater number of infraorbital cells (“Haller cells”) and a smaller diameter of the infundibulum in patients with RARS, compared to the group control. Only a statistical tendency was found towards an association between deviated septum and concha bullosa in patients with RARS, but the small sample size did not allow a definitive conclusion.^70^

A more recent study, also with small sample size, observed association between type 2 frontal cells and longer lasting (in years) of RARS. Association between other variants and RARS was not confirmed.^70^, ^72^

A more recent study with a larger sample (*n* = 160) evaluated the anatomical variations that can affect the ostiomeatal complex, however, some methodological issues made difficult any extrapolation of results.^78^

ABR considers that if a surgery for RARS is being contemplated, anatomical findings that potentially lead to obstruction of the drainage routes must also be surgically addressed.

#### Allergy

Few studies have evaluated the clinical factors and possible comorbidities associated with RARS. This fact can be due to the difficulty in differentiating RARS from CRS or acute exacerbations of CRS, as pointed out by international guidelines.^3^, ^4^ Allergic rhinitis, asthma, primary antibody immunodeficiency and autoimmune disorders are the most related comorbidities.^74^, ^79^ In fact, few studies have evaluated the relationship between RARS and allergy. Choi *et al* investigated the predisposing factors associated with CRS, acute and subacute RS in children under 13-years and observed that the prevalence of allergic rhinitis, atopy and asthma are significantly higher among patients with CRS compared to other patients. On the other hand, Veskitkul *et al* evaluated the clinical characteristics and predisposing factors of RARS in children and found that allergic rhinitis is present in 35.1% of the patients. The absence of a control group limits the interpretation of these results.^69^, ^79^

A systematic review that studied the association between allergy versus ARS and CRS in children, failed to establish a relationship between allergy and acute rhinosinusitis because of the limited data. Despite the limitations, the authors documented a significant reduction in frequency and intensity of upper respiratory tract infections, including the common cold, in patients undergoing anti-allergy treatment.^80^

Behnke *et al*, retrospectively evaluated adult patients with RARS (*n* = 81), comparing them to patients presenting headache and/or facial pain syndrome (*n* = 65). Patients with RARS were older and had a higher frequency of comorbidities: allergic rhinitis (53.1% × 9.2%, respectively), asthma (33.3% × 15.4%, respectively), primary antibodies immunodeficiency (13.6% × 1.4%, respectively), and autoimmune diseases (20.3% × 7.7%, respectively). The tomographic (Lund-Mackay score) and endoscopic (Lund-Kennedy score) assessments showed slight changes. After a multivariate analysis, asthma showed no significant difference.^74^

Acute allergic rhinitis is usually associated with edema of the nasal mucosa, causes disturbance of important mechanical and immunological defense functions; it increases production of fluid mucus, decrease ciliary beats, promotes edema/obstruction of the ostium of the paranasal sinuses, retention of secretion, increasing of expression of innate immunity receptors that is important for virus adhesion, among others.^81^

Furthermore, even after the exposure to the triggering allergen, the inflammation persists, called minimal persistent inflammation that justifies the large lability that some patients have towards many irritants.^81^, ^82^ The knowledge about changes in the mucociliary lining in atopic patients corroborates with the concept of a relationship between allergic rhinitis and ARS. However, the available evidence for both, a possible role in ARS physiopathology, and the importance of allergy treatment for prevention and management of RSA, does not allow now, to confirm this relationship.^80^

The ABR considers that allergic rhinitis should be remembered and appropriately treated in patients with RARS. Although there is a logic indicating its importance in the pathophysiology of ARS and RARS, so far there is no evidence to confirm this association.

#### Immunodeficiencies

Primary Immunodeficiencies (PID) or Inborn Errors of Immunity (IEI), are a heterogeneous group of genetic diseases whose primary defect is in the immune system, affecting the number and/or function of its different components. The group is made up of almost 500 diseases of quite heterogeneous presentation.^83^, ^84^ However, the most common manifestations are severe and/or recurrent infections, due to common and/or opportunistic germs.^85^ Immunodeficiencies secondary to immunosuppressants medications, or diseases that cause protein loss or related to diseases such as diabetes mellitus, also present with repeated and/or severe infections.^86^

Although primary and secondary immunodeficiencies are recognized risk factors for RARS in any age group this diagnosis is not usually suspected.^87^ Defects predominantly in the production of antibodies or humoral defects are the most common IEI and, are more frequently associated with sinopulmonary infections due to encapsulated bacteria.^88^

The most prevalent humoral defects are selective IgA deficiency (defined as IgA <7 mg/dL, normal levels of IgM and IgG in individuals over four years of age age), common variable immunodeficiency (upper and lower respiratory tract infections, bronchiectasis, autoimmunity, granulomatous diseases and lymphoproliferation, low serum IgG and IgA and/or IgM values, inadequate vaccine response, absence of significant cellular defect, in patients over four years of age) and defect of antibodies against polysaccharide antigens (normal immunoglobulin levels and normal response to antigens proteins).^89^, ^90^

Other defects predominantly from the production of antibodies are rarer, such as X-linked agammaglobulinemias and autosomal recessive defects; hyper IgM; and selective IgM deficiency.^83^, ^84^ Diseases from dysregulation of the immune system that are associated with hypogammaglobulinemia also present with sinopulmonary infections, in association to autoimmune endocrinopathies, cytopenias and others.^83^, ^84^ Defects of the initial factors of the classical pathway or factors of the alternative pathway of the complement system can also lead to recurrent sinopulmonary infections.^83^, ^84^ ARS due to non-habitual infectious agents such as Staphylococcus aureus, *Pseudomonas* spp., *Aspergillus* spp., other fungi or opportunistic microorganisms should guide towards suspicion of cellular or phagocyte defects (numeric or functional).^91^

The investigation must be directed by the type of infectious agent involved or likely, other associated infectious or noninfectious diseases, age group and family history. Therefore, careful clinical history and physical examination is an essential first step. The complementary exams most frequently requested in the initial investigation include complete blood count, dosage of immunoglobulins A, M, G and E, assessment of response to proteins (IgG for measles, rubella, mumps, tetanus and diphtheria) and assessment of response to polysaccharides (IgG for 23 unconjugated pneumococcal vaccine) before and after vaccination. In specific cases, it may be necessary to measure lymphocyte subpopulations (CD3, CD4, CD8, CD19 and CD16/56), Complement Hemolytic activity (CH50), and to evaluate the intracellular capacity of neutrophils (DHR – Dihydrorhodamine) digestion.^84^, ^92^, ^93^ In many cases, diagnosis is only possible through genetic new generation sequencing technology, in specific panels for IEI, exome or complete genome.^84^, ^92^

The ABR considers that, among patients with RARS, it is important to keep in mind and investigate specific alterations of the immune system. In patients with immunodeficiency a multidisciplinary monitoring is mandatory.

### Treatment of RARS

#### Intranasal corticosteroids

A systematic review of 3 clinical trials summarized the impact of intranasal corticosteroids in relieving symptoms in patients with RARS. However, none of the studies defined RARS according to the definition of 4 or more episodes annually with absence of symptoms between crises. The studies included other associated treatments, and all patients were also treated with antibiotics, with differences in types, doses and duration of therapy. Intranasal corticosteroids were used during periods of acute exacerbation and, therefore, the efficacy as a preventive measure is unknown. The best evidence was derived from one low risk of bias trial, providing moderate robustness of evidence that intranasal corticosteroids can accelerate symptoms relief in patients with RARS.^94^

Despite the low quality of evidence, ABR recommends the use of intranasal corticosteroids in patients with a confirmed diagnosis of RARS.

#### Antibiotics

A systematic review evaluated the effectiveness of short-term antibiotics on severity and duration of symptoms and, recurrences in patients with RSAR but failed to identify placebo-controlled studies.^95^ In one study, it was observed that the prophylactic treatment of RSAR in children with azithromycin three times weekly over 12 months significantly reduced episodes from ARS from 5 to 0.5 per year. However, in that same study a high percentage of patients had low levels of IgG subclass (83%). The lack of knowledge about the impact on bacterial resistance and its consequences makes prophylactic use of antibiotics in RARS restricted to very specific situations.^96^

The ABR does not recommend the prophylactic use of antibiotics in RARS, except in specific cases of immunodeficiency.

#### Surgery

According to international guidelines, Functional Endoscopic Sinonasal Surgery (FESS) recommendation for patients with RARS is Grade B evidence. Patients improve symptoms, reduce the need of antibiotics, frequency of episodes and absence from work. Costa *et al* in a robust study of 142 patients comparing FESS and medical treatment, observed that the cohort undergoing FEES experienced a greater reduction of SNOT-22 scores at 3-, 6-, and 12-months follow-up.^71^ Patients also experienced improvement of quality of life, decreased use of antihistamines, less workdays absences and fewer acute episodes, although reductions in antibiotic use were not significant.^97^

Other studies showed an average of 61.2% reduction in antibiotics after surgery, similarly to what happen for CRS cases and, significant improvement in SNOT-20 scores at 6-months of follow-up.^98^, ^99^ Although all above studies met the criteria for RARS, some additional inclusion criteria were different. Rudmik *et al* along with a panel of experts established minimum inclusion criteria for later studies: 4 or more annual episodes of ARS, confirmation of at least one episode through endoscopy or computed tomography, joint decision between patient and doctor, failed cycle of intranasal corticosteroids or significant impact on patient productivity due to RARS.^99^, ^100^

As most studies on children only evaluate the role of adenoidectomy in patients with CRS, it suggests that the recommendation of adenoidectomy due to RARS in children is not very frequent, and that maybe new studies could elucidate its role in this group of young patients with RARS.

The ABR considers ESS an option in patients with RSAR with difficult medical control of recurrences and this should always be a joint decision with the patient.

## Bacterial resistance

More than 50,000 deaths per year in Europe and North America are due to resistant bacteria from diseases previously curable with available antibiotics.

For this reason, the WHO has put the world on alert creating several surveillance programs.^101^, ^102^, ^103^, ^104^

Different studies indicate that more than 50% of patients with viral infections are treated with antibiotics.^104^ Therefore, awareness of the role of health professionals is necessary to mitigate this situation. In practice, developing countries and underdeveloped countries have greater lack of information in comparison to developed countries.^105^, ^106^ This fact not only harms education and measures of warnings against the problem, but makes the empirical treatment difficult in cases where antibiotics are necessary. In our country there are no studies evaluating the bacterial colonization of rhinopharynx and its resistance profile. It makes it difficult to discuss alternative therapies and/or changes in the face of the known abusive use of antibiotics.^107^

It is necessary to study and determine if the percentages of resistance in the local population is like developed countries from where most of this information is generated.^105^, ^106^, ^108^ Furthermore, the few available publications about bacteriology usually address severe and systemic infections, not community infections such as ABRS. Even without definitive evidence, it is reasonable to believe in resistant bacteria in patients over 65-years of age, recently hospitalized for more than 5-days, using antibiotics for at least 30-days and, suffering from some type of immunodeficiency, comorbidities (diabetes, heart failure, renal, suppurative complications and/or toxemic conditions).^44^

In Table 3, sensitivity profile of S. pneumoniae and H. influenzae isolated from invasive bacterial infections.^109^

**Table 3:** Susceptibility profile of *S. pneumoniae* and *H. influenzae* isolates from invasive bacterial infections.

Microbiota	Antimicrobian medication	Susceptibility
*Streptococcus pneumoniae* (non-meningitis)	Penicillin	63.8%
	Ceftriaxone	79.4%
	Sulfametoxazol ‒ Trimetoprim	52.4%
	Erythromycin	53.6%
*Streptococcus pneumoniae (meningitis)*	Penicillin	58.7%
	Ceftriaxone	79.1%
*Haemophilus influenzae*	Ampicillin	81.7%
	Ceftriaxone	100%
	Sulfametoxazol ‒ Trimetoprim	69.4%

It should be clear that bacterial resistance is a currently feared reality and with frightening predictions for the future. There is little information about the percentage of ABRS resistant microorganisms. However, based on bacterial resistance of invasive infections on other body systems, the current effectiveness of clinical ABRS treatments may indicate that there is an important participation of the local immune system in resolving infectious conditions and/or the ABRS bacteria are not as resistant as in other infections. Nevertheless, given the number of patients using antibiotics for superior airway conditions, evidence demonstrating the absence of any benefit of antibiotics in viral conditions and small benefit in non-serious and uncomplicated cases is to be respected and understood.

## Complications of Rhinosinusitis

Complications of rhinosinusitis result from extrasinus extension of a primary acute infections or CRS exacerbations, with an incidence ranging from 2 to 5 cases per 1 million inhabitants per year.^110^ The orbit is the most common site of complications of ARS, representing 60%–80% of the cases, followed by intracranial and bone infections.^4^

### Orbital complications

Orbital complications of ARS are more frequent in young male children.^3^, ^4^, ^111^ Immunological, anatomical and vascular factors explain the predominance in the pediatric population. This could be explained as children may not have the immune system fully competent, present more fragile bone barriers (thinner and more porous lamina papyracea) and also greater vascularization associated with the pneumatization process of the paranasal sinuses. These factors contribute to the spreading of the infection through a retrograde flow in a valveless venous system.^111^, ^112^ In orbital complications of ARS, the affected sinuses are usually the ethmoid and maxillary.^4^

The infection is polymicrobial, with emphasis on Streptococcus species and Staphylococcus, in addition to anaerobic bacteria. Species of *Haemophilus* and *Moraxella catarrhalis* present lower association with RSA complications,^113^ with post-vaccination reduction of type b *Haemophilus influenzae*. Antibiotic therapy is responsible for improving the evolution and the outcome of ARS complications; however, the prescription of antibiotics does not prevent the occurrence of suppurative complications.^114^

#### Diagnosis

The clinical history is not always obvious in cases of orbital complication of ARS, mainly because it commonly affects children, a population that not uncommonly presents with misleading symptoms. Therefore, a high level of suspicion is mandatory when dealing with complications of ARS. Furthermore, the assessment of a multidisciplinary approach is fundamental, highlighting the importance of the ophthalmological examination. Some signs and symptoms should alert to a possible orbital complication of ARS. This includes periorbital edema and hyperemia, proptosis, decreased ocular mobility, diplopia and reduced visual acuity. Complementary exams are essential in suspected RSA complication for confirmation of the diagnosis and assessment of the extent of the disease. Computed Tomography (CT) imaging with contrast is the exam of choice and allows differentiation between edema/phlegmon and abscess, in addition to bony assessment. The nuclear Magnetic Resonance Imaging (MRI) has better accuracy in the analysis of soft tissue, but it is more expensive, more time consuming and is generally reserved for cases of diagnostic doubt and/or when there is concern about the intracranial involvement.

#### Classification

To understand the classifications of orbital ARS complications some anatomical concepts must be kept in mind. Orbit is defined as a pyramidal shape bone recess of the anterior part of the skull, covered by periosteum called periorbita. The periorbita delimits the subperiosteal (between the periorbita and the orbit) and orbital (inside the periorbita) spaces. Orbital cone is the space composed by the four extraocular rectus muscles (medial, superior, lateral and inferior), delimiting the extra and intraconal spaces. Both the extraconal and intraconal spaces are part of the orbital space. The anterior limit of the orbit is the orbital septum, fibrous membrane extending from the orbital rim up to the upper and lower eyelids, delimiting the pre- and post-septal spaces. The orbital and subperiosteal spaces are post-septal.^115^ Several classifications systematize the complications related to orbit, involving anatomical structures concepts (such as pre- or post-septal, subperiosteal or orbital) or type of infection (such as cellulitis or abscess) and the particularities of these classifications must be discussed. The definitions of each subgroup of classifications of ARS orbital complications follow below:

##### Chandler classification^116^

Stage I ‒ Preseptal cellulitis, characterized by Inflammatory edema and hyperemia in the eyelid, occasional pain. There are no visual deficits or restriction of ocular mobility.

Stage II ‒ Orbital cellulitis, characterized by diffuse edema of the orbital contents with infiltration of the orbital fat, without abscess. Hyperemia, conjunctival edema and proptosis may be present. Visual acuity and ocular mobility may be affected.

Stage III ‒ Subperiosteal abscess: Purulent collection between the periorbita and orbital wall. Proptosis becomes more accentuated with the inferolateral displacement of the eye, pain and restriction in eye movement.

Stage IV ‒ Orbital abscess: Purulent collection in the orbital contents (intraconal compartment). Hyperemia and conjunctival edema with proptosis are more prominent. Restriction in eye movement, ocular pain and severe visual loss are usually present.

Stage V ‒ Cavernous sinus thrombosis: Posterior extension of the phlebitis towards the cavernous sinus. The opposite eye may be involved. Prostration and meningismus are frequently observed.

##### Classification by Velasco and Cruz & Anselmo- Lima^112^

I ‒ Orbital cellulitis: Diffuse inflammation of orbital fat with a poorly defined transition between normal fat and high-density fat.

II ‒ Subperiosteal abscess: Well-defined purulent collection between the periorbita and at least one orbital wall adjacent to the sinus.

III ‒ Orbital abscess: Collection with heterogeneous density within the orbital fat, circularly delimited or not.

Regarding Chandler’s classification, criticism is particularly directed at stages I and V, as they are not properly orbital complications. In addition, it is not ordered by severity since orbital cellulitis can pose a greater risk to vision than a subperiosteal abscess. On the other hand, this classifications’ stages are based on the clinical history, allowing the doctor to suspect of the extent of the disease by the signs and symptoms. It allows a segmented type of therapy since cellulitis is usually treated medically and abscesses in general require surgical approach.^116^

The Velasco and Cruz & Anselmo-Lima classification is more concise, excluding preseptal infection, because it is an eyelid complication and not an orbital complication of ARS. It is a simple and objective classification with easy applicability by doctors from different areas (such as otolaryngologists, ophthalmologists and radiologists). Similarly, to Chandlers’ classification it is not ordered according to the severity of the condition.^112^, ^116^, ^117^

#### Treatment

Correct diagnosis is essential for treatment. Differentiating cellulitis from abscess, determining the site of infection and monitoring visual acuity are fundamental for therapeutic success. Orbital complications generally require hospital admission for intravenous antibiotic therapy and close ophthalmological monitoring. The intravenous antimicrobial therapy should cover aerobic and anaerobic pathogens. Different antimicrobial combinations can be used; ceftriaxone (for Gram negatives) associated with clindamycin or metronidazole (to cover anaerobes) and/or oxacillin or vancomycin (for coverage of *Staphylococcus aureus*). The duration of IV therapy can vary and may end 48-h after clinical improvement or up to 14-days. The choice of antibiotics and treatment duration should be in conformity with the Infectious Disease Department. Intravenous corticosteroid for orbital complications is controversial, without proven evidence, but usually prescribed to promote reduction of edema and proptosis.

Orbital cellulitis with absent vision alterations can be treated clinically, but very intense monitoring must be carried out. Immediate surgical intervention is needed if vision deterioration occurs or if there is no improvement in orbital signals after 48-h of intravenous antibiotic therapy. On the other hand, subperiosteal or orbital abscesses should be addressed surgically (with rare exceptions that will be described below), besides the opening of the affected sinuses. Medial abscesses are easily approached endoscopically, following resection of the lamina papyracea after adequate sinusectomy. External approaches for superior and lateral abscesses may also be necessary.

Indications for surgical intervention in orbital complications^4^, ^111^:

• Evidence of subperiosteal or orbital abscess on CT or MRI (except for medial subperiosteal abscess of small volume),
• Ophthalmoplegia.
• Reduced visual acuity, affected afferent pupillary reflex or impossibility to assess vision.
• Progression or no improvement of orbital impairments (diplopia, proptosis, edema, chemosis) after 48-h of intravenous antibiotic therapy.
• Progression or no improvement of the general conditions (fever, infection parameters) after 48-h of intravenous antibiotic therapy.

Small medial subperiosteal abscesses in children and adults can be managed medically in selected situations (exceptional conduct), expecting for evident clinical improvement within 48-h after the beginning of therapy.^118^, ^119^

These are criteria for conservative treatment of medial subperiosteal abscesses:

• Abscess width (axial CT section) ≤4 mm
• Normal vision, pupil and retina
• Absence of ophthalmoplegia
• Intraocular pressure < 20 mmHg
• Proptosis ≤ 5 mm.

According to BAR, when facing a strong suspicion of orbital complication, investigation with contrast CT and hospitalization under intravenous antibiotic therapy are essential. Surgical indication depends on the severity of the case and the clinical response to drug treatment.

### Intracranial complications

Intracranial involvement as a complication of acute rhinosinusitis is rare but a potentially fatal process. It is estimated that approximately 3% of hospitalized pediatric patients due to acute rhinosinusitis present an intracranial complication.^120^ Mortality from this condition decreased since the introduction of antibiotics and continued to decrease in recent decades. In 1980, estimated mortality was about 40%.^121^ Recent systematic reviews report mortality rate between 2% and 3%.^122^, ^123^

#### Diagnosis

Intracranial complications of acute bacterial rhinosinusitis include suppurative processes, such as epidural empyema or subdural and cerebral abscesses, and non- suppurative infections, such as meningitis, cerebritis and thrombosis of the superior sagittal or cavernous sinuses. The frontal sinuses are the most associated with intracranial suppuration, followed in order of frequency by the ethmoid, sphenoid and maxillary sinuses.^4^, ^124^

Cavernous sinus thrombosis represents only 10% of all intracranial complications.^125^, ^126^ The broad anastomotic venous system of the paranasal sinuses allows retrograde spread of infection to the cavernous sinus, causing sepsis and multiple involvement of the cranial nerves.^127^, ^128^, ^129^ The clinical picture, include proptosis, eyelid ptosis, diplopia, chemosis, impairment of the ocular motor nerves an involvement of the branches of the trigeminal nerve, ophthalmic nerve and the jaw, in addition to papilledema and signs of meningeal irritation associated with fever spikes and prostration. Symptoms begin in one side and may progress to the other.^4^, ^124^

Diagnosis of suppurative intracranial complications is initially accessed through contrast Computed Tomography (CT), recommended due to the lower cost and practicality in the emergency room. The sensitivity of contrast enhanced CT for an intracranial abscess is 95%–99%. However, in the first three days of abscess formation, contrast CT can be normal or with minimal changes. After this period, consolidation of the abscess occurs, appearing as a lesion with enhanced ring and a hypodense center. In up to 25% of cases, non-contrast CT may not identify the injury.^130^

Magnetic Resonance Imaging (MRI) is considered the “gold standard” due to greater sensitivity compared with CT, and when both are available, they complement each other. The CT is better to evaluate bone structures, while MRI is more effective for evaluating soft tissues.^131^

MRI has an additional diagnostic value excluding or confirming cavernous or sagittal sinus thrombosis. In cases with suspected meningitis and an intracranial abscess has been discarded by the imaging exam, lumbar puncture can be performed to determine the pathogens.^4^

#### Microbiota

The microbiology of the intracranial complication of ARS is distinct from ABRS. The latter is caused by bacteria that colonize the respiratory tract, such as *S. pneumoniae*, *Haemophilus influenzae*, *Moraxella catarrhalis* and, occasionally *Staphylococcus aureus*. In intracranial complications, *Streptococcus species* are the most prevalent, including *S. anginosus*, *S. intermedius* and *S. constellatus*. Among *Streptococcus species*, some studies have shown a change in this microbiota, and *S. anginosus* is emerging as the most common bacterial agent of synogenic intracranial abscesses.^113^, ^120^, ^129^, ^131^^,^^132^

*Streptococcus species* are a common commensal of mucosal sites, including the respiratory, genitourinary and gastrointestinal systems. The *S. anginosus* group have multiple virulence factors, responsible for its pathogenicity. One of these factors includes the production of hyaluronidase, which facilitates tissue liquefaction and abscesses formation. Furthermore, hyaluronidase contributes to the development of biofilms, which protect the microorganism from the host defenses and antimicrobials.^133^

Din-Lovinescu *et al* observed that 58% of patients with intracranial complications presented with polymicrobial growth in the paranasal sinuses or in other cultures, and 25% had anaerobes isolated in the cultures.^120^ Therefore, it is important to obtain multiple cultures, including sinonasal and intracranial samples, and blood cultures, to identify these polymicrobial involvements. Polymicrobial cultures are usually described as associated with longer hospitalization and longtime use of antibiotics.^134^

#### Treatment

Treatment of intracranial complications of ARS varies greatly, from intravenous antibiotics to sinonasal surgeries or open neurosurgical interventions. Intravenous antimicrobial therapy must cover aerobic and anaerobic pathogens, and several associations can be made, such as ceftriaxone (for coverage of Gram negative) with clindamycin or metronidazole (for anaerobic coverage) and/or ceftriaxone with oxacillin or vancomycin (for coverage of *Staphylococcus aureus*), until the antibiogram is available. If there is no evidence of resistance to third generation cephalosporin, vancomycin is no longer an option, and ceftriaxone and metronidazole may be appropriate unless risk factors for Staphylococcus infection Multidrug-Resistant Aureus (MRSA) are suspected.^135^, ^136^ Therapy should be carried out for at least 4-weeks after intracranial control is achieved.

Antibiotic therapy is often discontinued if there are significant improvements in the radiological image of the skulls and extended if there is evidence of persistence of infection.^136^ The choice of antibiotic and treatment time must always be shared with the hospital infection control department.

Unlike the well stablished clinical and surgical interventions in cases of intracranial infections, a particularly controversial issue is the role of sinonasal interventions in these intracranial complications of ARS. There are no specific guidelines about sinonasal surgical treatment, and most of the studies are retrospective reporting a great variety of procedures, based on anecdotal evidence, clinical experience and on the local resources’ availability.^124^

Milinis *et al* carried out a systematic review with 32 retrospective and observational studies and compared patients who underwent sinus surgery in addition to intervention neurosurgical, versus just sinus surgery or just neurosurgical intervention. The results were against the sinonasal intervention. However, they concluded that selection bias can be a determining factor in not having the adequate answer, since patients undergoing only neurosurgery could present more extensive collections or be considered too unstable for combined surgeries. Likewise, patients who were treated only with sinonasal drainage were probably more stable and with less severe intracranial abscesses.^124^ Some authors argue that sinonasal interventions are important in managing the primary source of infection, helping to treat the intracranial infection. Furthermore, sinonasal drainage can provide culture, particularly if no neurosurgery drainage is contemplated, bringing essential information to the long-term antibiotic therapy planning.^124^, ^136^

Another controversial topic is the treatment cavernous sinus thrombosis, regarding anticoagulation. Some experts recommend anticoagulation if the patient has no contraindications. Retrospective reviews are favorable, indicating lower morbidity and mortality when anticoagulation and antibiotics are used together to treat septic cavernous sinus thromboses, although there are no prospective clinical studies, due to the rarity of cases. Intravenous corticosteroid is often administered in conjunction with antibiotics, although their effectiveness has not been proven as well. Pathophysiological, intravenous corticosteroids can reduce inflammation and edema of the vessels around the cranial and orbital nerves. Endoscopic surgery on the affected sinus, in most cases the sphenoid sinus, is considered mandatory.^4^

Although it is unlikely that a clinical randomized trial for intracranial complications be performed due to the rarity of this condition and ethical issues, a well-designed multicenter prospective study could provide more assertive conclusions about these surgical interventions.

The ABR considers that, given the suspicion of an intracranial complication, a multidisciplinary assessment with imaging exams (computed tomography with contrast and/or magnetic resonance imaging) and hospitalization with intravenous antibiotic therapy is essential. Despite the lack of evidence, in an ideal scenario a combined otorhinolaryngological and neurosurgical surgical approach is ideal for an earlier and shorter recovery.

### Bone complications

These bone complications correspond to 3%‒10% of complications of ARS.^4^, ^137^ Occupies the third position in frequency the bone complications of ARS are the rarest, behind orbital (most common) and intracranial complications. Among bone complications, the most common is osteomyelitis of the frontal bone (generally in adolescents), followed by maxillary osteomyelitis (typically in childhood). Osteomyelitis of the frontal sinus walls can progress to a subperiosteal abscess producing soft tissue edema and bulging of the frontal region (Pott’s edematous tumor). Fistulation to the skin may occur, but it is uncommon.^3^, ^4^ Previous history of cranioencephalic trauma is considered a risk factor.

Orbital and intracranial complications may occur simultaneously.^4^, ^138^, ^139^ The progression of the infection towards the posterior wall of the frontal sinus directly or via thrombophlebitis of the diploic veins (no valves), explains the occurrence of intracranial complications. In this case, the patient may present with soft tissue edema (especially of the upper eyelid), high fever, intense headache, signs of meningeal disorders, nausea, diplopia, photophobia, lowering of the level of consciousness and focal neurological signs.

The diagnosis can be confirmed through a contrast CT.^4^ Magnetic resonance imaging can help the investigation of other complications. Technetium scintigraphy is helpful to confirm early diagnosis of osteomyelitis. Gallium scintigraphy can help monitoring and determining the resolution of the infectious process.^1^, ^140^

Treatment includes intravenous prolonged broadspectrum antibiotics (6–8 weeks) associated with surgical drainage and debridement of the sequestrum bone (fragments of necrotic bone that have detached from the healthy bone). Antibiotic therapy should preferably be guided by culture that often reveals a polymicrobial etiology. The most common microorganisms are *Streptococcus*, *Staphylococcus*, *Haemophilus influenzae* and anaerobes.^1^, ^137^, ^141^ For many years, an extensive external surgical approach was recommended, which certainly continues to make sense for the most serious cases. However, with the evolution of antibiotics and endoscopic sinonasal surgery, less severe cases (without intracranial involvement) have been the most frequent, generally treated endoscopically, whether or not associated with minimal external approaches.^4^, ^138^

BAR considers that if a bone complication is suspected, contrast CT investigation and hospitalization for prolonged intravenous antibiotic therapy/surgical debridement are essential.

## Particularities of ARS in children

### Epidemiology

The frequency of ARS in the pediatric population is greater than in adults. Although there is no concrete data about prevalence and incidence in the pediatric population, it is estimated that up to 7.5% of viral cases evolve into ABRS, and that these conditions are one of the main causes of antibiotic prescription in this age group.^8^, ^142^

### Paranasal sinuses in children

One of the main particularities in children is the development of the paranasal sinuses since not all of them are developed at birth. The frontal sinus, for example, begins its development around 4-years of age, and only 20%‒30% of children will have a frontal sinus radiologically visible at 6-years of life. The majority of children will only have CT frontal sinus detectable at 12-years of age. Regarding the sphenoid sinus, pneumatization begins to be visible on CT only at 7‒8-years of age and continues to develop until adulthood.^143^

The paranasal sinuses of greatest clinical importance in pediatric population are the ethmoidal and maxillary sinuses, already present at birth, although still very small. The ethmoidal sinuses become pneumatized quickly until the age of 7 and presents full development by 15–16 years of age. The maxillary sinuses, in turn, at 2-years of age have a volume of around 2 mL; at 9-years, 10 mL; and around 15 mL at 15–16 years of age. With pneumatization of the alveolar process, after the second set of teeth, at 12-years of age of age the maxillary sinuses expand mainly in the lower part. The floor of the maxillary sinuses, which in children is found at a higher level than the floor of the nasal cavity, gradually descends and, in adult life it will be about 4–5 mm below the nasal floor.^143^, ^144^

### Definition and classification of ARS in children

#### Diagnosis of ABRS in children

Clinical diagnosis of ABRS in children is not always easy. Many symptoms are common to other diseases in childhood, such as colds/flu/VARS and allergic rhinitis. Furthermore, there are limitations and difficulties in both, anamnesis and physical examination of the pediatric population.

Studies in children with ABRS show that the clinical picture often includes fever (50%–60%), rhinorrhea, (71%–80%), cough (50%–80%) and pain (29%–33%), in addition to nasal congestion/obstruction.^143^, ^144^, ^145^, ^146^ In young children, up to preschool age, painful symptoms are less frequent, while coughing is more common. In older children and adolescents, pain complaints become most frequent.^3^, ^4^, ^7^

According to the BAR, the diagnosis of ABRS must be considered when the patient presents symptoms with:

• **Significant worsening of the condition (increase in intensity/severity and/or signs of unilaterality) after an initial improvement in the first 5-days of illness-double worsening, OR**
• **Maintenance of the initial intense condition without gradual improvement after 14 days.**

#### Endoscopic examination in children

In addition to the signs and symptoms, the nasal endoscopy can be helpful to confirm the diagnosis of sinus involvement. However, purulent secretion draining from the middle meatus is indicative of sinus inflammation but does not distingue between viral or bacterial etiology. Besides, endoscopy is not always easy to perform in children. Furthermore, despite the high specificity for ARS (not specifically for ABRS), endoscopy presents a low degree of sensitivity, as the absence of discharge does not exclude the diagnosis of ABRS. Therefore, nasal endoscopy is not essential for the diagnosis of uncomplicated ABRS.^20^

#### Imaging study in children

There is practically a consensus among the guidelines that the diagnosis of ARS should be based on clinical criteria only, especially in children. X-Rays are not recommended for diagnosing uncomplicated ARS, since viral respiratory conditions in children often involve the paranasal sinuses. Children with symptoms of URTIs with at least six days of evolution usually present involvement of all sinuses, the maxillary, ethmoidal, sphenoidal and frontal sinuses, in order of frequency. Opacification is a nonspecific radiological finding and may be present in various situations such as viral, bacterial, allergic, tumoral processes, or even due to the lack of sinus development. Study of CT scans in children with clinical ARS demonstrated that even the most severe condition show significant improvement of image findings after two weeks.^147^ Therefore, recommendations for CT in acute sinus conditions should be reserved for patients with persistent symptoms after adequate therapy, and when a complication is suspected.

#### Differential diagnosis in children

The main differential diagnosis of ARS in children is acute infectious adenoiditis. This entity may present with very similar symptoms, including posterior discharge and cough. A high percentage of association is likely to exist between the two diseases, although this differentiation is very difficult in clinical practice. Studies show that 89.2% of children with symptoms for more than ten days, have ARS, and 19.2%, associated adenoiditis. Adenoiditis, alone, is present in about7% of children. Younger children (2‒5-years of age) tend to present an association with ARS/adenoiditis with higher frequency.^147^, ^148^

In clinical practice, the differential diagnosis is not always necessary since the treatment of the two conditions is the same. Another differential diagnosis is the presence of nasal foreign body. In these cases, the secretion is usually fetid and almost always one-sided.

### Bacteriology in children

A recent study shows that the most common bacterial agents in childhood ARS remain the same; *Haemophilus influenzae* (45%), *Streptococcus pneumoniae* (32%), *Moraxella catarrhalis* (16%), and *Chlamydophila pneumoniae* (13%). Culture revealed bacteria in 21 of the 31 cases (68%), with *H. influenzae* being the most frequent pathogen (42%).^149^

### Medical treatment of ABRS in children

Most ABRS in children are self-limited, resolving spontaneously, therefore, the initial conduct may be limited of careful observation, or just directed on relieving the most intense symptoms.^150^

#### Antibiotics in ABRS in children

Meta-analysis results suggest that the rate of resolution and improvement of ABRS between 7- and 15-days is greater with antibiotics when compared to placebo.^4^, ^150^ Despite the discussion about the real clinical impact of this statistical significance findings, in favor of an initial observation period without immediate antibiotic treatment is the well-known ARS self-limited natural history in childhood. So, it is also reasonable recommend an initial observation period without immediate antibiotic treatment, reserving immediate antibiotics treatment for moderate or severe cases, or even, in the presence of concomitant illnesses that could be exacerbated by ABRS, e.g., asthma and chronic bronchitis. The results of treatments with antibiotics may be more justified when there are indications of bacterial presence associated with the clinical picture. For example, Shaikh *et al* observed that patients diagnosed with S. pneumoniae and H. influenzae in the rhinopharynx before the treatment, showed significant better improvement with antibiotics compared to patients without the presence of these bacteria.^16^

There is no universal consensus on antibiotics to be used in ABRS in childhood. In general, amoxicillin is still considered an initial rational treatment.^7^ Alternatives include amoxicillin/clavulanate and second^1^ and third cephalosporins generation (orally)^1^, ^151^ that are considered good options against beta-lactamase producers and may also be indicated in cases of failure with the initial treatment. For children with ABRS who cannot tolerate oral medication, 3-doses/3-days of ceftriaxone 50 mg/kg IV (Intravenous) or IM (Intramuscular) may be an option, with sequential oral treatment. In specific cases of allergy to penicillin, macrolides, second and third generation cephalosporins are options to be considered (Table 4).

**Table 4:** Antibiotics of choice for ABRS in children.

Antibiotics of Choice	Posology	Duration	Special considerations
Amoxicillin	25‒50 mg/kg/d 8/8 h or 12/12 h	7 to 10 days	*Preferred in patients with suspected resistant pneumococcus.
	90 mg/kg/d* 8/8 h or 12/12 h	7 to 10 days	
Amoxicillin with clavulanic acid	25‒50 mg/kg/d 8/8 h or 12/12 h	7 to 10 days	*Indicated for beta-lactamase-producing bacteria and in doubled dose if resistant pneumococcus is suspected. Diarrhea in 1%‒10%.
	90 mg/kg/d* 8/8 h or 12/12 h	7 to 10 days	

Options for penicillin allergy	Posology	Duration	Special considerations
Axetilcefuroxime	25‒50 mg/kg/d 12/12 h	7 to 10 days	Amoxicillin/clavulanic acid-like spectrum.
Clarithromycin	15 mg/kg/d 12/12 h	7 to 10 days	Consider high resistance. Contraindicated for concomitant use of statins.
Cefdinir	14 mg/kg/d 24/24 h or 12/12 h	10 days	Third-generation oral cephalosporin with action on *S. pneumoniae*, H. influenzae and *M. catarrhalis*. It does not have the same spectrum as ceftriaxone, an option to axetilcefuroxime.

Options in case of failure	Posology	Duration	Special considerations
High dose amoxicillin	90 mg/kg/d 8/8 h or 12/12 h	7 to 10 days	Exceptional conduct without evidence of clinical efficacy. Observe increased gastrointestinal effects.
High dose amoxicillin with clavulanic acid	90 mg/kg/d8/8 h or 12/12 h, without increasing clavulanic acid.	7 to 10 days	
Ceftriaxone	20 to 80 mg/kg/d 24/24 h	3 to 5 days	Intravenous or intramuscular. After improvement after 3 to 5 days, continue oral treatment.
Clarithromycin	15 mg/kg/d 12/12 hs	7‒10 days	Contraindicated for concomitant use of statins. Consider for therapeutic failure due to atypical bacteria
Axetilcefuroxime	25‒50 mg/kg/d 12/12 hs	7‒10 days	Amoxicilin/clavulanic acid-like spectrum
Cefdinir	14 mg/Kg/d 24/24 hs or 12/12 hs	10 days	Third-generation oral cephalosporin with action on *S. pneumoniae*, *H. influenzae* and *M. catarrhalis*. It does not have the same spectrum as ceftriaxone. An option to axetilcefuroxime

#### Intranasal corticosteroids in children

Although the level of evidence is not high, the intranasal corticosteroid for up to three weeks, associated to antibiotics, appears to have advantages over to the treatment of ARS with antibiotics alone, especially regarding coughing and nasal secretion.^4^

Even though they are in common use in medical practice, due to the lack of evidence in the literature regarding their effectiveness, decongestants, oral or intranasal, antihistamines and even washing with saline solution are not routinely recommended in the treatment of children with ABRS.^4^

### Recurrent acute rhinosinusitis in children

There is no consensus on the definition of the Recurrent Acute Rhinosinusitis (RARS) in children. With complete remission between the crises, 4 or more episodes per year can be considered recurrence. It may seem obvious, but recurrent viral infection should always be rulled out. Besides that, as in chronic conditions, one must rule out some causes of systemic origin such as allergic conditions, immunodeficiencies, cystic fibrosis, gastroesophageal reflux, mucociliary diseases. Hypertrophy, not necessarily obstructive, of pharyngeal tonsils, should also be considered, due to the possibility of it acting as a reservoir of pathogens. Anatomical factors, generally considered to be of little relevance in the pathophysiology of ARS in children, should also be discarded (concha bullosa, septal deviations, etc.). CT, nasal endoscopy and/or MRI can help in the diagnosis of obstructive processes and malformations. The bacteriology is the same as ARS and, therefore, antibiotic treatment of the acute phase must follow the same precepts. In cases of recurrence, prophylaxis with antimicrobials should be reserved for exceptional cases, mostly in the presence of confirmed comorbidities, particularly immunodeficiencies. As general prophylactic measures, annual vaccination for Influenza and the pneumococcal vaccines are recommended. Bacterial lysates have shown to help preventing recurrences of viral and bacterial infections and can be an adjuvant in the control of RARS.^4^

## Investigation of Chronic Rhinosinusitis (CRS)

Currently, under the name chronic rhinosinusitis there are several diseases with distinct etiology, pathophysiology, prognosis and treatment. To ensure the best treatment for each patient, they must be individualized. Careful investigation, including anamnesis, physical examination and complementary exams are fundamental. A comprehensive assessment should include a detailed analysis of recent diagnostic criteria, such as the classification of CRS as primary versus secondary, the anatomical distribution (localized or diffuse) and endotype dominance (type 2 or not type 2). Pathophysiological mechanisms involved in the pathogenesis of CSR, including environmental, genetic and immunological factors should also be investigated. The dysfunctional interaction between the host and environmental stressors on the mucosal surface is crucial to the development of CRS and the consequent chronic inflammatory process.

### Clinical history

Clinical history is a fundamental part in CRS investigation. Time of onset, type and intensity of main symptoms (obstruction/nasal congestion, rhinorrhea, headache, olfactory changes, cough), worsening or improving factors, previous therapies, comorbidities, and many other items are all important and must be evaluated.

#### Environmental factors

Environmental factors, such as exposure to allergens, viruses, pollutants, tobacco smoke and chemical irritants, may exacerbate the symptoms of Chronic Rhinosinusitis (CRS) and contribute to chronic nasal mucosal inflammation. Air quality, both indoor and outdoor, significantly influences the incidence and severity of chronic rhinosinusitis.^152^

#### Systemic factors

Primary systemic diseases, such as asthma and autoimmune diseases, may be associated with upper airway chronic inflammation. Furthermore, secondary conditions, such as acquired immunodeficiencies, can predispose patients to chronic and recurrent infections.^153^ A detailed history of comorbidities and associated systemic issues is essential for an effective diagnostic and therapeutic approach.^3^

#### Genetic factors

Genetic factors play a significant role in the predisposition for CRS. Studies have identified specific genetic variants associated with the immune function and the epithelial barrier that can influence susceptibility to CRS. Future research is needed to elucidate the underlying genetic mechanisms and develop targeted therapies.^4^ Assessment of people in the family with similar symptoms helps to identify possible genetic causes.

### Assessment of severity of symptoms and quality of life

#### SNOT-22

**Fig. 2:**
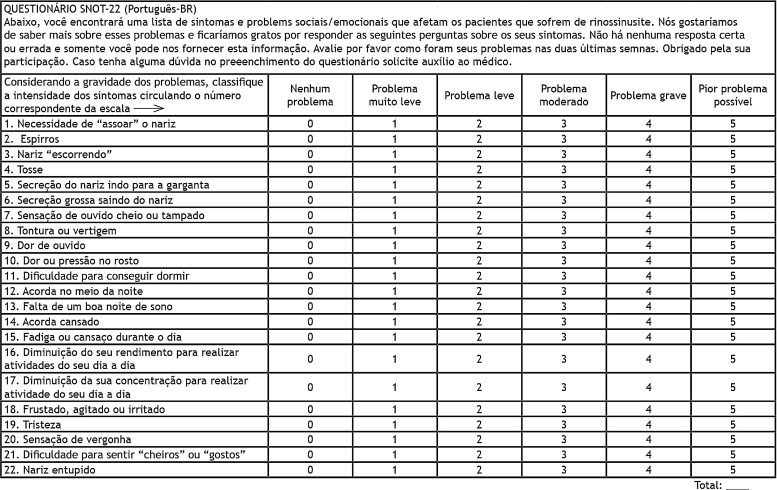
Questionnaire SNOT-22 validated to Brazilian Portuguese.^155^

There are several specific quality of life questionnaires to evaluate patients with sinonasal diseases. The SNOT22 (Sinonasal Outcome Test)^154^ was published in 2009 and is widely used worldwide, both in research and clinical practice and has been the preferred in Brazil. It has been translated and cross-culturally adapted in several languages. The Brazilian validation was carried out in 2011 by Kosugi *et al*^155^ It is a practical and self- applicable test to assess the impact caused by CRS on patient’s quality of life, as well as to evaluate the response to the treatment. The questionnaire includes 22 symptoms, and the patient grades the severity of each symptom on a scale of 0–5 (0-no problem, 1-very mild problem, 2-slight, 3-moderate, 4-severe, 5-worst possible). Next, the score must be added up and may vary from 0 to 110 (Fig. 2). The cutoff score related to the severity of the disease is still controversial. EPOS 2020 considered that a value of ≥40 represents a significant impact on quality of life.^4^ The Minimal Clinically Important Difference for SNOT-22 (MCID) is 9-points, and it is consistent in the current literature, so, a variation of 9-points can be considered relevant.^4^, ^156^

In the Brazilian validation the MCID was 14-points. However, the analysis of the normal population presented scores varying between 0 and 8-points. Thus, 9-points could be considered an MCID as well, similarity to studies in other countries.^155^, ^157^, ^158^

#### Visual Analogue Scale (VAS)

**Fig. 3:**
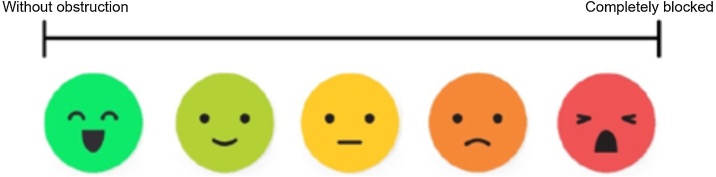
Visual Analogue scale in “ruler” and “emojis”.

Visual Analogue Scale (VAS) is a tool to assess the severity of the patient’s symptoms. Its main advantages are the agility and practicality in application. Furthermore, results are correlated with SNOT-22.^4^ A bar of 10 cm is presented to the patient, who is asked to point out on the bar, the level of the symptom from 0 to 10. Each end of the bar represents an extreme of the symptom. (Example: on one side “without obstruction” and on the other “completely blocked” (Fig. 3). In patients with chronic rhinosinusitis, it is reasonable to routinely evaluate the main symptoms in each appointment: nasal obstruction/congestion, nasal pain, rhinorrhea, change in smell and facial pain.

### Olfactory tests

Testing olfactory function plays an import role when prescribing different medications for CRS.

Including biologics, determining the continuity or not of these treatments. The olfaction is often impaired in CRS, especially in patients with CRS with nasal polyps (CRSwNP), due to both, obstructive or inflammatory reasons.^159^, ^160^ On one hand, the olfactory molecules cannot reach the olfactory epithelium; on the other hand, the olfactory epithelium functions poorly due to an impaired signaling caused by the inflammatory response.

A recent cross-sectional observational study aimed to determine the inflammatory endotype pattern in Brazilian patients with Chronic Rhinosinusitis (CRS) and to correlate it with olfactory function, in addition to assess the clinical severity of the disease. Seventy-three patients with CRS were recruited, and compared to patients with non-type 2 CRS, those with type 2 CRS had a higher prevalence of nasal polyps (93% vs. 12.5%), asthma (40.3% vs. 12.5%) and respiratory disease exacerbated by nonsteroidal anti-inflammatory drugs (AERD, 17.5% vs. 0%). Type 2 patients also showed significantly lower scores on olfactory tests, indicating reduced olfactory function, and higher scores on the SNOT-22, Lund-Kennedy and Lund-Mackay, indicating greater clinical severity.^161^

Patient perception of the olfactory function serves as an effective indicator to evaluate the treatment response; however, it does not replace the psychophysical assessment of smell. If patients with CRS are experiencing a normal sense of smell or just mild hyposmia, this is a good sign that treatment is effective. In Brazil, three validated tests are available for patients with Chronic Rhinosinusitis (CRS). These tests, described below, are recommended by this Consensus for both pre- and posttreatment to manage the disease in an effective way.

#### Digital odor identification Test-Multiscent-20

The Multiscent-20 is a portable device designed to identify odors, with an advanced system of aromas storage and release. It includes a touch screen and a system of microcapsules that presents up to 20 different odors through a controlled flow of dry air that is delivered from the device. Capsules are loaded through a port insertion on the back of the device. The software automatically manages the presentation, control and recording of answers.^162^

The test employs a forced-choice paradigm with four alternatives and starts with instructions provided by an on-screen avatar. Participants must identify 20 different odors throughout the test. They are instructed to sit comfortably and hold the device 10 cm away from the face when asked to release the odor. During the test, the screen displays “THIS SMELL IS SIMILAR TO” followed by four options response form, and participants are advised to read all options carefully before pressing the “RELEASE SMELL” button. When pressing this button, a small opening at the top of the device releases the odor for 5 s, with the option to press the button up to twice if necessary. After smelling the odor, participants select the answer option that best corresponds to what they felt and continues to press the “NEXT” button. If none of the options match exactly to the perceived odor, they are encouraged to choose the closest one. At the end of the test, the number of correct answers is revealed to the participant as part of the evaluation process.

Multiscent-20 scores are based on the number of correct answers. The olfactory function is classified as normosmia (≥15-points), hyposmia (14 to 11-points) and anosmia (≤10-points).

#### Connecticut Sensory Chemo Clinical Research Center (CCCRC) Test

The Connecticut Chemosensory Clinical Research Center (CCCRC) Test evaluates olfactory function through two main tests: olfactory threshold and odor identification.^163^ These tests provide quantitative and qualitative assessments of a person’s sense of smell. A unique feature is its ability to independently assess each nasal cavity, distinguishing between olfactory impairments on each side with minimal cost.

Olfactory Threshold Test: The olfactory threshold test uses seven concentrations of butanol (n-butyl alcohol) in vials numbered 1–7, from highest to lowest concentration. Bottle 8 contains odorless distilled water for control. The test begins with the presentation of two vials—one with distilled water and the other with butanol. The person identifies which bottle contains the odor. Starting with bottle 7, if the person detects the odor, their olfactory threshold score for that nostril matches the number on the bottle. Otherwise, progressively more concentrated vials are presented up to identification or up to vial 1. A score of 0 indicates an inability to detect even the strongest concentration. This process is repeated for the other nostril.

Odor Identification Test: Eight substances—coffee, cinnamon, baby powder, peanut, chocolate, neutral soap, mothballs and menthol are presented in opaque bottles. The person, guided by a pre-provided list, identifies each substance without seeing its contents. Scores range from 0 to 7 for each nostril based on correct identifications, with menthol excluded from the score but used to assess trigeminal nerve function.

Score: The final score for each nostril combines the average of the olfactory and identification threshold test scores, resulting in a score of 0–7 points per nostril. The final score is the average of the threshold test with the identification test, considering the following values: Normal: 6.0–7.0; Mild hyposmia: 5.0–5.75; Moderate Hyposmia: 4.0–4.75; Severe Hyposmia: 2.0–3.75; Anosmia: 0–1.75.

This comprehensive CCCRC approach provides a detailed assessment of olfactory function, suitable for clinical and research settings.

### University of Pennsylvania Odor Identification Test (UPSIT®)

The University of Pennsylvania Odor Identification Test (UPSIT®) involves the identification of 40 different odors distributed in 4 booklets, each containing 10 pages. To activate the test, the patient scrapes the brown strip at the bottom of each page to release the odor. Holding the booklet approximately 1.0 cm from the nose, the patient selects a correct answer between four provided options. Answers can be marked directly on the test booklet or on a separate sheet for later scoring. The total score is based on the number of odors correctly identified, which determines the classification of olfactory function as normal or impaired.^164^

### Nasal endoscopy (Nasal polyp scale and Lund-Kennedy scale)

Nasal endoscopy is mandatory in all stages of care for a patient with chronic rhinosinusitis: diagnosis, evaluation of response to treatment, care, and postoperative follow-up. There are several methods to systematize its use and registration. Among the most widespread are the nasal polyps scale^165^ and, the Lund-Kennedy endoscopic scoring system.^166^ Their routine use allows the otorhinolaryngologist to measure and compare the patients condition at each visit.

#### Nasal Polyps Scale (NPS)

**Fig. 4:**
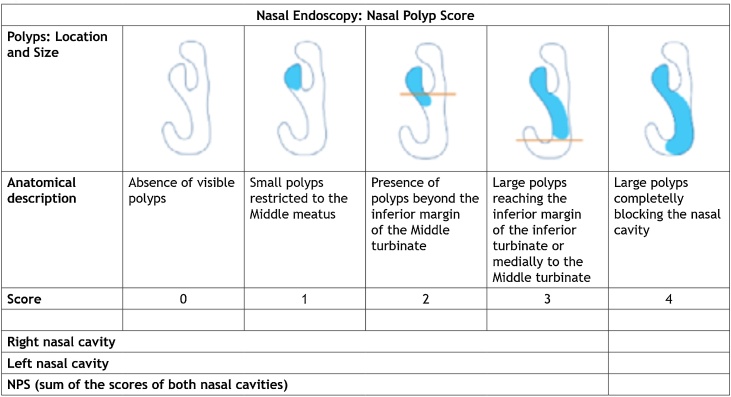
Nasal Polyps Score.

This evaluation system is very simple and reproducible. Therefore, it is widely used in clinical practice and in research. Only the presence and size (and/or location) of the polyps are considered. Each nasal cavity can range from the absence of polyps (score 0) to polyps completely obstructing the nasal cavity (score 4). The result is obtained by adding the scores of the two nasal cavities, which can vary from 0 to 8 (Fig. 4).^165^, ^166^

#### Lund-Kennedy endoscopic scoring system

It is a more comprehensive endoscopic evaluation system, as it considers the evaluation of polyps, edema, secretion, adhesions/synechiae and crusts.^166^ There is also a simpler modified version (considering only polyps, edema and secretion)^167^ which has greater reproducibility and correlation with SNOT-22.^168^ Thus, it is a very interesting system for office routine. Each of these characteristics must receive a score ranging from 0 to 2 in each nasal cavity.

### Biopsy

Biopsy is an essential diagnostic tool in the investigation of Chronic Rhinosinusitis (CRS). It helps to confirm the diagnosis and the endotyping of inflammatory diseases, and it is important for research purposes. Biopsy provides non-macerated tissue, suitable for histopathological analysis, allowing the exploration of differential diagnoses such as inflammation, Respiratory Epithelial Adenomatoid Hamartoma (REAH), infection, granuloma/vasculitis, and tumors.^169^ It also helps to confirm potential etiological or pathogenic mechanisms, such as the type and severity of inflammation, cellular composition (e.g., eosinophils), and the presence of bacterial and fungal elements, as well as determining the nature of the infection (e.g., invasive or non-invasive fungus) and determining potential therapies, such as biologics.^4^

Nasal biopsies can be performed in an outpatient setting under local anesthesia or in the operating room. The choice of biopsy site and method depends on the reason and the required sample size.^170^ For research purposes, biopsy should be easily obtained, painless, and with minimal complication rates. For clinical diagnosis, the sample must be large enough to allow adequate analysis, but without causing excessive discomfort to the patient. It is important that the material is not crushed during the collection, by using specific forceps, such as Fokkens forceps or fenestrated forceps.^171^ Studies have shown that biopsies can be safely performed on the olfactory mucosa without affecting nasal function or changes in the sense of smell.^172^ Subphenotyping and endotyping according to the eosinophilic infiltration and other histological criteria are gaining importance.^173^ This fact should result in the development of institutional protocols for sampling storing and processing in collaboration with histopathologists. Chronic Eosinophilic Rhinosinusitis (CRSe) requires quantification of the number of eosinophils per High-Power Field (HPF), with cut-off values varying in the literature.^174^ Systematic studies have shown that high eosinophilic infiltration is associated with greater disease recurrence, being a useful marker for the diagnosis of CRS.^174^

Romano *et al* conducted a multicenter study in Brazil to evaluate the inflammatory profile of patients with chronic rhinosinusitis with nasal polyps (CRSwNP). The results indicated that type 2 inflammation, when characterized by more than 10 eosinophils per high power field in polyps, according to EPOS criteria, is predominant in about 80% of patients. However, when cytokine concentrations were evaluated, this group had a mixed inflammatory profile, with T1, T2, and T3 characteristics. The clustering of the findings allowed the identification of 2 groups, one more inflamed and one less inflamed, and the best practical criterion to distinguish the groups was tissue eosinophilia greater than or equal to 43 per HPF. This inflammatory profile was associated with clinical severity and worse prognosis.^175^

The findings of the Brazilian study reinforce the need of an individualized approach when treating chronic rhinosinusitis, where therapy is tailored based on the patients’ individual inflammatory profile. This type of approach can result in better clinical outcomes and more efficient treatment of the disease.^175^

### Culture of sinus secretion

When managing CRS, culture of sinus secretion can help the identification of bacterial and fungal pathogens, allowing more targeted and potentially more effective treatment. Culture also helps to differentiate between acute and chronic bacterial infections, as well as providing important information about antimicrobial resistance.^176^, ^177^

The collection of sinus secretions can be carried out through different methods, varying according to specific indications:

a) Maxillary Sinus Aspirate: the puncture of the maxillary sinus, performed under local anesthesia through the inferior meatus or canine fossa, is traditionally considered the gold standard for obtaining representative samples of sinus secretions. Although it is an effective procedure, it is often associated with discomfort and rarely with potential risks such as orbital and dental injuries.^176^, ^177^, ^178^
b) Endoscopically Directed Middle Meatal cultures (EDMM): Endoscopically guided middle meatus swabbing offer a less invasive alternative for collecting sinus samples. Studies shown a high rate agreement between EDMM and maxillary sinus aspirates, making this technique a common practice for microbiological evaluation of patients with rhinosinusitis.^179^, ^180^
c) Direct Sinus Lavage: Direct sinus lavage, performed through an endoscopically placed maxillary sinus catheter, can improve bacterial yield and increase the recovery of anaerobic pathogens. This technique can be facilitated by balloon devices, which have built-in catheters for drainage and antibiotic irrigation.^181^

#### Clinical applicability

Sinus secretion culture is especially useful to identify specific pathogens. In the Brazilian population the most common are *Staphylococcus aureus*, *Pseudomonas aeruginosa* and *Enterobacteriaceae*.^182^ Accurate identification of pathogens allows you to choose appropriate antibiotics, improving the treatment efficacy and reducing antimicrobial resistance.^176^

#### Challenges and limitations

While culture of sinus secretion is a valuable diagnostic tool, there are challenges and limitations to consider:

• Nasal Contamination: Samples obtained by nasal swabs can be contaminated by the local flora, resulting in less representative cultures. This can be minimized using endoscopically directed collection techniques.^179^
• Slow-Growing Pathogens: Traditional culture techniques may not identify slow-growing pathogens or those that require specific growing conditions. Advanced molecular methods, such as next-generation sequencing, can provide a more comprehensive identification of the microorganisms.^183^

The ABR recommends that microbiological evaluation should be performed in the following cases: severe rhinosinusitis, nosocomial rhinosinusitis, immunosuppressed patients, locoregional complication, poor response to antibiotic treatment, clinical trials and epidemiological studies.

### Allergological evaluation

There are several studies evaluating the correlation between allergy and Chronic Rhinosinusitis with (CRSwNP) or without Nasal Polyps (CRSwNP), showing controversial results. While some point to a possible association, others do not. However, the level of evidence on the subject is low.^4^

Recent studies suggest that different phenotypes/ endotypes of CRSwNP may have links with Allergic Rhinitis (AR) as the central atopic compartment disease. Thus, AR could be considered a disease-modifying factor in CRSwNP.^3^ However, the evaluation of AR in patients with CRSwNP is optional,^3^ but may be recommended in special cases, notably relapsed cases or cases of surgical reintervention.^4^

Allergen sensitization is measured by “in vivo” methods such as the prick test, or “in vitro” methods such as specific serum IgE measurement or by the basophil activation test. In exceptional cases, where occupational sensitization is suspected, nasal or conjunctival provocation testing may be performed.^184^ “In vitro “tests have the advantage of not suffering interference with the use of medications, and in addition, do not expose the patient to systemic allergenic reactions.^4^

Nasal cytology is used to assess the cellularity of the mucus. Collecting techniques may vary between nasal washing and nasal brushing. The material obtained by nasal brushing can also be used in electron microscopy to evaluate the presence of primary ciliary dyskinesia.^4^

#### Laboratory tests

Blood tests in CRS are usually for investigation of vasculitis, humoral immunodeficiencies and more currently to detect biomarkers of the type 2 reaction, such as total IgE and serum eosinophils.^4^

Eosinophils are characteristic cells of type 2 reaction, and their levels correlate with tomographic scores of Lund-Mackay and Lund-Kennedy endoscopic images in patients with CRSwNP.^4^ They are considered markers of type 2 reaction when the serum levels exceed 150 cells/ mm^3^.^185^

#### Aspirin (acetylsalicylic acid) susceptibility test

A clear history of multiple reactions accompanied by respiratory symptoms occurring within 2 h of ingestion of a Nonsteroidal Anti-Inflammatory Drug (NSAID) in a patient with adult-onset asthma and recurrent CRScPN may be sufficient to diagnose aspirin and/or NSAID-Exacerbated Respiratory Disease (ERD). In cases of diagnostic doubt, a challenge test with aspirin or the drug implicated in the reaction is necessary to confirm the diagnosis. On the other hand, it is important to emphasize that the absence of a history of respiratory reactions to NSAIDs in a patient with asthma and CRSwNP does not exclude the presence of hypersensitivity.^186^, ^187^

Aspirin/NSAID Oral Challenge (OCT) testing is the gold standard for diagnosing NSAID hypersensitivity, as it mimics natural exposure to the drug. Despite the advent of new diagnostic tools, such as the Basophil Activation Test (BAT), these should not replace challenging tests and are not recommended for the routine diagnosis of ASRD.^186^, ^188^, ^189^

Other routes, such as intranasal and inhalation, are less sensitive, but safer and faster alternatives, especially for patients in whom oral challenge is contraindicated. The main indications and contraindications for aspirin challenge tests are in the Table 5.

**Table 5:** Indications and contraindications for aspirin challenge tests in patients with ASRD.

Test type	Indications
Oral	Confirmation (or exclusion) of NSAID hypersensitivity in patients with a doubtful history.
	Verification of negative results in inhaled or intranasal tests
	Evaluation of the provocative dose of aspirin prior to oral desensitization
Intranasal	Diagnosis of NSAID Hypersensitivity in Patients with contraindications to oral or inhalational testing
	Diagnosis of ARSD in patients with upper airway symptoms of NSAID Hypersensitivity

Test type	Contraindications
Oral or inhaled	History of anaphylactic reactions precipitated by aspirin or other NSAIDs (alternatively, an intranasal challenging should be considered)
	Uncontrolled asthma
	FEV1 < 70% of the expected value
	History of chronic renal failure or gastrointestinal bleeding
	Respiratory tract infection or asthma exacerbation within 4-weeks prior to testing- Pregnancy
	Current treatment with β receptor blocker.
Intranasal	Nasal disease that interferes with nasal function challenge
	Upper respiratory tract infection within 4-weeks prior to testing.

Adapted from Kowalski *et al*.^186^

Different protocols for aspirin challenge testing are recommended.^186^, ^190^, ^191^ These procedures must be performed in a specialized clinical setting, by allergy specialists and trained healthcare staff. After the test is completed, the patient should remain in the clinic for a few hours or the entire day, depending on the clinical evaluation and the severity of the reaction.

### Imaging studies

Computed Tomography (CT) is fundamental when investigating CRS. Plain radiography is not recommended for this purpose by most of the practice guidelines.

The usefulness of Magnetic Resonance Imaging (MRI) in diagnosing CRS is restricted to certain situations, such as concerns about involvement of the skull base or inflammation in the sinonasal region related to tumors.

CT imaging offers higher sensitivity compared with nasal endoscopy. It is worth mentioning that a CRS patient has at least 3-months of clinical history and will probably undergo at least 3-more months of clinical treatment. A diagnostic error in this situation would delay at least 6-months the correct treatment. However, concerns persist regarding radiation exposure.

The ABR recommends performing at least one CT scan in the investigation of patients with CRS symptoms, preferably between acute exacerbations. In addition, the ABR does not recommend serial CT scans to assess response to treatment.

It is recommended to assess the severity of CRS with CT scans using various scoring and staging systems. A widely accepted system is the Lund-Mackay system, which evaluates the degree of opacification of the maxillary, anterior and posterior ethmoidal, frontal, and sphenoidal sinuses (0 = none; 1 = partial; 2 = complete), as well as scoring the blockage level of the osteomeatal complex (0 = unblocked or 2 = blocked). This system generates a maximum score of 24 or 12 per side, aiding in the comprehensive assessment and management of CRS (Table 6).^191^

**Table 6:** Lund-Mackay scale for tomographic assessment.^192^

Sinus	Right side score	Left side score
Maxillary	0, 1 ou 2	0, 1 ou 2
Anterior Ethmoidal	0, 1 ou 2	0, 1 ou 2
Posterior Ethmoidal	0, 1 ou 2	0, 1 ou 2
Sphenoidal	0, 1 ou 2	0, 1 ou 2
Frontal	0, 1 ou 2	0, 1 ou 2
Ostiomeatal Complex	0 ou 2	0 ou 2
Total	0 a 12	0 a 12

### Comorbidities

#### Allergic rhinitis

Allergic Rhinitis (AR) is defined as an IgE-mediated, type I hypersensitivity response triggered by exposure to allergens and resulting in inflammation of the nasal mucous membranes and typical symptoms that include: rhinorrhea, nasal congestion, itching, and sneezing.^193^

Upper airway diseases present a variable pattern of common symptoms, making it difficult to differentiate the diagnosis of Chronic Rhinosinusitis (CRS) from AR and non-allergic based solely on their clinical presentation.^4^ In addition, when the diagnosis is complemented with nasal endoscopy and CT scan, not all patients with symptoms that meet CRS criteria have evidence of sinus disease.^194^

Despite the lack of an uniform definition for AR in epidemiological studies, different authors have demonstrated a higher risk of CRS in individuals with AR, asthma, and other allergic conditions.^4^, ^5^, ^195^, ^196^ Moderate or severe nasal obstruction, any decrease in smell/taste, or positive nasal endoscopy in patients with a presumed diagnosis of allergic rhinitis should undergo further evaluation for CRS in order to prevent delayed treatment.^195^, ^197^

Studies using different methods have demonstrated connections between Th2 inflammatory cytokines and the occurrence of nasal diseases such as AR, RSCsNP and RSCwNP. The participation of Interleukin (IL-4), IL-5, IL13, IL-17, INF-g, TGF-β and matrix metalloproteinases in the development of NP and CRS have been documented.^198^, ^199^ AR may be associated with CRS in a variety of clinical settings. Although characterized by persistent inflammation of the sinonasal cavity, the type of inflammation in CRS varies according to the main subtypes. CRCwNP is usually associated with type 2 inflammation, whereas CRSsNP has a lower predominance of this type of inflammation. Thus, AR, whose main pathophysiological characteristic is type 2 inflammation, is considered a potential factor to CSR development.^4^

To date, there are no controlled studies examining the role of AR in the development of CRSsNP, nor demonstrating that the treatment of allergic diseases alters its progression, or vice versa.^200^ Even so, although this correlation is not well established, some authors recommend skin allergy tests or dosage of specific IgE for aeroallergens in CRSsNP, due to the theoretical benefit of identifying and treating allergic comorbidities of these patients.^193^

In this context, a recent study demonstrated that sensitization to multiple allergens, such as dust mites and pollens, contributes to the severity of patients with CRS, particularly decreasing the olfactory function and worsening radiological scores. These findings underscore the importance of considering the pattern of allergen sensitization when assessing the severity of CRS and its potential progression.^201^

The pathogenesis of CRSwNP is strongly associated with type 2 inflammation, with high levels of eosinophils, mast cells and basophils within these structures.^4^ Hypersensitivity to mites, cockroaches, and fungi has been associated with CRSwNP. However, clinical evidence on the association between AR and CRSwNP remains controversial, with studies for and against this finding.

In summary, the association between AR and CRSwNP remains unclear due to the lack of robust scientific evidence and, above all, due to variations in the definitions and classifications of these conditions in epidemiological studies.^200^

As the understanding of CRS endotypes and their inflammatory patterns evolves, it is important to specify the relationship of AR to the Central Compartment Atopic Disease (CCAD) subtype. Another condition associated with the presence of IgE is Allergic Fungal Rhinosinusitis.

#### Asthma

Asthma is a heterogeneous disease characterized by chronic inflammation of the airways, bronchial hyperreactivity, and variable airflow obstruction. Asthma symptoms include wheezing, dyspnea, chest tightness, and coughing, which vary over time in frequency and intensity. The prevalence of asthma ranges from 1% to 29% in different populations.^202^

The relationship between asthma and CRS has been widely studied, and a fourfold higher risk of CRS has been described in patients with asthma.^203^ It is estimated that 22% to 45% of patients diagnosed with asthma have CRS as a comorbidity, whereas in the general population the prevalence of CRS ranges from 5% to 10%.^4^, ^204^

In patients with CRS, the prevalence of asthma ranges from 4% to 44% in different studies.^205^, ^206^, ^207^, ^208^, ^209^, ^210^, ^211^ In both pediatric and adult patients with CRS, the most commonly reported comorbidities are allergy and asthma.^210^ In addition, CRS is associated with increased asthma severity, especially in patients with CRSwNP.^212^, ^213^, ^214^, ^215^, ^216^, ^217^ Regardless of the presence of polyps, CRS increases the risk of asthma, and asthma increases the risk of CRS, especially in young males.^218^

Several studies have shown that CRS contributes to the triggering of asthma exacerbations, and that asthma symptoms can be better controlled after medical or surgical treatment of CRS.^219^, ^220^, ^221^ A recent systematic review showed that patients with CRS and asthma had improvement of quality of life related to the disease after treatment. However, patients with CRS and asthma had a worse response to the clinical treatment of rhinosinusitis, indicating that the association of the two diseases makes management even more difficult.^212^

In the evaluation of patients diagnosed with asthma, especially those with difficult-to-control asthma or severe asthma, risk conditions for exacerbations, including CRS, should be investigated. Factors related to environmental exposure, psychosocial problems, inflammatory biomarkers, and characteristics related to disease severity and drug treatment should also be evaluated (Table 7).

**Table 7:** Risk factors associated to asthma exacerbations.

**Comorbidities and other clinical conditions:** Chronic Rhinosinusitis, obesity, Gastroesophageal Reflux, Food allergy, pregnancy
**Exposure:** Smoking, electronic cigarettes, exposure to akkergens, air pollution
**Psychosocial problems:** Psychic and socioeconomic disorders
**Pulmmonary function:** VEF1 < 60%, High FeNO
**History of severe exacerbation:** Intubation or ICU hospitalization, more than 1 severe exacerbation in the past year
**Overuse of short-acting bronchodilator:** More than 3-bottles/year
**Inappropriate use of inhaled corticosteroid:** Insufficient dose, poor adherence, incorrect inhalational technical
**FEV1:** forced expiratory volume in in the first second;
**FeNO:** exhaled fraction of nitric oxide;
**ICU:** intensive care unit

The high prevalence of CRS in patients with asthma has been attributed to the anatomical and functional similarities between the upper and lower airways, and to the shared pathophysiology of both diseases.^204^, ^222^

Since the introduction of the “united airway” concept, the correlation between asthma, CRS, and AR has been widely studied.^223^ Evidence of immunopathological mechanisms shared by CRS and asthma reinforced this concept.^224^ Nasal biopsies from patients with CRS show epithelial damage and basement membrane thickening.^225^ It has also been shown that nasal provocation tests induce bronchial inflammation and bronchial challenging tests induce nasal inflammation, indicating a bidirectional relationship between the upper and lower airways.^226^, ^227^

Currently, several mechanisms (endotypes) and distinct clinical presentations (phenotypes) related to asthma are recognized. Phenotypes are defined by demographic, clinical, and/or pathophysiological characteristics, the most common include allergic asthma, nonallergic asthma, cough variant asthma, adult-onset asthma, asthma with persistent airflow limitation, and asthma with obesity. Among these, the most common phenotype is allergic asthma, which usually begins in childhood and is associated with a personal or family history of allergic diseases.^202^

Asthma endotypes are categorized as T2-high and T2-low based on the presence or absence of T-helper 2 lymphocyte-mediated inflammatory response.^202^ The T2-high endotype is characterized by the production of the cytokines IL-4, IL-5 and IL-13 and the participation of Innate type 2 (ILC2) cells.^228^, ^229^ When the epithelium and dendritic cells are exposed to allergens, viruses, bacteria, and irritants, alarmins are produced (IL-33, IL-25, and Thymic Stromal Lymphopoietin ‒ TSLP) and other cytokines that initiate the inflammatory cascade. The interleukins IL-4 and IL-13 regulate the exchange of B lymphocyte isotypes for IgE production, while IL-5 regulates the recruitment and activation of eosinophils, and stimulates their production by the bone marrow besides delaying apoptosis.

T2 inflammation is usually characterized by high numbers of eosinophils, increased Fraction of Exhaled Nitric Oxide (FeNO), and may be accompanied by elevated IgE. Activated eosinophils release Eosinophilic Cationic Protein (ECP), Major Basic Protein (MBP), and eosinophilic peroxidase.^228^, ^229^ These mediators and cytokines attack epithelial cells, induce their desquamation, increase mucus production, and cause edema and bronchoconstriction. In addition, they contribute to the remodeling of the airways. Peripheral eosinophils, sputum eosinophils, and FeNO are considered biomarkers of T2 inflammation. In the low- T2 endotype, Th17 lymphocytes are involved with the production of IL17, IL1B and IL-23 with an increase of neutrophils in the airways.^202^, ^229^

The growing understanding of these mechanisms related to T2 inflammation coincides with the advent of targeted therapies, which target specific inflammatory cells and mediators, based on biomarkers that reflect the underlying endotype. Several therapeutic targets have been explored, expanding the treatment options for severe asthma and CRSwNP.

Asthma and CRS are diseases that often coexist, especially in patients with severe asthma. These patients have more frequent exacerbations, poorer symptom control, and impaired quality of life. Identifying and treating these conditions can significantly contribute to better disease management, reducing costs, and improving the quality of life. The available evidence reinforces the importance of an integrated and multidisciplinary care approach for patients with asthma and CRS, emphasizing the need for targeted and personalized treatment.

## Chronic Rhinosinusitis without Nasal Polyps (CRSsNP)

### Anatomical and iatrogenic alterations

#### Anatomical alterations

The paranasal sinuses are aerated cavities in the anterior skull that communicate with the nasal cavity through small ostia. Inside most of the paranasal sinuses, mucociliary beating occurs unidirectionally to these small orifices.^230^, ^231^, ^232^ Factors such as deficiency in ciliary beat, thick mucus, and narrow ostia, alone or together, may favor the irreversible histological alterations of the mucosa that characterize CRS.^230^, ^231^, ^232^ In addition to inflammatory/infectious causes, there is a hypothesis that anatomical alterations may favor the obstruction of these ostia. Although there is no scientific evidence regarding causality, there are reports of the association of these alterations with CRS and/or ARRS in some patients. The recognition and interpretation of these anatomical alterations have relevant practical applications in the daily routine of otorhinolaryngologists since they can influence the decision to recommend surgical treatment.^4^ The Ostiomeatal Complex (OMC) is a narrow anatomical area of great importance for the drainage of the frontal, maxillary, and ethmoid sinuses.^230^, ^231^, ^232^ It corresponds to the functional unit that is composed of the ostia of the maxillary sinuses, anterior ethmoidal cells and their ostia, ethmoid infundibulum, semilunar hiatus, and middle meatus.^233^, ^234^ Anatomical alterations that may limit the drainage patency of OMC:

a) Bullous middle turbinate: it is associated with pneumatization of the bony plate of the middle turbinate. It presents a wide variation in pneumatization, which is the cause of its high prevalence. This can obstruct the OMC and the nasal airflow.
b) Paradoxal Middle turbinate: It is a Middle turbinate with paradoxical curvature in relation to the lateral wall of the ethmoid, which may cause narrowing of the OMC.
c) Nasal septum deviation: Depending on the location, some deviations can also narrow the OMC region.
d) Haller’s cell: These are ethmoidal cells that develop towards the roof of the maxillary sinus, adjacent to and superior to the maxillary sinus ostium. Very pneumatized or diseased cells can lead to maxillary rhinosinusitis due to the blockage of the sinus drainage.
e) Variations of the uncinate process: The uncinate process is an important bony structure of the lateral nasal wall. Adjacent to the ethmoid bulla, defines the semilunar hiatus that forms an exit to a recess, the ethmoid infundibulum, which is directed anteriorly and inferiorly. The maxillary sinuses ostium is located on the posterior aspect of the infundibulum. In endoscopic sinus surgeries, usually, the first procedure to access the maxillary sinus is the uncinectomy. When the free margin of the uncinate process is enlarged or deformed, it can compress the infundibulum, disturbing sinus ventilation. Notably, lateral displacement and hypoplasia of the uncinate process have been associated with maxillary sinus hypoplasia.^233^, ^234^, ^235^

The changes described above have been suggested as potential risk factors for the development of CRS. Some authors postulate that anatomical variations of the paranasal sinuses may contribute to ostial obstruction; however, several studies demonstrate that the prevalence of anatomical variations is not more common in patients with rhinosinusitis or polyposis than in the general population. There is no evidence for a causal correlation between nasal anatomical variations and the incidence of CRS.^4^

Anatomical changes that can hinder the drainage of the Frontal sinus recess:

a) Anatomical variations of Agger Nasi: It is the most anterior cell of the ethmoid sinus. Endoscopically, it can be described as an elevation of the lateral wall of the nose, prior to the insertion of the vertical portion of the middle turbinate. There are various degrees of pneumatization and location. Therefore, they can narrow the frontal recess posteriorly and/or the nasolacrimal duct laterally.
b) Several cells around the frontal recess can hinder its natural drainage. Below, their descriptions and prevalence’s in the Brazilian population are listed^236^:
  • Agger Nasi cell (95.6%).
  • Supra Agger nasi cell (37.9%): cells anterior to the frontal recess, above the Agger Nasi, without reaching the frontal sinus.
  • Suprafrontal Agger nasi Cell (37.4%): cells anterior to the frontal recess, above the Nasi Agger, reaching the interior of the frontal sinus.
  • Suprabullar cell (77.2%): cells posterior to the frontal recess, above the ethmoid bulla, without reaching the frontal sinus.
  • Suprabullar Frontal Cell (30.1%): cells posterior to the frontal recess, above the ethmoid bulla, reaching the interior of the frontal sinus.
  • Ethmoidal Supraorbital Cell (32.0%): cells posterior/ lateral to the frontal recess, reaching the upper region of the orbit and normally encompassing the anterior ethmoid artery.
  • Septal frontal cell (33.5%): cells medial to the frontal recess, pneumatizing the interfrontal septum.

#### Iatrogenic

a) Recirculation: it can occur when a maxillary antrostomy does not include the main ostium of the maxillary sinus, allowing the secretion usually drained by the main ostium to return to the maxillary sinus through a surgical antrostomy. That is why it is called recirculation.
b) Middle Meatus Synechia: The formation of synechiae between the middle turbinate and the lateral wall of the nose is the most common surgical complication^237^ with prevalence ranging between 4% and 35% and up to 43%.^238^ Instability and lateralization of the middle turbinate are the most significant factors for revision surgeries.^237^, ^238^ Bolger recommended scarification of the medial side of the middle turbinate and adjacent septum mucosa to achieve stabilization of the middle turbinate, maintaining access to the middle meatus.^239^ The septummiddle turbinate suture is effective in preventing lateralization and allowing better penetration of nasal irrigation into the sinus.^240^, ^241^
c) Mucocele: Mucocele occurs when there is total obstruction of a paranasal sinus leading to accumulation of secretion, and ultimately to bone resorption (seen on CT). Postoperative scar stenosis is the main reason for the development of iatrogenic mucoceles. The most common mucocele location is frontoethmoidal. The time interval between previous surgery and diagnosis of mucocele is around 6.25-years, compared with 10-years after external sinonasal trauma. Case series show that 25%‒35% of patients with mucocele have undergone sinus surgery in the past.^242^
d) Dental procedures: oroantral fistulas due to tooth extractions; sinus intrusions of foreign bodies (dental implants and other related reconstructive procedures).

The ABR recommends concomitant surgical correction of the anatomical alterations that compromise the sinonasal drainage pathways or can make postoperative topical therapy difficult, in patients with CRS and indication for surgical treatment.

### Odontogenic sinusitis

Odontogenic Sinusitis (OS) differs from other types of rhinosinusitis regarding its pathophysiology, although the clinical characteristics may be similar.^243^ The cause, as the name suggests, is a dental disorder that compromises the integrity of the maxillary sinus mucosa (Schneiderian membrane). When the floor of the maxillary sinus, is near the dental roots, this anatomical feature can facilitate the spread of infections and other disorders originating from the dental region to the maxillary sinus. The etiology can be infectious (periodontal or periapical lesions), traumatic (tooth fractures), tumoral, or iatrogenic (oroantral fistulas due to dental extractions and sinus intrusions of foreign bodies).^244^ The progressive increasing incidence, mainly caused by the popularization of dental implants and other related reconstructive procedures, have an important impact in terms of public health.^244^, ^245^

The incidence in the general population is not clear, but studies suggest that around 15% of all acute rhinosinusitis have an odontogenic cause.^246^ Among chronic maxillary sinusitis, 25% are attributed to odontogenic disorders,^247^ with a prevalence of 45% to 72% in unilateral diseases.^248^, ^249^

The lack of suspicion of the odontogenic cause leads to the failure of therapeutic success since the basis of OS treatment is precisely the correction of the dental cause.

#### Diagnosis

The combination of nasal and oral symptoms with the imaging study’s findings helps us to differentiate odontogenic sinusitis from non-odontogenic rhinosinusitis.^250^ OS shares most symptoms with other types of RS, such as nasal congestion and rhinorrhea. However, some warning signs suggest the possibility of OS (Table 8).^251^

**Table 8:** Warning signs for odontogenic sinusitis.

Foul nasal odor
Unilateral facial pain
Ipsilateral toothache
Tooth in poor condition
Gum inflammation
Previous dental manipulation

The absence of dental pain does not exclude the diagnosis, since many patients may not present this complaint.^251^ A history of extractions and previous treatments, even if carried out a long time ago, must be questioned.

The examination of the oral cavity must be carried out carefully, evaluating the overall condition of the teeth, absence of dental parts, and disorders such as tooth decay, pain on tooth percussion, fistulas, or gingival findings (hyperemia, edema, abscesses).

On nasal endoscopy, purulent secretion in the middle meatus is a common finding, generally accompanied by edema and mucosal hyperemia. With the chronicity of the inflammatory process, polypoid degeneration may be present. Zhang *et al* demonstrated that, in 88.5% of patients, there are no polyps, in 8.2%, polyps are restricted to the middle meatus and in 3.3% of cases, polyps are extending from the middle meatus to other regions.^252^

On CT findings, opacification of the maxillary sinus, especially if it is unilateral, is suggestive of OS.^253^, ^254^ In the initial stages, there may only be thickening of the sinus floor mucosa (>2 mm) or fluid level.^255^ The lower third of the maxillary sinus, is the point of origin of OS, but the entire sinus must be evaluated since the patency of the Ostiomeatal Complex (OMC) is a valuable information for therapeutic decisions. For example, a patient with periapical disease and ipsilateral isolated maxillary sinusitis, with patent OMC, can be managed initially with antibiotic therapy and dental treatment, with a high chance of success, as sinus drainage and ventilation are reasonably adequate. Another patient, with the same dental finding, but with opacification of the sinus and significant involvement of the OMC, has less chance of success with isolated medical and dental treatment. It is common for patients to be referred by dentists, with only dental imaging exams, with no images that allow a full assessment of the paranasal sinuses. In these cases, CT of the paranasal sinuses should be requested.

The presence of foreign bodies inside the sinus, as well as findings compatible with a fungus ball, are also highly suggestive of OS.

Dental disorders, such as apical periodontitis, are often not diagnosed in tomographic examinations.^253^ It is advisable that patients with unilateral opacification of the maxillary sinus are referred for specialized evaluation (preferably with endodontists) of possible dental problems, even in cases where there are no dental abnormalities detected on the CT.^251^ Cone beam CT has greater sensitivity than conventional CT in identifying odontogenic lesions, being widely used in the diagnosis of OS.^256^

#### Treatment

Considering that the pathophysiology of the disease differs from other types of rhinosinusitis, treatment must also be exclusive, that is, focused on correcting the origin of the disease and possible secondary nasal lesions. Therefore, dental treatment must be associated with sinus treatment.

ENT treatments, which can be surgical or non-surgical, aim to promote the resolution of inflammation, correct obstruction of the natural ostium of the maxillary sinus, and remove irreversible injuries.

#### Medical treatment

The sinonasal medical treatment must be initiated quickly to improve the infection, in conjunction with the earliest possible dental treatment, which generally involves invasive procedures (root canal treatment, periodontitis corrections, fistula closure, etc.). Nonsurgical therapy includes antibiotics (Table 9), nasal corticosteroids, and nasal irrigation to control the symptoms. Many times, the medical treatment, associated with dental procedures, is often sufficient for the patient’s complete recovery.

**Table 9:** Antimicrobial treatment of OS.

Amoxicillin/Clavulanic acid	First option
Clindamycin alone.	Other options in cases of therapeutic failures or allergy to penicillins.
Clindamycin + cefuroxime or ceftriaxone.	
Metronidazole + cefuroxime or amoxicillin with clavulanic acid.	
Fluorquinolones.	

As OS is a disease with a defined oral pathogenesis, there are peculiarities that must be considered when choosing antibiotics. There is generally polymicrobial involvement, predominantly anaerobic bacteria from the upper respiratory tract and oral cavity.^257^ The main microorganisms include Fusobacterium, Prevotella, Porphyromonas, and Peptostreptococcus.^258^ A meta analysis by Chang *et al* demonstrated that Fusobacterium was significantly more prevalent in odontogenic maxillary sinusitis compared to chronic rhinosinusitis, while *Staphylococcus aureus* was more prevalent in CRS. The prevalence of Peptostreptococcus and Prevotella, despite being higher in the OR, was not statistically significant.^259^

Culture of the secretion and antibiogram may be useful, given the microbial diversity of OR. This should be used whenever necessary and available, especially in cases where infectious findings persist after empirical treatment.

Other options in cases of therapeutic failures or allergy to penicillin’s.

#### Surgical treatment

The objective of sinus surgical treatment is to reestablish the drainage and ventilation of the sinus, by opening the maxillary sinus, removing lesions, and inspecting the interior of the sinus, as well as addressing findings outside the jaw, when necessary. Maxillary antrostomy is the chosen technique in most cases. The natural ostium of the maxillary sinus is widened, thus maintaining the natural drainage pathway of the sinus.^260^ The size of the maxillary opening depends on the extent of the patient’s disease, as well as the need for endoscopic inspection of the sinus postoperatively. There are other techniques, such as inferior meatotomy and Caldwell-Luc that can be used in some cases, isolated or associated with other techniques.

There are not enough studies to establish guidelines on the ideal sequence of nasal surgery and dental treatment. Therefore, the conditions of each patient must be considered when making therapeutic decisions. Symptomatic patients, with persistent endoscopic and radiological findings after clinical treatment, should undergo sinonasal endoscopic surgery.^250^ In cases of dental and sinonasal surgery indication, combined (simultaneous) surgery represents a simple approach, with a high percentage of success, low morbidity, low incidence of complications, and rapid recovery, avoiding a series of unsuccessful multiple conservative treatments (antibiotics) and allowing adequate dental reconstructions.^261^

**Table:** 

Odontogenic Sinusitis Remarks
• Dental problems can disturb the integrity of the maxillary sinus mucosa (traumatic, infectious, tumoral or iatrogenic etiology). • Careful evaluation of the clinical dental history, dental treatments and complaints, and detailed inspection of the nasal cavity are essential. The diagnostic hypothesis is generally confirmed with paranasal sinus CT. • The basic treatment is the correction of the dental cause. Medical treatment must be carried out in conjunction with dental treatment, considering a polymicrobial involvement. • Sinus surgical treatment aims to reestablish the sinus drainage and ventilation through maxillary antrostomy with lesion removal.

The ABR recommends that in cases of isolated chronic sinusitis (predominantly maxillary), resistant to usual treatments the dental origin should be investigated. In cases of odontogenic sinusitis, concomitant dental treatment must be discussed with the dentist. If the medical treatment fails, surgical drainage of the affected sinus should be accomplished.

### CRS and immunodeficiencies

The relevance of immunodeficiencies in CRS phenotypes, especially in difficult-to-treat or in frequent recurrent cases, has been demonstrated in several studies. The most frequently described immune defects are those that affect the production of antibodies.^262^, ^263^, ^264^, ^265^, ^266^, ^267^, ^268^, ^269^ Defects in antibody production can be found in up to 50% of cases of treatment resistant CRS.^270^ However, many of the published studies have not used the internationally accepted diagnostic criteria, particularly regarding the diagnosis of the defect of specific antibodies (or antibodies against polysaccharide antigens). The fact is that immunodeficiencies are more common in patients with CRS than in the general population, but given the heterogeneity of the available studies, the real prevalence in patients with CRS, whether pediatric or adult is not clear.^267^

On the other hand, many studies demonstrated a high prevalence of CT changes in patients with CRS and immunodeficiencies, mainly (but not exclusively) in diseases that involve defects in antibody production. CRS can be identified in patients with combined T and B lymphocyte defects, phagocyte defects, complement system defects, and diseases with immune dysregulation.^91^, ^270^, ^271^ On the other hand, the severity of CRS symptoms does not appear to be affected by the presence of an Inborn Error of Immunity (IEI), but rather by the response to its treatment.^267^

Primary immunodeficiencies, currently known as IEI, are a heterogeneous group of genetic diseases whose primary defect is in the immune system, affecting the number and/ or function of different components. The most frequent manifestations are repeated and/or serious infections by common and/or opportunistic microorganisms. In addition to infections, due to immunological dysregulation, manifestations of allergy, autoimmunity, inflammation, lymphoproliferation, and malignancy are also found (Table 10).^83^, ^84^, ^272^

**Table 10:** Classification of primary immunodeficiencies (inborn errors of immunity) according to the International.

Combined immunodeficiencies that affect immunity
cellular and humoral immunities
Combined immunodeficiencies associated with syndromic traits
Predominantly antibody deficiencies
Immune dysregulation diseases
Phagocyte defects (quantitative or functional)
Defects of innate immunity
Autoinflammatory diseases
Complement system deficiencies

In the so-called secondary immunodeficiencies, there are permanent or transient changes in the components of the immune system caused by extrinsic factors, such as metabolic changes (malnutrition, diabetes mellitus); Infectious Agents (HIV); malignant diseases (solid tumors, lymphoma); diseases with protein loss (nephrotic syndrome, enteropathies); medications (corticosteroids, immunosuppressants, rituximab); other genetic diseases (Down syndrome).^86^

The most frequent immune defects worldwide are predominantly antibody production defects or humoral defects. In this group of diseases, the usual presentation is respiratory tract infections caused by common, encapsulated microorganisms. Antibodies are responsible for protecting mucosal surfaces, including the upper and lower airways, acting in defense against extracellular bacteria. Therefore, individuals affected by predominantly antibody deficiencies often develop repeated and/or severe episodes of otitis media, rhinosinusitis, and pneumonia. These patients are also at increased risk of invasive infections due to encapsulated bacteria, including meningitis and osteomyelitis, with or without sepsis.^88^

The most common humoral defect is the selective deficiency of IgA, defined as serum IgA < 7 mg/dL and normal levels of other immunoglobulins in patients aged four years or older. It is usually oligosymptomatic in children and asymptomatic in adults.^89^

The most common symptomatic humoral defect is Common Variable Immunodeficiency characterized by recurrence of upper and lower respiratory infections, bronchiectasis, autoimmunity, granulomatous disease and lymphoproliferation, low serum levels of IgG and IgA and/or IgM, inadequate vaccine response, absence of significative cellular defect, in patients over four years of age.^90^ One study identified that rhinosinusitis was present in 63% of these patients.^273^ Angulo-Pérez *et al* observed a prevalence of CRS in 52% of patients with CVID, 58% had mild severity and in 33% bilateral maxillary sinuses were affected.^274^ Another study identified the presence of chronic mucous thickening in 91% of patients.^275^

Specific Antibody Deficiency (SAD) is a humoral deficiency characterized by normal serum levels of IgG, IgM, and IgA and inadequate response to polysaccharide antigens. SAD has been associated with allergic rhinitis and recurrent infections, including otitis media, rhinosinusitis, and bronchitis. This defect has been described in many papers as the most common immune disease associated with CRS. However, the diagnostic criteria used in many of these publications were not appropriate, disregarding the patient’s previous vaccination status or even valuing the lack of response even before the administration of the nonconjugate vaccine.^89^

Other antibody deficiencies are rare. They include X-linked agammaglobulinemia (very low levels of all immunoglobulins with a B-lymphocyte count <2%) and Union of Immunological Societies (IUIS)^84^ hyper-IgM (normal or greatly increased values of IgM with low levels of other immunoglobulins).^89^

It is important to mention that it is possible to find deficiency in antibody production in different classifications, as part of another group. It is estimated that some defect in antibody production occurs in 75% of IEI. In these cases, upper and lower respiratory infections are usually frequent.

Another group of defects that lead to repeated respiratory infections are defects of the complement system. In these cases, the association with autoimmune diseases such as systemic lupus erythematosus and recurrent meningitis caused by meningococci is common.^89^, ^276^

IEI that affects other components of the immune system can lead to repeated respiratory infections, generally caused by other types of microorganisms. This is what occurs in patients with chronic granulomatous disease (phagocytic defects in the intracellular digestion of microorganisms with greater susceptibility to S. aureus, Gram-negative bacilli, and fungi) or combined defects of T- and B-lymphocytes. In hyper IgE syndrome, there is special susceptibility to *S. aureus* and *Aspergillus spp*. and the presence of rhinosinusitis and chronic otitis media is common.^91^ In these cases, collecting material to identify the involved infectious agent is essential.

Before performing any surgical approach to CRS resistant to clinical treatment, it is interesting to investigate immunological defects.^270^

The investigation must begin with a careful clinical history: associated infections, frequency, location, severity, need for hospitalization, identification of infectious agent; associated manifestations, such as autoimmunity, lymphoproliferation, allergy, bronchiectasis; use of medications; vaccination history; family history. A complete physical examination can identify phenotypes characteristic of some IEI, such as oculocutaneous telangiectasia, partial albinism, absence of palatine tonsils, and cerebellar-type ataxia.^92^, ^265^, ^276^

Laboratory investigation of antibody production defects is carried out with serum measurement of immunoglobulins (A, M, G, and E), B-lymphocyte count (CD19), and assessment of response to vaccines (tetanus, diphtheria, measles, rubella, mumps). Immunoglobulin values must be analyzed according to references by age group. The diagnosis of specific antibody defect is made by comparing IgG levels for pneumococcal serotypes absent in the conjugate vaccines, before and after the administrations of the 23-valent pneumococcal vaccine (not conjugated to protein) and must always consider the previous vaccination status regarding pneumococcal vaccines.^93^ Research for HIV infection is essential, in any age group.^91^

Low values of IgG subclasses, although valued in some publications, must be interpreted with caution, as in many cases they lack clinical significance, particularly low levels of IgG4. Functional assessment through vaccine response is essential in these cases.^270^, ^277^

The measurement of secretory IgA is not part of the investigation of IEI since its absence lacks clinical significance.

Other types of immunological defects should be investigated when infections by Gram-negative bacteria, fungi, or opportunistic microorganisms occur. The investigation must be directed according to the type of suspected or identified infectious agent, as well as the associated non-infectious manifestations (see item 2.2.3).^278^

The treatment of choice for antibody defects is immunoglobulin replacement, intravenously or subcutaneously. It is indicated whenever immunoglobulin levels are very low or there is no good response to vaccines. There is no indication of replacement in selective IgA deficiency.

Antibiotics are indicated for active infections and for the prophylaxis of recurrent infections, especially in defects for which there is no indication for immunoglobulin replacement therapy. As prophylaxis, sulfamethoxazoletrimethoprim, azithromycin, or amoxicillin are commonly used.^270^

Surgical treatment of CRS is not contraindicated in patients with immunodeficiency, and it is possible that the surgery will bring benefits, especially if the treatment of the underlying disease is difficult to control.^278^, ^279^

Early diagnosis and treatment are essential to prevent morbidity and mortality related to IEI, as well as those related to secondary immunodeficiencies (Table 11).

**Table:** 

Immunodeficiencies
• Immunodeficiencies can be primary, called Inborn Error of Immunity (IEI), or secondary to genetic, infectious, or malignant diseases. • The most common symptomatic IEI is common variable immunodeficiency, that presents with recurrent respiratory tract infections. • Treatment for humoral defects consists of replacement of immunoglobulins, antibiotics for active infections and /or prophylaxis.

**Table 11:** Warning signs for suspected IEI.

CRS poorly responsive to medical and surgical treatment
Recurrence of CRS after initial response to treatment
Familial history of an IEI or consanguinity
Presence of other sites repeated infections
Unusual/opportunistic microorganisms’ infections
Severe and/or prolonged infections that require hospitalization for IV antibiotic therapy
Association with bronchiectasis
Other non-infectious manifestations related to IEI: syndromic features, growth deficit, autoimmunity

The ABR recommends that, in cases of difficult-to treat CRSsNP, immunodeficiencies should be investigated, through anamnesis directed to the warning signs of IEI, and appropriate laboratory investigation.

### Granulomatosis with Polyangiitis (GPA)

Granulomatosis with Polyangiitis (GPA), previously named Wegener’s Granulomatosis, is a necrotizing vasculitis that predominantly affects small and medium-sized vessels. It is characterized by granulomatous inflammation and fibrinoid necrosis, and the formation of necrotizing granulomas. The most common clinical manifestations in otorhinolaryngology are chronic rhinosinusitis, otitis media, and inflammation of the upper and lower respiratory tract.^280^, ^281^, ^282^

Its annual incidence is estimated at approximately 10–20 cases per million individuals. Particularly in North America and Western Europe populations, GPA is a rare autoimmune disease.^283^ The disease affects predominantly middle-aged male adults. Studies indicate that GPA is more common in individuals of European origin, with a lower incidence in other ethnical groups. The geographic distribution of GPA shows higher prevalence in countries with temperate climates, possibly due to environmental, genetic, and access to medical care factors. In Brazil, specific data on the incidence and epidemiology of GPA are scarce. The lack of detailed national registries and variability in diagnostic methods may contribute to disease underreporting.^281^, ^284^

Regarding pathophysiological characteristics, GPA is characterized by the presence of Antineutrophil Cytoplasmic Antibodies (ANCA), typically directed against Proteinase 3 (PR3), which play a central role in the pathophysiology of the disease. Neutrophil activation and adhesion to the vascular endothelium result in inflammation and tissue damage, characteristic of the necrotizing vasculitis observed in this disease. This process is mediated by the interaction of ANCAs with their target antigens, leading to the formation and deposition of immune complexes, which perpetuate endothelial inflammation and vascular injuries.^281^, ^285^ Affected tissues, show granulomatous inflammation along with necrotizing granulomas, predominantly in the upper and lower respiratory tract, as well as in the kidneys, which leads to segmental glomerulonephritis.^286^, ^287^, ^288^

Recurrent nasal and paranasal sinus manifestations are common. It is estimated that 70%–100% of patients exhibit otorhinolaryngological symptoms at the moment of diagnosis, such as crusted rhinorrhea, rhinosinusitis, chronic otitis media, and facial cartilage deformities, including saddle nose, perforation of the nasal septum and palate.^283^ The initial presentation may include nasal obstruction, purulent rhinorrhea and hyposmia or anosmia. Nasal obstruction and nasal crusts are the earliest signs of GPA.^280^, ^281^ Chronic and destructive inflammation in the upper airways can result in more serious complications, such as the formation of granulomas and scar tissue that affect nasal and paranasal sinus structures and function. In addition to nasal and sinus manifestations, GPA can affect other otorhinolaryngological loci. Chronic otitis media is reported in up to 22.5% of cases, resulting from Eustachian tube dysfunction and granulomatous lesions in the upper airways.^280^, ^283^ Patients may also present with hearing loss, conductive or sensorineural, developing over the course of weeks or suddenly. Other otorhinolaryngological complications include facial nerve paralysis that although rare, can occur due to vasculitis of the small vessels that supply the nerve. Additionally, subglottic stenosis with symptoms such as stridor and hoarseness, affects approximately 16% of patients with GPA.^280^, ^285^

In addition to the otolaryngological symptoms, GPA presents a variety of non-ENT manifestations that encompass multiple body systems. Pulmonary involvement occurs in 50%–90% of patients, characterized by alveolar hemorrhage and parenchymal nodules.^280^, ^283^ Renal inflammation is frequently observed, presenting as segmental and focal necrotizing glomerulonephritis, with extra capillary proliferation, negatively impacting the prognosis of the disease.^280^ Peripheral nervous system involvement affects about one-third of patients, manifesting primarily as mononeuritis multiplex or sensorimotor neuropathy.^281^ Skin involvement, such as rash, and systemic symptoms such as fever, weight loss, asthenia, and arthralgias are common. GPA can also cause cranial neuropathies, mainly of the optic and olfactory nerves as well as affecting ocular motility.^289^, ^290^

The diagnosis of GPA is based on a combination of clinical, laboratory, and imaging criteria, as shown in Table 12.

**Table 12:** Diagnostic criteria for Granulomatosis with Polyangiitis (GPA) according to the American College of Rheumatology.^281^, ^286^

Nasal or oral inflammation
Abnormal findings on Chest X-Ray
Urinary sediment with microhematuria or blood casts
Granulomatous Inflammation shown in biopsy

The presence of ANCA autoantibodies, specifically C-ANCA, and antiproteinase-3, is a common finding and can significantly aid the diagnosis since the test has a high rate of sensitivity and specificity. Additional important laboratory tests include complete blood count, Erythrocyte Sedimentation Rate (ESR), C-Reactive Protein (PCR), and urinalysis, which may indicate glomerulonephritis. Imaging tests also play a key role; CT and chest X-Rays often reveal fixed infiltrates, cavitations in the lungs, and other disease features. Histopathology is critical to the diagnosis, particularly for identifying necrotizing vasculitis of small and medium vessels, the presence of granulomas, and multinucleated giant cells.^281^, ^283^

In routine otorhinolaryngology, the diagnosis involves a comprehensive evaluation that includes a detailed medical history and, a complete physical examination of the head and neck, with particular attention to nasal and sinus symptoms.^281^ Nasal endoscopy is often used to inspect the nasal cavity and identify typical features such as crusting, ulcers, bloody rhinorrhea, septal defects or perforations and effusions in the middle ear.^283^

GPA treatment involves specific approaches to induce remission of the disease and long-term maintenance, including immunosuppressants and corticosteroids. In the induction phase, systemic corticosteroids combined with immunosuppressive agents such as cyclophosphamide or rituximab are recommended.^282^ Cyclophosphamide is often administered in pulsed doses of 15 mg/kg every two weeks, followed by adjusted dosages according to the patient’s response.^289^ Rituximab, in turn, can be administered in doses of 375 mg/m^2^ weekly, for a total of four doses.^290^ Patients with less severe disease can be treated with methotrexate as induction therapy.^281^

After inducing the remission, maintenance therapy is essential to prevent relapse, usually combining low-dose corticosteroids with azathioprine or methotrexate.^280^, ^281^ Maintenance treatment is often continued for at least 24-months and may include TMP-SMX prophylaxis to prevent infection by Pneumocystis jirovecii.^280^, ^281^, ^292^ Recent studies indicated that rituximab could provide superior remission benefits compared to azathioprine, showing a lower risk of relapses.^281^

The role of surgery in GPA is limited. Surgical intervention is generally reserved when medical interventions are not sufficient and when necessary, should always be performed when the disease is in remission, due to reduced tissue quality and healing capacity while the disease is active.^281^, ^286^, ^293^ Surgical indications include correction of external nasal deformities for aesthetic and functional purposes. Subglottic stenosis may also require surgical intervention in severe and refractory cases, although the evidence is still limited.^281^, ^282^

Another scenario when surgery may be needed would be in cases with chronic otitis media, for placement of ventilation tubes. However, it is important to highlight that, even with surgical treatment, the need for multiple procedures to achieve satisfactory symptomatic control is common.^285^

Careful monitoring of patients and collaboration work with a multidisciplinary team are fundamental to the effective management of GPA. Depending on the organic manifestations and severity of the disease, professionals such as rheumatologists, nephrologists, pulmonologists, ophthalmologists, and neurologists may be involved in patient care.^281^, ^290^, ^291^

**Table:** 

Granulomatosis with polyangeitis (GPA)
• Granulomatosis with Polyangiitis (GPA) is an autoimmune vasculitis of small and medium vessels, which presents with necrotizing granulomas in the upper and lower airways and kidneys. • Clinical picture: rhinosinusitis associated with purulent secretion and formation of crusts, facial cartilage deformities and chronic otitis media. • Diagnosis is based on clinical, laboratory and imaging criteria. • Treatment is based on the use of systemic corticosteroids and immunosuppressive agents to induce remission and afterwards, maintenance must be carried out for at least 24-months with low-dose corticosteroids, methotrexate and azathioprine. • The role of surgery is limited and must be performed in the remission period.

The ABR recommends that in cases of CRSsNP with suspected GPA (purulent secretion, crusting, cartilaginous deformities, and chronic otitis media), a biopsy of the nasal lesions and a multidisciplinary evaluation should be performed.

### Primary ciliary dyskinesia

Primary Ciliary Dyskinesia (PCD) is an autosomal recessive disease in which changes in the ultrastructure of the hair cells result in dysfunction of the ciliary motility.^294^ In European countries, the estimated prevalence is 1 in 10,000–20,000 live births,^295^ however, it is known that this number is underestimated.^296^ In Brazil, there are no studies on the prevalence of PCD.

In this disease, pathogenic genetic variants will determine changes in the proteins that are present in the ciliary ultrastructure of the ciliated cells, therefore compromising ciliary beating and its functions: mucociliary clearance, fertility, and embryogenesis.^296^, ^297^

In the upper and lower airways, cilia are responsible for mucociliary clearance, which plays an important role in the body’s innate defense.^298^, ^299^ The effectiveness of mucociliary clearance is related to the characteristics of the mucus, the structure of the cilium, and the function: synchronicity and frequency of ciliary beat.^300^, ^301^ Thus, patients with PCD will present secondary diffuse CRS of mechanical endotype.^4^

These mucociliary clearance action changes of the sinonasal mucosa will lead to mucus accumulation, disturbance of the airway microbiota, infections, and structural alterations with consequent functional disorders, resulting in severe clinical repercussions.^294^, ^302^

Currently, approximately 45 genes related to PCD have already been identified, but in many cases, the genetic variants are not known.^303^ This large genomic variety results in a significant phenotypic multiplicity, which makes PCD a very heterogeneous disease in terms of clinical features and evolution.^297^, ^302^ Or, in other words, to diagnose patients with ciliary dyskinesia it is necessary to think beyond the classic Kartagener syndrome, described in patients with bronchiectasis, situs inversus totals, and chronic rhinosinusitis. There are numerous phenotypes of ciliary dyskinesia in which situs inversus, infertility, and chronic otitis media may or may not be present. Furthermore, ideally, the diagnosis of PCD in children should be made before the occurrence of structural lung changes such as bronchiectasis.^294^, ^296^, ^299^

#### Clinical presentation

PCD is a congenital disease that presents phenotypic diversity, as well as great variability in symptoms as the disease progresses. In the neonatal period, PCD may be suspected in patients with a positive familial history. Furthermore, laterality defects such as dextrocardia and situs inversus, congenital cardiac abnormalities, and hydrocephalus may be present on ultrasound. In the neonatal period, respiratory distress and atelectasis can be observed in full-term patients, without risk factors, necessitating prolonged oxygen therapy. Patients commonly present with persistent rhinorrhea and chronic rhinitis since the first weeks of life.^294^, ^304^, ^305^ In addition to chronic rhinitis, chronic productive cough, recurrent serous or acute otitis media, and consequently conductive hearing loss may occur.

Furthermore, toddlers can have recurrent pneumonia, and bronchiectasis may start to appear in this period.

They are present in 100% of adults. In addition to bronchiectasis, CRS can begin in childhood and persist into adulthood. CRSwNP is more common in adolescents and adult patients. In adults, infertility can also be identified in both sexes.^294^, ^304^, ^305^

CRS is present in 70% of adult patients with PCD and, they also have a smaller volume of the maxillary, sphenoid, and frontal sinuses, due to a decrease in pneumatization.^306^ The inflammatory cytokine profile in PCD patients with CRS has not been specifically studied, however, the mucus resembles cystic fibrosis.^307^ CRSwNP is less common in PCD than in cystic fibrosis. There is evidence that patients colonized by *P. aeruginosa* present the same strain in the paranasal sinuses and lungs, corroborating the hypothesis that early eradication of paranasal sinus colonization could reduce lower airway infections.^307^, ^308^

Patients with PCD have greater olfactory impairment compared to patients with primary CRS, especially those with severe damage to the ciliary ultrastructure. The main hypothesis for this finding is related to impaired ciliary mobility and reduced primary sensory function.^309^, ^310^

#### Diagnosis

The diagnosis of ciliary dyskinesia is challenging, as there is no gold standard test, due to the phenotypic and genotypic variability of the disease and the cost and complexity of diagnostic tools.^294^, ^295^, ^299^ In Brazil, few centers are equipped with the necessary tools for the diagnosis.^310^ Even the European and the American consensus differ in terms of PCD diagnostic algorithm. Initially, it is necessary to exclude cystic fibrosis in these patients, as CF is a disease with similar symptoms and higher prevalence. Therefore, sodium/chlorine measurement in sweat or CFTR genetic evaluation should be requested.^311^

There are some validated questionnaires for suspected cases of PCD as screening tools.^312^, ^313^ Nasally exhaled nitric oxide is one of the diagnostic tools and is generally decreased in patients with PCD. However, it is difficult to perform the test in patients under 5-years of age and false positive and negative results may occur.^100^, ^312^ High-speed video microscopy evaluates the function of ciliary cell beats after nasal or lung epithelium biopsy, however, it may be normal in some PCD phenotypes. Transmission electron microscopy analyses defects in the ciliary ultrastructure that are compatible with PCD, however, around 30% of patients may have normal ciliary ultrastructure and altered ciliary beat function.^294^, ^302^, ^311^ The evaluation of pathogenic genetic variants has shown increasing diagnostic value, especially after the whole exome analyses, however, the knowledge of all genetic variants that cause PCD is still limited, and just like other diagnostic tools, should not be evaluated alone.^297^

#### Treatment

Due to the difficulty in diagnosing PCD, besides the rarity of the disease, there is no evidence-based therapeutic intervention for this disease. However, there are some international consensuses based on the opinion of experts.^294^, ^302^

The recommendation is for personalized evaluation of each patient and individualized treatment, considering some peculiarities of the disease such as mucociliary clearance dysfunction and colonization by *Pseudomonas aureginosa* and *Staphylococcus*, which can be synchronous in the paranasal and pulmonary sinuses.^308^, ^314^, ^315^ As these peculiarities are common between cystic fibrosis and PCD, treatments for PCD are in general based on evidence related to cystic fibrosis.^307^

Medical management may include intranasal and oral corticosteroids, particularly in patients with CRSwNP and long-term macrolide therapy.^315^ The use of isotonic and hypertonic nasal saline solutions is recommended to improve nasal mucociliary clearance.^315^, ^316^ Recombinant human Deoxyribonuclease (rhDNase), hypertonic saline, or mannitol have questionable efficacy in PCD. Prophylactic antibiotic therapy is not indicated, however, in cases presenting with respiratory symptoms and exacerbation of rhinosinusitis, long-term antibiotics should be used, preferably guided by culture.^314^, ^315^, ^317^

Alanin *et al* evaluated ESS associated with postoperative treatment with antibiotics, nasal lavage with saline solution, and intranasal corticosteroids in 16 patients with PCD.^316^ Despite the small number of studied patients, ESS significantly improved SNOT22, especially in patients with rhinosinusitis caused by *P. aeruginosa* or intermittent pulmonary colonization, ESS showed potential benefit regarding pulmonary colonization, similar to what occurs in CF.^307^, ^316^ However, there are no controlled studies comparing surgery with medical treatment alone. Thus, the recommendation for surgical management should be made considering the patient’s symptoms, failure in clinical treatment, sinus and pulmonary colonization, and the severity of the disease.^307^, ^316^, ^317^

**Table:** 

Primary Ciliary Diskynesia (PCD)
• Primary Ciliary Dyskinesia (PCD) is a genetic condition in which ciliary cell beating is congenitally altered. • PCD has great variability in clinical presentation and may present with rhinosinusitis, chronic rhinitis, chronic otitis media, bronchiectasis, recurrent respiratory infections, dextrocardia and situs inversus and, fertility disorders. • The diagnosis is made in patients with a suggestive clinical history associated with more than one complementary test (nitric oxide, electron microscopy, assessment of ciliary beat and genetic alterations). • Treatment includes intranasal corticosteroids, nasal lavage with saline solution, antibiotics, and sinonasal surgery.

The ABR recommends that, in cases of CRS and bronchiectasis, recurrent respiratory infections, chronic otitis media, dextrocardia, situs inversus, and/or fertility disorders, the suspicion of PCD should be considered.

### Chronic rhinosinusitis without nasal polyps NON-TYPE 2

This item refers to patients with CRSsNP in whom the phenotypes described earlier in this document have not been identified. For most, the etiology is uncertain, although several environmental and host genetic factors have been imputed. From the perspective of pathogenesis, these environmental and host factors interact over time to trigger one or more immunological pathways (endotypes) of chronic tissue inflammation that lead to clinical presentation (phenotype).^4^, ^318^

From the point of view of etiopathogenesis, the predominant hypothesis, not yet proven, is that these various inflammatory mechanisms are driven by dysfunctional interactions on the mucosal surface between the host and environmental stressors. From a host perspective, genetic and epigenetic variation of the mucosal immune system is thought to play a key role in CRS, but multiple genes are likely to be involved, and to date, very few have been associated with a broad-sized effect. Major environmental agents also remain largely varied, but cigarette smoke, fungi, viruses, bacteria, pollutants, and allergens are involved. The most discussed microbial agent is Staphylococcus aureus, but some evidence also implies dysbiosis of the microbiota, not only associated with a single pathological agent.^4^, ^319^ A wide variety of exogenous agents are inhaled through the nose and interact with the sinonasal mucosa. It is a process that begins at birth with rapid colonization by viruses, bacteria, and fungi. In healthy individuals, the mucosa serves as a relative barrier that limits and regulates environmental interaction with the host’s immune system, a process that is likely beneficial to the host in several ways, including developing tolerance, generating important metabolites, and competitive inhibition against pathogens.

Non-eosinophilic CRS occurs in middle-aged patients, more common in obese females, without a relevant history of corticosteroid therapy. The presence of hyposmia is rare and even when present, the response to oral corticosteroids is poor. Likewise, it is not uncommon that oral corticosteroids do not significantly reduce overall symptoms.^318^

Nasal endoscopy may find swollen mucosa but without eosinophilic mucin. However, the presence of thick rhinorrhea is common, sometimes frankly purulent. CT shows pansinusal opacification, often indistinguishable from patients with eosinophilic CRS (eCRS).^318^ Histopathologically, inflammation is predominantly neutrophil-mediated, so that tissue eosinophilia is negligible, which leads to the marked presence of non-type 2 proinflammatory cytokines (IL-1β, 6, and 8). Allergy tests are negative and serum IgE is normal.^318^

Therapeutic options include intranasal corticosteroids and nasal saline irrigation. The effect of topical corticosteroids is lesser than in patients with eCRS. Some studies suggest using low doses of macrolides for long periods, due to their modulating effect on the inflammatory activity of neutrophils, as a therapeutic alternative.^4^, ^318^, ^320^ Clarithromycin, 250 mg daily for 3-months, with reassessment of the response after this period, is also indicated, attempting to the importance of electrocardiographic follow-up due to the risk of elongated QT interval.^318^

Currently, another option would be using 5% xylitol in high-volume nasal washing. Xylitol is a sugar alcohol, with five carbons, which has a beneficial effect on the sinonasal mucosa since it can reduce nasal symptoms even in patients without previous surgery.^321^ There are indications that xylitol can inhibit bacterial biofilm, acting against bacterial growth and adhesion. Studies suggest that xylitol enhances the recovery of the mucosa after a surgical procedure, improving ciliary beating, mucociliary clearance, and removal of crusts.^322^ There are formulations on the market containing xylitol in spray or sachet for high-volume washing, the latter being more recommended for CRS, due to penetration into the sinonasal cavities, especially after surgery. The recommended dose is 5% xylitol, 12 g diluted in 240 mL of filtered or boiled water. Benefits have been observed in cases of CRS without nasal polyps, however, studies are necessary to confirm it.

Oral corticosteroids are generally not recommended for the treatment of CRSNsNP unless there is a strong suspicion that the disease involves predominantly type 2 inflammation.

Patients with CRSsPN who do not respond to medical therapy are good candidates for ESS to relieve sinonasal obstruction.

**Table:** 

CRSsNP non type 2
Frequent complains nasal secretion/facial pain
Asthma is less frequent. When present, usually neutrophilic
Nasal endoscopy: purulent secretion, mucosal edema
Laboratorial exams: normal level IgE, no eosinophilia
Treatment: antibiotics, long-term macrolides, nasal irrigation, topic xylitol, intranasal corticosteroids, ESS.

## Chronic rhinosinusitis with nasal polyps

### Cystic Fibrosis

Cystic Fibrosis (CF) is an autosomal recessive genetic disease, which courses with pathogenic variants of the CFTR (Cystic Fibrosis Transmembrane Regulator) gene, located on the long arm of chromosome 7. The mutation in the transmembrane conductance of CFTR leads to defective chloride channels, resulting in a marked increase in the viscosity of secretions.^323^, ^324^, ^325^

The most common mutation of CFTR gene is the F508del deletion, which is observed in approximately half of Caucasian patients in Brazil. About two thousand variants have been described, however, only 15% of them cause CF.^326^ The CFTR gene encodes a protein of the same name, whose function is the chloride transport channel. Depending on the type of genetic variant, the CFTR protein may be absent, deficient, or dysfunctional. Depending on the alteration, patients may present with milder or more severe clinical phenotypes.^324^, ^327^ Genotype also seems to influence sinus disease severity, and despite few studies, there is a probable association between severe extrapulmonary disease and a higher prevalence of recurrent CRSwNP.^328^ Neonatal screening includes investigation for CF that is based on immunoreactive trypsinogen levels. If the newborn has two positive dosages, confirmation is accomplished with a sweat test. The sweat test can confirm the diagnosis and may be associated with the search for genetic mutations related to cystic fibrosis and CFTR protein function tests.^329^, ^330^

#### Clinical picture of cystic fibrosis

The CFTR protein is in the apical part of the cells of several organs and functions as an epithelial channel for chlorine. It is a multisystem disease that may present structural and functional changes; it is frequent in the pancreas, liver, muscles, bones, urinary and reproductive tract. In the respiratory and digestive tract, however, the dysfunction of this protein results in the most serious consequences, such as chronic respiratory infection, pancreatic insufficiency, and malnutrition. In the upper and lower airways, the alteration in the chloride channel results in the thickening of the mucus and consequently impairment of mucociliary clearance, bacterial colonization, and chronic inflammation. Enzymes produced by bacterial colonization, especially Pseudomonas aeruginosa, lead to cilia disturbs such as impairment of ciliary beating and ciliary rupture, which further aggravates the mucociliary clearance process.^331^, ^332^

In the sinonasal tract, chronic rhinosinusitis is the main manifestation, present in more than 90% of cases. The mechanical obstruction caused by the impairment of mucociliary clearance leads to blockage of the sinonasal drainage ostia, and patients present diffuse involvement of the paranasal sinuses, especially the maxillary and ethmoidal sinuses.^324^

In about two-thirds of CF patients, nasal polyps may be present, and unlike other phenotypes of chronic rhinosinusitis, polyps are common in children and adolescents. Thus, in pediatric patients with chronic rhinosinusitis with nasal polyps, it is recommended to search for lower airway and pancreatic changes to exclude CF.^324^, ^333^

Sinus bacterial infections and bacterial colonization are themes of great attention in the literature. There is some evidence that bacteria with the same genotype colonize the paranasal sinuses and resemble that of induced sputum.^334^

Although almost all patients with cystic fibrosis present sinonasal alterations on imaging exams, only 10%–15% will present symptoms. Thus, these radiological tests should be interpreted with caution, especially regarding surgical recommendations. One should also consider the real need to perform multiple imaging exams, to avoid unnecessary radiation exposure. Despite the lack of robust scientific evidence that the treatment of the upper airways implies an improvement of the pulmonary condition, some authors believe that the decrease in colonization and inflammation of the upper airway could reduce bronchial reactivity, inflammatory burden, and colonization of the lower airway.

Patients with CF may present with typical findings of chronic rhinosinusitis such as thick nasal discharge, middle meatus edema, and nasal polyps. Other common findings in CF on CT include bulging of the medial wall of the maxillary sinuses, with remodeling of the bone walls known as pseudomucocele. The main hypotheses for these findings are alterations of the uncinate process, osteitis of the lateral wall, or pressure exerted by the thick mucus, peculiar to CF, against the walls of the maxillary sinuses or ethmoidal cells.^325^, ^331^, ^335^, ^336^

#### Treatment of cystic fibrosis

The treatment of the patient with CF should consider the patient’s upper airway symptomatology. CT of the paranasal sinuses will show alterations in most cases and is not a good parameter for treatment indication. Specific systemic antimicrobials, aiming to eradicate *P. aeruginosa or S. aureus* should be recommended in conjunction with the lower airway approach, and a multidisciplinary approach is essential for these patients.

Nasal lavage with physiological or hypertonic saline solutions is commonly recommended in patients with nasal discharge, despite the lack of robust clinical trials proving the efficacy of this intervention. Topical corticosteroids should be prescribed in patients with associated allergic rhinitis, but systemic corticosteroids should be avoided in patients with CF, due to the lack of benefits evidence.^337^

Currently, there are drugs that act at the molecular level, correcting the defects of the CFTR protein, such as Ivacaftor, Lumacaftor (Orkambi), Tezacaftor (Symkevi or Symdeko), or Elexacaftor (associated with Tezacaftor as Trikafta).^336^ These medications act directly on the disease, significantly improving the quality of life and life expectancy of CF patients. Although there are not enough studies on the performance of these medications in the upper airways, they seem very promising.

Endoscopic sinonasal surgery should be recommended in patients with symptoms refractory to appropriate medical treatment, regardless of age group. Surgery is generally well tolerated, but the risks must be considered, especially in patients with deteriorated lung function.

**Table:** 

Cystic fibrosis
• Cystic fibrosis (CF) is a disease caused by pathogenic variants of the CFTR gene, resulting in increased thickness of secretions in various tissues of the body. • Impairment of sinonasal mucociliary clearance, leading to bacterial colonization and chronic inflammation, and chronic rhinosinusitis with polyposis. • Treatment involves physiological or hypertonic saline solutions, antibiotics and medications that act at a molecular level, correcting defects of the CFTR protein. • There is no consensus on surgery recommendation, and it may be indicated in symptomatic patients despite adequate clinical treatment

### CCAD ‒ Central Compartment Atopic Disease

CCAD is a variant of the recently described chronic rhinosinusitis type 2 inflammation and is significantly associated with IgE-mediated allergy. In 2014, White *et al* for the first time presented polypoid and edematous changes in the middle turbinate in 25 patients who tested positive for inhalant allergens. The proposed etiology was that the appearance of the anterior part of the middle turbinate, being exposed to inhalant allergens through the nasal flow, would lead to edematous and polypoid alterations in that site.^336^, ^338^

Brunner *et al* also observed a higher association of isolated middle turbinate alterations in allergen-sensitized patients than in those with diffuse polyposis.^339^ Del Gaudio *et al* revealed that other central structures such as the posterosuperior septum and superior turbinates are also involved in this variant of chronic rhinosinusitis.^340^ Hamizan *et al* described that a central pattern of mucosal alteration is highly associated with allergy.^341^

CCAD disease is a phenotype of CRS described in highly allergic patients, with inflammatory changes (obstructive edema, polypoid mucosa, and thickening) affecting the central portion of the sinonasal cavity, sparing the lateral walls, as seen on endoscopy and CT.^342^

#### Comparison with other respiratory allergic diseases

CCAD is a nasal inflammatory process that results from an allergy to inhalants. It provides us with a definitive link between CRSwNP and allergy.^343^

Patients with CCAD have a higher prevalence of atopy, and a higher mean serum IgE than other subtypes of CRS.^344^ Compared with chronic eosinophilic rhinosinusitis, patients with CCAD are younger, and the number of circulating eosinophils and eotaxin is lower.^345^ Regarding type 2 cytokines, such as IL-5 and IL-13, CCAD has lower rates than AERD and AFRS, and a lower number of tissue eosinophils.^346^

Patients with CCAD have a high incidence of allergy and a low incidence of asthma, like AFRS, and lower than other types of CRSwNP. According to Marcus *et al*, allergic rhinitis was detected in 100% of patients with AFRS, 97.6% in CCAD, 82.6% in AERD, and 56.1% in CRSwNP. The incidence of asthma was 17.1% in ACHD, 19% in the AFRS, 30.8% in the CRSwNP, and 100% in the AERD.^347^, ^135^

#### Nasal endoscopy in atopic central compartment disease

The most common endoscopic features of ACCD are edema or polypoid mucosa of the middle turbinate, superior turbinate, and/or posterosuperior septum. According to Lau *et al*, endoscopy is a better predictor of inhalant allergy than CT in patients with CRS and CCAD. Nasal endoscopy findings should indicate to the otorhinolaryngologist the search for a correlation between the patient’s clinical history of allergic rhinitis and CCAD, then, diagnostic confirmation with allergy tests is recommended.^344^

#### Computed tomography of the paranasal sinuses in Atopic Central Compartment Disease

In allergic patients, according to a 2017 study by Brook who evaluated 216 cases, patients with an in vitro specific test for allergens did not have greater radiographic signs of sinus inflammation.^347^ In another study of allergic patients and CT images, in the group with allergic rhinitis and asthma, 58% had sinus abnormalities on CT, versus 74% in asthma-only patients, 67% in rhinitis alone, and 20% in the control group. A central radiological pattern of mucosal disease may be associated with sensitization to inhalant allergens.

This group may represent the subgroup of patients with atopic central compartment disease.^341^ The prevalence of allergy in chronic rhinosinusitis can vary according to the phenotype, CCAD and AFRS have the strongest association.^4^

#### Medical treatment

As already known, several endotypes are responsible for the phenotype of nasal polyps, and the prevalence of asthma and allergy varies widely among these endotypes. Evidence is limited on how allergy treatment affects the management of different subtypes of nasal polyps. Usually, a treatment that considers the upper and lower airway results in better outcomes for these patients.^342^

Treatment includes topical intranasal corticosteroids, nasal washes with saline solutions, and functional endoscopic surgery. In patients with allergic rhinitis, intranasal topical corticosteroids and topical or oral antihistamines are recommended; and in patients with asthma, inhaled corticosteroids associated with bronchodilators.^348^, ^138^

Since CCAD is an allergic disease with sensitization to inhalant agents, the option of allergen-specific immunotherapy is also listed as an option for treatment, but more studies are still needed.^343^

When symptoms cannot be controlled with the usual treatments and appropriate surgery, biologic therapy may be an option for these patients, especially omalizumab.^348^

#### Surgical treatment

It is not yet clear why patients with allergic rhinitis develop CCAD, but we do know that patients with isolated CCAD respond well to conservative endonasal surgery in the involved areas, with lower incidence of polyp recurrence and revision surgery than the other subtypes of CRSwNP.^343^

**Table:** 

Central compartment atopic disease (CCAD)
• CCAD is a condition in which inflammatory changes such as edema and polyps affect the central portion of the sinonasal cavity, in allergic individuals. • CCAD occurs in young patients, has a higher prevalence of atopy, increased serum IgE and eosinophilia. • Nasal endoscopy and sinus CT show a central pattern of inflammation in the middle and superior turbinates, ethmoid cells and posterosuperior septum. • Medical treatment includes intranasal topical corticosteroids, nasal saline irrigation, and allergen specific immunotherapy. • This phenotype responds well to surgical treatment.

The ABR recommends that in CCAD cases, medical treatment should include allergy control, and in refractory patients, surgical treatment must be considered.

### Aspirin exacerbated respiratory disease (AERD) or Nonsteroidal Anti-Inflammatory Drugs (NSAIDs)

Aspirin Exacerbated Respiratory Disease (AERD) or (Nonsteroidal anti-inflammatory drug (NSAID) is a syndrome, defined as a classic triad: moderate to severe asthma, chronic rhinosinusitis with nasal polyps, and hypersensitivity to aspirin and other COX-1 inhibitor NSAIDs, in which a type 2 immune response predominates, with marked both, tissue and peripheral blood eosinophilia.^186^

The prevalence of AERD is unknown. In patients with asthma, 5.5%–12.4% have AERD, 21% of whom are diagnosed only after oral challenge; in patients with CRSwNP, about 10%–20% have AERD.^175^, ^186^, ^191^

According to the main guidelines, AERD is one of the worst prognostic phenotypes of CRSwNP, in which the patient requires treatment with multiple courses of oral corticosteroids, and several surgeries, often without the expected success. The disease usually courses with a high rate of recurrences, and great difficulty in controlling the symptoms. The negative impact on the quality of life of these patients is very significant and consequently brings many financial losses.^349^

#### Clinical diagnosis of AERD

Patients with AERD usually present the first symptoms in adulthood, usually after the age of 30. They report several episodes of acute sinus infections, and worsening of symptoms caused by alcohol or food. The main complaints are nasal obstruction or congestion, nasal discharge (anterior and/or posterior), cacosmia, chronic cough, facial pressure or pain, and progressive loss of smell. Patients respond very well to oral corticosteroids, even if only for a short period of time.^191^, ^349^ All patients with CRSwPN, type 2 inflammation, should be questioned about asthma and hypersensitivity to aspirin and NSAIDs that are COX-1 inhibitors.^186^

Asthma, also late-onset, usually moderate to severe, can appear before, during, or even after nasal complaints. These patients have a high prevalence of atopy. The risk of uncontrolled asthma in patients with AERD doubles; severe asthma or asthma exacerbations increase by 60%; emergency room visits increase by 80% and hospitalization due to asthma, 40%.^142^ It is always very important when taking the medical history, to ask the patient if they have or ever had an asthma attack or any type of reaction while taking aspirin or NSAIDs. It is also relevant to investigate whether the patient has suggestive complaints or a confirmed diagnosis of allergic rhinitis, atopic dermatitis, or eosinophilic esophagitis in his clinical history.^185^, ^186^

Ideally, these patients should always be evaluated by a multidisciplinary team to avoid delays in the correct diagnosis and treatment. It is up to ENT professionals to deal with the diagnosis of CRSwNP. Symptoms can be affeered through a visual analog scale, showing the patient’s perception of the severity of the symptoms, total or separately. A score above or equal to 5 means severe and uncontrolled disease. An extremely important symptom in patients with AERD is the progressive loss of smell, which usually shows non-sustained improvement with oral corticosteroids. It is essential to apply the psychophysical test of smell, so, the patient understands that this loss may be more serious than he realizes. It is worth mentioning that anosmia is one of the criteria established by the main guidelines to recommend treatment with biologics.^4^, ^185^, ^349^

The ideal order of investigation would be first to apply the smell test and the SNOT-22 questionnaire. The patient must answer a series of 22 questions about symptoms, sleep quality, and mood changes, describing the existing problem, whether it is very mild, moderate, severe, or very severe, scoring from 0 to 110. The result clearly shows how much the disease is interfering with the patient’s quality of life.^155^, ^185^ Next, nasal endoscopy, reveals the presence of edema, secretion, and nasal polyps. Therefore, the Nasal Polyp Scale (NPS), which has been widely used, can be applied, or even the Lund & Kennedy score,^166^ is more complete because it evaluates edema of the nasal mucosa, presence of secretion, and polyps. These scores are important for assessing the severity and lack of control of the disease, as well as helping to choose different treatments.

During nasal endoscopy, biopsy of polyps can be done if the patient has not been on oral, topical, or inhaled corticosteroids for at least 30 days. It should always be done with fenestrated forceps avoiding crushing the sample and sent to the pathologist, asking for eosinophils count per high-power field, obtaining an average of at least three high-power fields. 10 cells or more/HPF is currently accepted as the cut-off for tissue eosinophilia, characterizing a disease with a type 2 immune response.^4^

To confirm the disease and evaluate the extension into the paranasal sinuses, non-contrast CT should be performed in patients between acute exacerbations, in axial, coronal, and sagittal sections. The most used method for this study is the Lund & Mackay scale.^350^

Multidisciplinary care is critical for the management of patients with AERD. Specialists in allergy, immunology, or pulmonology can help in the correct diagnosis of asthma. Patients present with bouts of wheezing or wheezing, which can be confirmed by spirometry, demonstrating bronchial obstruction reversible with bronchodilators, in addition to investigating the presence of allergic sensitization, particularly to inhalant allergens such as house dust mites, animal allergens such as cats and dogs, cockroaches, pollens, and fungi. This investigation is carried out through immediate hypersensitivity skin tests (prick tests or, more rarely, intradermal tests) and/or specific IgE dosing for the most common inhalant allergens in each area. Values equal to or greater than 3.5 kU/L in the ImmunoCAP system are considered high. The measurement of total IgE also gives useful information in defining IgE-mediated mechanisms as part of the pathogenesis of the disease, and values equal to or greater than 100 IU/mL are considered high. Finally, peripheral blood eosinophilia obtained by blood count can also inform about a type 2 immune response.^5^, ^141^ Absolute eosinophil count above 300 mm^3^ is considered peripheral eosinophilia, but some guidelines consider the value of 150 mm^3^ to be elevated in patients with severe asthma. Other biomarkers have been researched, including serum periostin assay, *S. aureus* enterotoxin-specific IgE, and cytokine assay, but are not yet part of the clinical practice routine.^185^, ^349^

Allergists, immunologists, or pulmonologists can cooperate in the diagnosis and treatment of asthma with clinical history, physical examination, and pulmonary function test (spirometry) evaluating pre- and postbronchodilator parameters.^350^ When available, Fractional exhaled Nitric Oxide (FeNO) levels are correlated with Interleukin-13 (IL-13) levels, and both are indicators of type 2 inflammation in the airways.^351^, ^352^ Objective tools such as the Asthma Control Test (ACT) and the Asthma Quality of Life Questionnaire (AQLQ) or mini-AQLQ are used to assess patients’ quality of life, as well as periodic measurements of Peak Expiratory Flow.^185^, ^351^, ^352^ Asthma is a very important component of AERD and should be diagnosed and treated appropriately by specialists. According to the studies by Sella *et al*^353^ and Gill *et al*,^354^ asthma was the only factor related to a worse prognosis of CRSwNP after clinical treatment, leading to revisional surgeries, and a negative impact on the patients.

#### Appropriate medical treatment of AERD

The treatment of AERD should be personalized and multidisciplinary, addressing the various components of the disease: asthma control, management of CRSwNP, aspirin desensitization when necessary, and therapy with biologics. The goal is to improve the patient’s quality of life, reduce symptoms, prevent exacerbations, and limit the need for future surgeries.

#### Surgical treatment of AERD

AERD represents a severe phenotype of CRSwNP, with increased sinonasal inflammation, severe asthma, anosmia, and poorer health-related quality of life when compared with CRS without AERD.

Conventional treatment strategies often fail to control this recalcitrant form of airway inflammation. Around 37% of patients require endoscopic revision sinus surgery within five years of the first procedure.^355^ Surgical treatment of CRSwNP associated with AERD represents a significant challenge, due to its recalcitrant nature, presenting a high rate of recurrence due to persistent chronic inflammation, and requiring extensive and radical surgeries.

#### Management of Asthma in AERD patients

Asthma treatment is carried out according to the recommendations of GINA – Global Initiative for Asthma (ginasthma.org), updated every year by asthma experts.^202^

#### Long-term aspirin therapy in AERD after desensitization

Treatment with aspirin after desensitization aims to induce tolerance to NSAIDs in patients with AERD.^356^ This is a very specialized procedure, which consists of the gradual and controlled administration of increasing doses of aspirin until the therapeutic dose is reached. Different protocols can be implemented, but always in a hospital environment and under the supervision of experienced professionals, trained with the technique, as there is a risk of hypersensitivity reactions.^4^

The treatment significantly reduces the size of nasal polyps, in addition to reducing asthma exacerbations and improving lung function in patients with AERD and coexisting asthma. It is a low-cost therapy that produces significant improvement in the life quality.^357^, ^358^, ^359^

The EUFOREA Panel recommends considering aspirin treatment after desensitization in patients with bilateral nasal polyposis who have asthma as a comorbidity and a history of aspirin/NSAID intolerance, especially if NSAIDs are required for the treatment of chronic inflammatory conditions.^360^

Desensitization with aspirin followed by continuous treatment at doses of 325–650 mg of aspirin twice daily, is considered the standard care for patients with AERD after surgery (3–4 weeks prior). Aspirin can be discontinued for up to 48 h without loss of desensitization.^361^ Studies show that desensitization reduces the need for surgical interventions from three to nine years, despite potential gastric and hemorrhagic complications in some patients.^362^, ^363^ This procedure is contraindicated in patients with uncontrolled asthma, pregnant women, with a history of eosinophilic esophagitis, peptic ulcers, coagulopathies, or bleeding disorders.^364^

Although the clinical benefits of aspirin desensitization are evident, the underlying mechanisms are not yet fully elucidated. The required dose to improve bronchial inflammation is usually higher than that required to initiate a respiratory reaction or to maintain desensitization. Patients with AERD usually have elevated levels of leukotrienes, as measured by urinary LTE4, decreasing after desensitization. Other findings include reduced expression of cysteinyl LT receptor 1 (cysLT1) in nasal submucosal cells and inhibition of IL-4 production in T-cells.^365^

#### Treatment of AERD with biologics

The treatment of CRSwNP and AERD with biologics marks a significant evolution in the therapeutic approach to these conditions, targeting the specific modulation of inflammatory pathways of type 2 inflammation.

**Table:** 

Aspirin or non-steroidal aintiinflammatory drugs (NSAID) exacerbated respiratory disease: AERD
• A syndrome characterized by the triad: late-onset asthma, chronic rhinosinusitis with nasal polyps and hypersensitivity to COX-1 inhibitors (aspirin and NSAIDs). • Predominance of type 2 response with increased eosinophils in the tissue and serum. • AERD phenotype is associated with extensive polyposis, severe hyposmia, and worse prognosis for recurrence. • Treatment must be individualized and multidisciplinary. It is based on asthma control, CRSwNP management, aspirin desensitization and therapy with biologics. • Surgical treatment should be extensive and radical (complete) due to the high recurrence rates of the disease.

## The ABR recommends that every patient with CRSwNP must be investigated for NSAID intolerance and the presence of asthma

### Eosinophilic Granulomatosis with Polyangiitis (EGPA)

Eosinophilic Granulomatosis with Polyangiitis (EGPA) is a necrotizing vasculitis affecting small and medium-sized blood vessels. EGPA is characterized histopathologically by extensive eosinophil infiltration, causing organ damage due to inflammation and tissue ischemia. This condition was first reported by Churg and Strauss in 1951,^366^ and is now recognized as an Antineutrophil Cytoplasmic Antibody (ANCA)-associated vasculitis.^367^ The incidence ranges from 0.5 to 4.2 cases per million people per year, and its prevalence is between 10 and 14 cases per million population^368^, ^369^ and, the 5-year survival rate is estimated at 60–97%.^370^ The development of EGPA is divided into three stages. The first stage is the prodromal, characterized by asthma and chronic rhinosinusitis, and usually lasts from 3 to 10 years. The second stage is the eosinophilic stage, in which eosinophilia and eosinophilic infiltration into target tissues and organs can be observed. The third stage is the vasculitis stage, which presents with clinical manifestations consistent with vasculitis, such as palpable purpura and peripheral neuropathy. However, these phases often overlap, or do not necessarily develop in the sequence and, some patients may not manifest vasculitis complications.^367^, ^369^

The majority (>90%) of patients with EGPA are affected by asthma, which usually appears in adulthood, with rare seasonal exacerbations, and tends to worsen over time. Asthma is often accompanied by chronic rhinosinusitis with nasal polyps and, in some cases, recurrent otitis media. Clinical suspicion of EGPA should be raised when patients with airway manifestations develop other complications, including pulmonary infiltrates (40%–50%), which are often multiple and migratory, and respond to treatment with systemic corticosteroids. Peripheral neuropathy occurs in 50%–70% of patients, and has a pattern of mononeuritis multiplex, is usually sensory, but can also cause motricity deficits and has an axonal damage pattern.

Skin lesions are also frequent but quite heterogeneous. Palpable purpura is the most specific lesion of vasculitis. It is common for patients to also experience nonspecific symptoms, including malaise, weight loss, fever, and myalgia throughout the phases of the disease.^371^, ^372^, ^373^, ^374^, ^375^

Patients with EGPA have characteristically serum eosinophilia greater than or equal to 1000 cells per/μL (absolute value). Anti-MPO-ANCA (anti-neutrophil cytoplasmic antibody directed against myeloperoxidase) (P-ANCA) is detected in 30%–35% of patients with EGPA. Currently, EGPA pathophysiology can be divided into two phenotypes, vasculitic and eosinophilic, based on ANCA positivity.^376^, ^377^

Specifically, vasculitic features, glomerulonephritis, peripheral neuropathy, and purpura, occur more frequently in ANCA-positive patients, while eosinophilic features, such as cardiac involvement, gastroenteritis, and airway changes, are more frequent in ANCA-negative patients. However, vasculitic and eosinophilic phenotypes are not clearly separated, as most patients manifest an overlap between vasculitic and eosinophilic features.^367^, ^369^

Due to the difficulty in making the diagnosis, the American College of Rheumatology (ACR) and the European Alliance of Associations for Rheumatology (EULAR) created, in 2022, diagnostic criteria for EGPA. These criteria are designed to be used and have validity in research studies. The main objective was to differentiate cases of EGPA from similar cases of vasculitis.^378^

The criteria include seven clinical items that can be easily assessed during the routine evaluation of EGPA patients. When a cumulative score of 6 or more points is achieved, a patient diagnosed with small or mediumvessel vasculitis can be classified as having EGPA, with a sensitivity of 85% and a specificity of 99% (Table 13).^378^

**Table 13:** Diagnostic Criteria for EGPA (ACR/EULAR).^378^

Maximum eosinophil count ≥1000 cells per/μL	+ 5
Obstructive airway disease	+ 3
Nasal polyps	+ 3
Extravascular Inflammation predominantly eosinophilic	+ 2
Multiple mononeuritis and/or motor neuropathy unrelated to radiculopathy	+ 1
Positive pattern for cytoplasmic ANCA on immunofluorescence or anti-Proteinase 3 (PR3) ‒ ANCA (C-ANCA)	−3
Haematuria	−1

These criteria comprise parameters with a positive score, i.e., a maximum eosinophil count ≥1000 cells per/μL (+5-points), obstructive airway disease (+3), nasal polyps (+3), predominantly extravascular eosinophilic inflammation (+2), and mononeuritis multiplex and/or motor neuropathy unrelated to radiculopathy (+1), make the diagnosis of EGPA most likely.^378^

On the other hand, some parameters make the probability of EGPA less likely and are therefore scored negatively; these parameters include a positive pattern for cytoplasmic ANCA on immunofluorescence or antiproteinase 3 (PR3)-ANCA (C-ANCA) (−3) and hematuria (−1).^379^

The negative items underscore that these criteria are intended to be used as classification criteria, rather than diagnostic criteria, to differentiate EGPA from other forms of vasculitis in research settings. Both hematuria and anti-PR3-ANCA (C-ANCA) are negative items in the new EGPA classification criteria, although glomerulonephritis and ANCA are features of the disease that, when present, may be useful for diagnosing EGPA.^379^

When compared to other forms of vasculitis, biopsy showed that glomerulonephritis was significantly less common in patients with EGPA (4.9%) compared to those with Granulomatosis with Polyangiitis (GPA) (27.8%) or Microscopic Polyangiitis (MPA) (48.5%). Similarly, antiPR3-ANCA (C-ANCA) antibodies have been reported in few patients with EGPA, being much more prevalent in GPA. Thus, these antibodies received a negative score in the final criteria, because, despite being present in some EGPA phenotypes, they are significantly more prevalent in other vasculitis, thus increasing the sensitivity and specificity of these classification criteria for EGPA.^380^ Although antiANCA-MPO-ANCA (P-ANCA) antibodies can be detected in 30 %–35 % of patients with EGPA, the positivity of this antibody was not included in the final criteria because they are significantly more prevalent in MPA (microscopic polyangiitis) and, therefore, are not determinants of EGPA.^381^

The treatment of EGPA can vary according to the severity of the case, as well as during the maintenance, induction, or relapse phases. Treatment options include pulse therapy with corticosteroid, azathioprine, mycophenolate mofetil, cyclophosphamide, or rituximab, and, more recently, biologic drugs, especially mepolizumab (see biologics section).^382^

The diagnostic assessment of patients with suspected EGPA should always be multidisciplinary; other eosinophilic disorders and vasculitis have to be excluded, as well as the main complications of the disease, particularly cardiac, respiratory, cutaneous, renal, and nervous system involvement P-ANCA and eosinophilia should be investigated. Biopsy is recommended when feasible but is not essential for the diagnosis of EGPA.

**Table:** 

Eosinophilic Granulomatosis with Polyangeitis (EGPA)
1 EGPA is a necrotizing vasculitis that affects small and medium-sized vessels and extensive eosinophil infiltration. 2 The clinical picture consists on adult-onset, non-seasonal asthma and chronic rhinosinusitis with polyposis. Some cases may present glomerulonephritis, recurrent otitis media, peripheral neuropathy and skin lesions. 3 Patients have serum eosinophilia >1000 mm^3^ and may be ANCA positive. 4 Diagnosis consists of a combination of clinical and laboratory criteria. 5 Treatment may include systemic corticosteroids, azathioprine, and biologicals.

**The ABR recommendation is that patients with CRSwNP and serum eosinophilia > 1000 μL should be investigated for EGPA.**

### Chronic rhinosinusitis with nasal polyps type 2

Chronic Rhinosinusitis with eosinophilic Nasal Polyps (eCRSwNP) is clinically diagnosed by the presence of sinonasal symptoms for more than three months and by visualization of polyps in the nasal cavity. This condition is characterized by a type 2 inflammatory pattern, evidenced by eosinophilia and elevated levels of cytokines such as interleukin IL-4, IL-5, and IL-13. Manifests bilaterally and when unilateral, alternative diagnoses such as other non-eosinophilic polyps (e.g., choanal polyps), inverted papilloma, or nasal tumors should be considered.^383^

The prevalence of CRSwNP ranges from 1% to 4%, with a large difference between races and geographic regions, being more common in Caucasians and Western populations. The condition is diagnosed primarily in young and middle-aged adults, with a mean age of 39-years, no significant difference between the sexes, and a high association with a diagnosis of asthma.^384^

Although most cases of CRSwNP are idiopathic, it can also occur as part of genetic, metabolic, or immunological diseases. Other phenotypes of chronic diffuse rhinosinusitis may present with nasal polyps.

#### Physiopathology

CRSwNP is a complex disease, involving different types of immune responses, especially type 2 inflammation, which plays a central role in the disease.^385^ This type of inflammation is mediated by several cells and cytokines, which promote fibrin deposition, retention of plasma proteins, and edema, generating nasal polyps.^386^ Type 2 inflammation is characterized by the predominance of type 2 (Th2) helper-T lymphocytes, type 2 Innate Lymphoid Cells (ILC2), eosinophils, basophils, mast cells, and the production of specific cytokines such as IL-4, IL-5, and IL-13. This immune response is triggered by different stimuli, such as the presence of allergens, bacterial and viral infections, and results in the production of Immunoglobulin E (IgE) by specific plasma cells.

Eosinophils play a central role in the pathophysiology and severity of CRSwNP, serving as important biomarkers for the diagnosis and staging of the disease. A specific phenotype within CRSwNP, the eosinophilic CRSwNP (eCRSwNP), is associated with more severe symptoms, greater refractoriness to treatment, and a higher chance of recurrence after surgery.^387^, ^388^ eCRSwNP mainly affects people between 30 and 50 years of age and may present with acute sinonasal exacerbations, loss of smell, and late-onset asthma, responding well to oral and topical corticosteroids. Increased eosinophil levels may also be associated with other CRSwNP phenotypes, such as AERD.

Despite the predominance of type 2 inflammation, there is a large geographic variability concerning endotypes.^385^ In Eastern countries, there is a higher prevalence of type 1 and 3 inflammation, associated with neutrophilia, although this inflammatory profile has apparently been changing in recent years.^389^, ^390^ Even with the predominance of type 2 inflammation in the United States and Western Europe, endotypes vary geographically.^385^ In Brazil, a recent multicenter study evaluated the inflammatory profile of patients with CRSwNP. The results indicated that type 2 inflammation, characterized by the presence of eosinophils above 10 per high-magnification field (EPOS2020 criterion), is predominant in about 80% of patients. However, neither in the general group nor in this eosinophilic group was a preponderance of Type 2 cytokines indicate mixed inflammation, a fact corroborated by the high level of tissue neutrophils found in these patients. Hierarchical clustering was performed, and two well-defined groups of patients were identified, one with discharge and one with low inflammation, but both mixed. The best cut-off to define the two groups was that of 43 eosinophils per high-magnification field in the polyp biopsy. The group with more than 43 eosinophils had more severe disease.^175^

#### Symptoms and diagnosis

CRSwNP substantially impairs the quality of life of patients, especially during exacerbations, with an impact comparable to chronic lung and heart diseases.^391^ The most common symptoms are nasal obstruction and reduced sense of smell, often associated with sleep disorders and posterior nasal discharge. The severity of nasal obstruction is well correlated with the size of the polyps but does not predict the intensity of other symptoms. Headache and facial pain may also be present but are less frequent compared to other types of CRS since mucosal thickening in this condition is not usually associated with chronic infection.^392^ Other reported symptoms include fatigue, malaise, cough, pain or pressure in the ear, dizziness, halitosis, dental pain, dysphonia, and nasal or throat irritation.

Assessment of the severity of sinonasal symptoms and other systems impacted by CRS should be performed routinely. The main instrument used is the Sinonasal Outcome Test (SNOT-22) quality of life questionnaire^154^ whose unprecedented validation for Brazilian Portuguese was published in 2011.^155^ It is a tool that allows diagnosing, grading, and monitoring 22 symptoms associated with CRS.

Another way to grade and access the cardinal symptoms Of CRS is through a Visual Analogue Scale (VAS).

Patients should also be asked about lower respiratory symptoms and whether nasal or respiratory symptoms are exacerbated by ingestion of salicylates (present in nonsteroidal anti-inflammatory drugs and foods such as fresh fruit and nuts).

Confirmation of the diagnosis of nasal polyps usually requires nasal endoscopy, although anterior rhinoscopy may allow visualization of larger polyps. However, it is important to reaffirm that serial CT scans are NOT recommended for treatment evaluation and/or disease monitoring.

The density of polyps on CT is like thickened mucosa, although polyps differ from ordinary mucosal thickening by their shape and contours.

Psychophysical evaluation of olfactory function is also recommended, since loss of smell, together with nasal obstruction, is one of the main complaints in CRSN. In Brazil, the most commonly used tests are the UPSIT (University of Pennsylvania Smell Identification Test),^164^ the CCCRC (Connecticut Chemosensory Clinical Research Center) test,^163^ and, the recently validated Multiscent-20 (MultiScent-20 Digital Odour Identification Test).^162^ In addition to the diagnosis and gradation of the severity of olfactory loss, these tests allow a semisubjective evaluation of the olfactory capacity, enabling the diagnosis of changes before and after treatment or disease progression.

The diagnosis of CRS endotype has gained increasing importance. Serum and tissue eosinophil counts are being progressively used for the diagnosis of type 2 inflammation, and prognostic purposes.^185^ Serum eosinophil counts higher than 150 cells/mm^3^ suggest eosinophilia, whereas counts above 300 cells/mm^3^ generally indicate a worse prognosis. Tissue eosinophils ≥10 cells/HPF suggest eosinophilia, whereas counts above 43 cells indicate a pattern of high inflammation.^175^, ^185^ IgE counts above 100 IU/mL can also be used as a biomarker of type 2 inflammation, although its usefulness has been questioned in some studies.^175^, ^185^

#### Medical treatment in non-operated patients

In the era of individualized and precision medicine, it is important to consider the distinct endophenotypes for appropriate medical therapy. Therapeutic possibilities must be offered to the patient, so that a shared decision can be adopted, increasing the adherence to the treatment. Due to a better knowledge of the disease’s mechanisms, the therapeutic arsenal for the treatment of CRSwNP has achieved great advances in recent years, especially regarding the adequate extension of surgery and the advent of biologics.^3^, ^185^

Saline solutions have a beneficial effect on CRS with or without polyps since they help with the mechanical removal of secretions, pollutants, and pathogens, and improve the mucociliary beat and the sun layer of the mucus, contributing to optimize the mucociliary clearance.^393^, ^394^ Several devices are used for the application of saline solution, such as droppers, syringes, continuous jets, sprays, squeeze bottles (high-volume bottles), and neti pots. The indication is individualized, depending on age, socioeconomic conditions, and patient’s needs.

As for concentration, saline solutions can be isotonic or hypertonic.^394^

Patients with CRSwNP may have benefits with intranasal corticosteroids, such as improvement of symptoms, and reducing the size of polyps and recurrences.^395^, ^396^ In general, long-term use is considered safe and effective.^397^, ^398^ Molecules of high absorption such as dexamethasone should be avoided, as the risks outweigh the benefits.

Short courses of oral corticosteroids (5–21 days), added or not to intranasal corticosteroid treatment, have positive results in improving the sense of smell and other symptoms and reducing the size of nasal polyps. However, due to adverse effects, they should be restricted to up to 2 cycles per year.^4^ Frequent use of oral corticosteroids carries higher risks than the surgical treatment.^399^ Injectable depot corticosteroids should NOT be used as medical treatment for CRS.

Short-term oral antibiotics, i.e., a treatment course for less than four weeks, has an uncertain efficacy in patients with CRSwNP, even in exacerbations.^182^, ^400^ Its action can have greater benefits when guided by culture. Studies with topical and intravenous antibiotics have shown unclear results, so they are not formally recommended.^3^, ^4^

The benefits of antihistamines are well-established for associated allergic symptoms, however, there is no evidence to recommend them in CRSwNP. Nasal decongestants are not indicated for CRSwNP.^3^, ^4^

Leukotriene receptor antagonists are not indicated for the treatment of CRSwNP, except when associated with ASRD.^401^

There is no definition in the literature about what would be the best therapeutic regimen and its duration.^3^, ^4^ In this sense, the term “maximum” clinical treatment (which would be the association of numerous treatments for several cycles in order to avoid surgical treatment) has been replaced by the term “Appropriate” Medical Treatment (AMT), as it better reflects the optimization of treatments in an individualized way, without postponing a possible surgical treatment. Although there are numerous studies evaluating the effectiveness of individual classes of drugs in the treatment of CRS, there are no clinical trials evaluating the optimal combination of drugs.^3^

**The ABR recommends the association of the following therapeutic modalities: nasal saline, intranasal corticosteroid, and short courses (up to 2× per year) of oral corticosteroids as appropriate medical treatment for patients with CRSwNP. In cases of failure of the AMT (including frequent use of oral corticosteroids), surgical treatment should be indicated.**

## Surgical treatment of rhinosinusitis

Surgery plays a very important role in the management of chronic rhinosinusitis. It can be curative in cases such as fungal ball and odontogenic sinusitis, or it can provide control of the disease when combined with optimized postoperative medical treatment, as seen in cases of Aspirin-Exacerbated Respiratory Disease (AERD).^3^, ^4^, ^402^, ^403^

There are different etiologies for the chronic sinonasal disease, and many are not well known, which makes surgical management a real challenge. In addition, the anatomy of the paranasal sinuses is very variable among patients, as is the extent of sinonasal disease. Finally, the available equipment for performing the surgery, as well as the experience of the surgeons, varies significantly among different care centers. Thus, there is no gold standard surgical approach that can be applied to all cases of chronic rhinosinusitis.^3^, ^4^

1 Relieve symptoms and improve quality of life.
2 Decrease the inflammatory “load” (inflamed mucosa).
3 Widen the drainage pathways of the paranasal cavities.
4 Allow the penetration of drugs for topical treatment of the paranasal sinuses.
5 Allow objective monitoring of the sinonasal mucosa by nasal endoscopy in an outpatient setting.

Establishing the objectives to be achieved with the surgery in agreement with the patients seems to be a very attractive way to address these issues. The objectives of endoscopic sinonasal surgery in the management of chronic rhinosinusitis should be: a) The removal of diseased tissues; b) The modification of the sinonasal anatomy in order to allow aeration and drainage of secretions from the paranasal sinuses with expansion of the natural drainage pathways; and c) To enable topical medications for treatment of the affected cavities, which are so important in the continued management in many cases. In addition, enabling the control and follow-up of patients in an outpatient set, especially through nasal endoscopy. Table 14 summarizes the main objectives of the surgical treatment in CRS.

**Table 14:** Main surgical objectives in CRS.

1. Relieve symptoms and improve quality of life
2. Decrease the inflammatory “load” (inflamed mucosa)
3. Widen the drainage pathways of the paranasal cavities
4. Allow the penetration of drugs for topical treatment of the paranasal sinuses
5. Allow objective monitoring of the sinonasal mucosa by nasal endoscopy in an outpatient set

At the present time, most ENT schools guide surgical procedures with the basic premise of preserving the sinonasal mucosa as much as possible.^3^, ^4^ Although most patients report improvement in quality of life after surgery, follow up and continued medical treatment are essential for managing their condition.^403^

### Preoperative

#### Surgical indication

Among the main surgical indications for patients with rhinosinusitis, we should mention the following conditions and respective reasons^3^, ^4^:

1 Complications (orbital, intracranial, or bone) secondary to ARS or eventually acute exacerbations of CRS.
2 Recurrent acute ARD, refractory to medical treatment.
3 Secondary CRS: elimination of the underlying factor causing sinonasal inflammation, such as fungal balls, odontogenic sinusitis, tumors (e.g., antrochoanal polyp), and neoplasms.
4 Correction of postoperative or iatrogenic complications, such as nasal synechiae, lateralization of the middle turbinate, and recirculation due to the presence of accessory ostia.
5 Primary CRS: refractoriness to clinical treatment, characterized by the non-improvement or persistence of poor indicators of quality of life or specific sinonasal symptoms related to CRS, confirmed by the presence of inflammatory changes on nasal endoscopy or computed tomography.

#### Timing for surgical indication

Once the patient is referred for a surgical procedure, it should not be postponed to minimize the negative impact on the patient’s quality of life and postoperative evolution.^404^ In the healthcare system, patient pathways must be optimized to avoid unnecessary delays in surgery.^3^

Sinus endoscopic surgery, which addresses both rhinosinusitis and its complications, may serve as a definitive treatment or be part of a combined medical and surgical therapeutic plan”.

#### Preoperative evaluation and care

Patients with Chronic Rhinosinusitis (CRS) who are recommended for surgery require a thorough preoperative evaluation and preparation to ensure a precise and safe procedure.

Some preoperative measures are essential to improve the anatomical understanding of the structures to be addressed, to properly plan the surgical strategy, to reduce bleeding, to preserve normal tissues, and to avoid complications.

It is important to evaluate medications in the preoperative period to optimize the surgical field. There are some medications that increase intraoperative bleeding. Stopping medications such as anticoagulants and/or aspirin/NSAIDs, garlic, ginger, ginkgo biloba should be sought. Although we are not aware of any study in sinus endoscopic surgery that shows a difference, a meta-analysis indicated that the risk of reoperation for postoperative tonsillectomy hemorrhage is 7.2-times higher in the aspirin group.^405^

In addition, it is important to control other comorbidities that may interfere with the intraoperative and postoperative periods, such as bronchial asthma. In particular, the lack of asthma control can impair the execution of surgery and is associated with a six-fold increased risk of postoperative recurrence of CRSwNP.^406^ In eosinophilic patients with CRSwNP with asthma, sinus surgery can positively affect asthma control by suppressing systemic/airway inflammation.^407^

#### Preoperative medications

##### Corticosteroids

In the preoperative evaluation, it is essential to identify whether the patient can use corticosteroids perioperatively, since the inflammation increases bleeding during surgery. Perioperative corticosteroids have been shown to reduce intraoperative bleeding, shorten surgical time by nearly 14 min, and improve the quality of the surgical field.

This effect was observed in a study with intranasal corticosteroids for four weeks and with oral corticosteroids for 15-days before surgery. It is unclear whether there is an additive effect on systemic corticosteroids plus intranasal corticosteroids.^408^, ^409^

**The ABR recommends intranasal corticosteroids for at least 4-weeks prior to endoscopic surgery 4 or shortterm oral corticosteroids.**

Current recommendations advocate preoperative corticosteroids in patients with CRSwNP undergoing endoscopic sinus surgery, however there is no consensus on optimal dose or duration. There was no statistically significant difference in the condition of the surgical field, intraoperative blood loss or surgical time among various dosing regimens. Because there is no evidence-based definition of dosage and duration, further studies are needed to evaluate the efficacy of a low-dose preoperative regimen with the goal of reducing the patient’s cumulative exposure to systemic corticosteroids.^410^ For oral corticosteroids, 0.5–1 mg/kg of weight within 7 days with or without progressive dose reduction over days is a commonly prescribed regimen.^3^

##### Antibiotics

There are no studies on the effect of preoperative oral antibiotics for CRSwNP. Peric *et al* demonstrated that macrolides can decrease polyp size, but the role of preoperative antibiotic therapy for CRSwNP needs further investigation.^411^

**Therefore, the ABR does not recommend the use of antibiotics in the preoperative period of endoscopic sinus surgery.**

#### Preoperative paranasal sinus CT

Sinus CT scan with multiplanar imaging is essential for a thorough preoperative assessment of the anatomy. This is necessary not only to confirm the presence and extent of the disease, but also to identify any anatomical variations that may increase the risk of complications.

There are several established methods for systematically interpreting preoperative imaging. The CLOSE mnemonic is widely used for this purpose (see Table 15).^412^, ^413^

**Table 15:** CLOSE method for preoperative tomographic evaluation.

C	**C**ribriform plate cleft – asymmetry, depth
L	**L**amina Papiracea – dehiscence
O	**O**nodi ‒ Spheno-ethmoid cells
S	**S**phenoidal sinus – carotid/optical dehiscence
E	**E**thmoidal Arteries – anterior and posterior

#### Informed Consent Form (ICF)

It is important to discuss the patient’s expectations before surgery, including the likely outcomes, risks, benefits, the importance of postoperative follow-up for optimal recovery, as well as postoperative medications and topical treatments. The patient’s consent should be documented in the ICF.

Table 16 summarizes the main preoperative care for endoscopic sinus surgery.

**Table 16:** Preoperative care in endoscopic sinonasal surgery.

• Detailed analysis of the computed tomography images (paying special attention to areas of greatest surgical risk).
• Discontinue acetylsalicylic acid or other non-hormonal anti-inflammatory drugs.
• Discontinue medications that alter blood clotting (e.g. Ginkgo biloba, antiplatelet agents and other anticoagulants).
• Emphasize the need for periodic postoperative endoscopy-guided monitoring and care.
• Discuss the treatment strategy (postoperative nasal washes with corticosteroids, immunobiologicals, among others), risks and benefits.
• Sign a specific informed consent form.

### Surgical techniques

Paranasal sinus surgery has advanced significantly with the routine use of endoscopic techniques. The imaging precision provided by endoscopes, including wide-angle optics (0 °, 30 °, 45 °, and 70 °), allows for detailed visualization of the nasal cavities, sinuses, and surrounding vital structures. Additionally, the development of specialized instruments for sinonasal dissection has enabled more precise and controlled surgeries. Despite these technological advances, surgical training and anatomical knowledge continue to be the key determinants of a successful outcome.

Among the various surgical techniques for CRS, there is no universal “gold standard” applicable to all cases. Due to the lack of randomized controlled trials, several aspects of surgical management remain controversial and are based on observational cohort studies.^414^ One of the most controversial topics is the extent of surgical dissection.^355^, ^415^, ^416^ Consequently, current guidelines, which rely largely on case series and expert opinion, recommend that surgical management should be tailored to each individual patient.^3^, ^4^

The most commonly used and recommended surgical approach is endonasal. However, some cases may require external or combined access, such as in the case of lateral frontal sinus lesions, or when safe anatomical landmarks or appropriate instruments for an exclusive endonasal approach are unavailable, or even based on the surgeon’s experience. Regardless of the technique and instruments used, there is clearly a learning curve in endoscopic sinonasal surgery that must be respected.^417^

Next, the main endoscopic techniques described for the surgical treatment of CRS will be briefly reviewed.

#### Endoscopic Sinus Surgery (ESS)

Endoscopy-assisted transnasal sinus surgery, better known as Endoscopic Sinus Surgery (ESS), is the most widely used technique for the surgical treatment of CRS. The term “functional”, first proposed by David Kennedy and Heinz Stammberger (Functional Endoscopic Sinus Surgery)^418^, ^419^ in the early 1980s, has been increasingly questioned over the last decade, and is now less commonly used. The term “functional” was used to differentiate endoscopic surgery from traditional surgeries, which were commonly performed externally, often not including the natural drainage pathways of the paranasal sinuses. In addition, nonendoscopic techniques advocated the systematic and complete removal of the sinus mucosa for the treatment of chronic rhinosinusitis, unlike “functional” surgeries. Despite ongoing debates surrounding certain aspects of Endoscopic Sinus Surgery (ESS), such as the extent of the procedure and the size of the drainage ostia, the principle of preserving normal sinonasal mucosa and addressing the natural ostia surgically is widely accepted in current literature.^3^, ^4^

The strategies and techniques for performing Endoscopic Sinus Surgery (ESS) vary widely across the world, whether in terms of anatomical approaches (centripetal vs. centrifugal technique), surgical sequence (anterior-posterior/inferior-superior), or even the instruments used.^419^, ^420^ However, the ultimate goal is to achieve a safe and successful surgical procedure. Given the challenges in conducting well-designed comparative studies and training, individual experience and the availability of resources should guide the choice of technique. Therefore, precise knowledge of the anatomical structures that constitute the ostiomeatal complex, the natural maxillary sinus drainage ostium, the complete configuration of the ethmoid cells, the frontal recess and its relationship with the corresponding sinus drainage, the sphenoethmoidal recess and the posterior ethmoid and sphenoid drainage pathways, are crucial for the success of CRS surgery, regardless of the surgical technique. In addition, a meticulous review of the CT scan in all three planes (coronal, axial, and sagittal) is crucial for effective surgical planning in every case.

#### Centripetal technique

The centripetal technique is an endoscopic surgery approach that can be applied for CRS prioritizing the gradual removal of diseased tissue, starting from the most external regions towards the central areas. The main philosophy behind this technique is the preservation of mucosa and sinus anatomy, which could theoretically reduce postoperative morbidity and facilitate the restoration of sinus function.^420^, ^421^

The technique differs from traditional surgical approaches to CRS by adopting a dissection methodology rather than resection. While conventional techniques often focus on identifying ethmoidal cells prior to resection, the centripetal technique focuses on initially identifying and preserving the orbit and skull base, proposing to remove ethmoidal cells only at the end of the procedure. This approach might enhance operative safety.^420^, ^422^

#### Extent of surgery

In cases of secondary chronic rhinosinusitis, such as an isolated maxillary or sphenoid fungal ball, surgical dissection can be directed to the affected region only, with enlargement of the corresponding drainage ostia to allow for complete cleaning and adequate aeration.

The most significant controversies occur in primary cases, where the exact cause of the disease is not known (e.g., CRSwNP and CRSwNP). The term “Full House” is still widely used to describe endoscopic surgery in which all paranasal sinuses are included in the dissection, from the anterior ethmoid sinuses to the skull base. This approach also involves opening the natural drainage ostia of the maxillary, frontal (Draf IIA), and sphenoid sinuses, with complete removal of diseased tissue and preservation of the normal mucosal lining.^4^

Modifications of conventional Endoscopic Sinus Surgery (ESS), such as the Minimally Invasive Sinus Technique (MIST), which involves a reduction in the extent of the dissection,^423^, ^424^ or the enlargement of the dissection in extended surgeries, can also be successfully performed in selected cases.^425^, ^426^

MIST (Minimally Invasive Sinus Technique) is used for surgery focused only on the transition space of the paranasal sinuses, i.e., the preostia space of the sinus natural drainage. In this technique, the unciform apophysis is removed, but the sinus ostia is not enlarged. The procedure is largely performed with the help of a microdebrider.^423^, ^424^

Balloon sinus dilation has also been successfully described in some cases and may be beneficial for patients with CRS without nasal polyps, particularly when the disease is limited to the maxillary sinus, with or without anterior ethmoid involvement. It is usually used in milder or less extensive cases, without involvement of the posterior ethmoids, the frontal or the sphenoid sinuses.^427^ However, higher-quality evidence is needed to assess the cost-effectiveness and efficacy of this technique compared to ESS in the management of CRS.^428^

Maxillary mega-antrostomy (Modified Endoscopic Medial Maxillectomy), Frontal sinus drill out (Modified Lothrop or Draf III), Sphenoid sinus drill out, are considered extended procedures and are usually reserved for recalcitrant or revisional cases.^425^, ^426^, ^429^ These techniques will be discussed in greater detail later in this chapter.

Studies show that enlarged maxillary, frontal, and sphenoid sinus drainage ostia are associated with increased penetration of nasal lavages.^430^, ^431^, ^432^ Thus, larger openings should be performed when one of the main objectives is to control intrasinus inflammation.^433^, ^434^ Furthermore, outpatient postoperative clinical monitoring can be better performed in the presence of larger openings of the paranasal sinuses.

Surgical techniques with a shorter dissection extent, such as MIST and balloon dilation, are considered short term treatment options for patients with less extensive CRS, particularly those with disease more localized to the ostiomeatal complex or maxillary sinus. For cases of moderate-severe CRS, with greater disease extension, traditional or extended ESS has the greatest potential for long-term sinus ventilation, as well as the best distribution of nasal lavages with topical medications.^4^ Furthermore, especially in cases of CRS with nasal polyps, despite the need for more robust evidence, studies show that complete surgery (Full House ESS), when compared to, for example, previous ethmoidectomy, significantly decreases the chance of recurrence.^355^, ^435^

#### Mucosal preservation versus resection

The preservation of the sinonasal mucosa in endoscopic surgeries has shown better postoperative results, with earlier reestablishment of local physiology. Resection of the sinonasal mucosa is an exceptional technique for the management of extensive CRSwNP.^4^ Direct comparison studies between mucosal preservation or resection techniques are necessary to reach a degree of recommendation.

Nasalization and Reboot Surgery are procedures that advocate, in addition to what has been described for Full House ESS, the removal of the sinus mucosa to a greater or lesser extent.^436^, ^437^

The nasalization technique was initially described in 1995 for the surgical treatment of CRSwNP,^436^ and is based on the communication of the entire ethmoidal and sphenoid complex, in addition to the careful resection of the entire non-olfactory mucosa of the ethmoidal complex, avoiding intraoperative complications and minimizing postoperative repercussions. This procedure, which corresponds to the complete ‘marsupialization’ of the ethmoid, with removal of all its mucosa and wide opening of the paranasal sinuses ‒ maxillary, frontal and sphenoid, into the nasal cavity, is known as “sinus nasalization”. The technique has recently been modified following the Theory of Evolution and Development (Evo-Devo) of the Nose^436^, ^438^ in which the ethmoid and paranasal sinuses are distinct organs.

More recently, a rereading of the philosophy of nasalization has been called Reboot Surgery^437^, ^439^ which consists of the complete removal of the inflamed sinonasal mucosa in cases of moderate-severe type 2 inflammation, associated or not with Draf III.

**The ABR recommends preserving the sinonasal mucosa during surgery whenever possible in all cases of CRS.**

#### Resection of the middle turbinate

The available evidence suggests that partial or total resection of the middle turbinate in patients with CRS should be individualized.^3^, ^4^ It can be performed, especially in patients with CRSwNP, but the risks and benefits must be considered in each case.^440^, ^441^, ^442^

The main potential benefits of partial or total resection of the middle turbinate are improved access and visualization of anatomical structures, enhanced reach and effectiveness of nasal lavages, reduction of local inflammation, delayed recurrence or nasal polyposis, and prevention of scarring adhesions. The main risks are epistaxis, cerebrospinal fluid fistula, iatrogenic obstruction of the frontal and middle meatus, alteration of smell, and loss of an anatomical reference in case of revisional surgery.

The main indications for middle turbinate resection by otorhinolaryngologists include: CRSwNP with involvement of the middle turbinate, cases of AERD (aspirin-exacerbated respiratory disease) and cases of allergic fungal rhinosinusitis. In CRSsNP cases, maximum preservation of the middle turbinate should be considered, both in primary surgeries and in revisional surgeries.^440^

#### Surgery of inferior turbinate

Surgery of the inferior turbinate is not considered treatment for CRS and, therefore, should not be performed routinely. In cases where intervention on the middle turbinate is necessary (e.g., in CRSwNP), addressing the ipsilateral inferior turbinate, in addition to offering no benefits, may lead to negative consequences for the patient.^443^ However, in cases of nasal obstruction due to inferior turbinate hypertrophy, regardless of the sinus condition (e.g., concomitant Allergic Rhinitis), conservative surgery on the inferior turbinates may be considered.

#### Primary surgery vs. revisional surgery

It is estimated that the need for revisional surgery in patients with CRS is around 5% of cases in a period of 5-years and up to 25% in a long-term followup.^444^, ^445^, ^446^

Although the anatomical changes found in revisional cases can present a technical challenge, the surgical principles and objectives remain the same in both primary and revisional procedures: opening the natural ostia and removing diseased tissue to facilitate drainage and allow better access for topical medications within the affected sinuses. In some cases, however, the anatomical alterations found in revisional surgeries may pose a significant challenge, as the loss of anatomical landmarks increases the risk of intraoperative complications or less precise surgical execution. In such cases, the classical anatomical landmarks (such as the uncinate process, ethmoid bulla, basal lamella of the middle turbinate, and superior turbinate) should be replaced by alternative structures, like the superior turbinate, choanal arch, and papyraceous lamina.^447^ When available, a surgical navigator can be particularly useful in these challenging cases.^448^

#### Extended surgical access to the frontal sinus

The best surgical approach for CRS depends on the severity of the disease, the patient’s anatomy, and other individual factors. In some situations, more extensive procedures, such as extended endoscopic approaches (e.g., Draf IIB or Draf III) or combined external approaches, may be necessary. The decision about the type of surgery should be individualized and based on the careful evaluation of the patient by a specialized surgeon, considering the severity of the disease, the specific nasal anatomy, and the possible complications associated with each procedure.^449^

The indications for extended frontal sinus surgeries can be seen in the Table 17.^430^, ^449^, ^450^, ^451^

**Table 17:** Indications for extended surgical approaches to the frontal sinus.

Reason	Justification
Failure of previous treatments	When the disease persists, especially in the frontal recess region, despite appropriate medical treatment and less invasive surgical procedures, such as endoscopic paranasal sinus surgery type Draf IIa.
Stenosis of the frontal ostium	In cases where there is significant stenosis of the frontal ostium, limiting adequate drainage of the frontal sinuses and contributing to the recurrence of symptoms of chronic rhinosinusitis or mucocele formation.
Complex anatomy	Prominent intersinus septations, reduced anteroposterior diameter, or obstructive frontoethmoidal cells requiring a more extensive surgical approach to improve drainage and access to the affected sinuses.

The decision to perform the Draf III procedure using the “inside-out” or “outside-in” approaches depends on the patient’s anatomy and the width of the frontal recess. Below are some considerations for choosing each approach^451^:

Inside-Out: Indicated when the anteroposterior diameter of the front recess is wide enough to allow access to surgical instruments, usually greater than 4–5 mm. This procedure is usually performed after the complete endoscopic sinus surgery, including maxillary antrostomy, ethmoidectomy, and sphenoidectomy. It involves the total removal of the uncinate process, enlargement of the frontal recess, and creation of a septal window to connect the frontal sinuses. The “inside-out” approach is the standard technique when the anatomy allows sufficient access to the frontal recess.

Outside-In: Indicated when the front recess is narrow and cannot easily accommodate surgical instruments or drills.

Resection of the anterior portion of the frontal bone (frontal beak) is performed, and the anterior limit is the exposure of the periosteum. Upon entry into the frontal sinus, the neoostium is expanded laterally to include the frontal recesses. In general, the “outside-in” approach is used when the patient’s anatomy does not allow a safe performance of the “inside-out” technique due to a narrow frontal recess.

The most significant postoperative complication following the Draf III procedure is stenosis of the frontal opening, which can occur up to 2-years after surgery, requiring revisional surgery in 5%–30% of cases. Periodic outpatient debridement, combined with continuous use of intranasal corticosteroids, may reduce the risk of stenosis.

External approaches to the frontal sinus are surgical procedures performed outside the nasal cavity to access and treat conditions of the frontal sinus. These approaches are indicated in specific cases where endonasal techniques are inadequate or insufficient for treating frontal disease. Below are some of the external approaches to the frontal sinus^449^:

1 Upper eyelid incision
2 Transorbital corridor
3 Frontal osteoplastic flap
4 Cranialization of the frontal sinus
5 Removal of the anterior wall of the frontal sinus (Riedel)
6 Complete frontal sinus ablation (Riedel-Mosher)

Despite advances in endoscopic techniques and instrumentation, the frontal sinus remains a challenging area due to the narrowness of the frontal recess, the need for specialized angled instruments, and its close anatomical relationship with critical structures. These factors increase the risk of complications and surgical failures.

#### Extended approaches to the maxillary sinus

There are several extended endoscopic approaches to the maxillary sinus, each with its own anatomical limitations and potential complications.^452^ Megaantrostomy plays a key role in the surgical treatment of refractory inflammatory diseases and sinonasal neoplasms. The prelacrimal approach provides excellent access to the maxillary sinus, though it may be limited by anatomical variations. Denker’s transseptal and endoscopic approaches do not significantly increase maxillary sinus exposure compared to other methods and are associated with higher morbidity.^452^

##### Maxillary mega-antrostomy

Endoscopic mega-antrostomy, also known as Endoscopic Modified Medial Maxillectomy (EMMM), was first described in 1996.^425^ Mega-antrostomy is a procedure designed to provide expanded surgical access to the maxillary sinus. It involves enlarging a conventional maxillary antrostomy to allow for better access to the sinus, facilitating sinus cleaning (including nasal aspiration or lavage), improving access for topical medications (such as corticosteroids or antibiotics), and promoting gravity-dependent drainage.^425^ This procedure is typically recommended as a surgical option for refractory CRS or for better endoscopic access to extensive benign tumors, such as juvenile nasopharyngeal angiofibroma, antrochoanal polyps with anteroinferior insertion, inverted papillomas, and odontogenic cysts. Mega-antrostomy can also be beneficial in patients with impaired mucociliary clearance, such as those with cystic fibrosis or primary ciliary dyskinesia. It is considered a safe procedure with low revision rates, and the most common complication is nasolacrimal duct injury, occurring in 0 %–4 % of cases.^425^

##### Prelacrimal approach

Initially described by Zhou *et al* in 2013,^453^ the approach to the maxillary sinus through the prelacrimal recess allows direct access to the sinus with 0-degree endoscopes, preserving the inferior turbinate and the nasolacrimal duct. This approach involves a vertical incision in front of the inferior turbinate along the lateral nasal wall, just behind the piriform aperture. After lifting a mucosal flap over the bone, the anteromedial wall of the maxillary sinus can be removed anteriorly to the nasolacrimal duct using an osteotome or drill, providing direct access to the maxillary sinus.^453^, ^454^ At the end of the procedure, the mucosal flap is repositioned and sutured with absorbable material. Access to the sinus can be further widened by moving the inferior turbinate/medial wall of the maxillary sinus medially into the nasal cavity.^453^, ^454^ Additional access can also be obtained by enlarging the the piriform aperture/anterior wall of the maxillary sinus. Complications such as collapse of the alar cartilage has been described.^452^

The advantage of this technique is that it provides direct access to the anterior wall of the maxillary sinus without the need to reposition or excise the nasolacrimal duct. Additionally, the inferior turbinate and medial wall of the maxillary sinus may return to their original anatomical position by the end of the procedure, minimizing functional morbidity associated with the removal of the lateral nasal wall.^453^, ^454^ The prelacrimal recess approach is particularly valuable for the removal of recurrent antrochoanal polyps, benign tumors, and refractory fungal infections, as well as for approaches to the pterygopalatine and infratemporal fossae.^452^

#### Extended approaches to the sphenoid sinus

Some situations require a wider opening of the sphenoid sinus, such as in cases of sphenoid fungal rhinosinusitis or significant osteoneogenesis, to ensure successful treatment.

Extended access to the sphenoid involves the removal of the entire anterior wall of the sinus. Lateral limit is the orbit, medially, the nasal septum, superiorly the skull base, and inferiorly, the floor of the sphenoid sinus which is at the level of the choanal ridge.

To avoid injury to the septal artery that crosses the anterior wall of the sphenoid, inferior to the natural ostium of the sinus, a mucosal flap can be elevated, exposing part of the bone, keeping the mucosa and artery intact.

More extensive cases may require bilateral procedures involving the removal of part of the posterior nasal septum and the sphenoid rostrum to create a single bilateral aperture.

#### External surgical approaches for RS treatment

The advancement of endoscopic sinonasal surgery in recent decades has led to the gradual abandonment of external surgical approaches to the paranasal sinuses, particularly for the treatment of rhinosinusitis. External approaches are primarily indicated when endonasal access is insufficient, especially in the lateral regions of the frontal and maxillary sinuses, or when the opening of the natural drainage pathways does not adequately address the disease.^450^, ^455^

Another reason for opting for external surgical approaches, even in the era of endoscopic sinonasal surgery, is the presence of complications from acute rhinosinusitis, such as superior, inferior, and lateral subperiosteal abscesses, as well as frontal and maxillary osteomyelitis, particularly when the disease cannot be reached endoscopically.^456^ It should be noted that we are only considering indications for external surgical access in the treatment of rhinosinusitis.

In inflammatory diseases, although success rates are similar between endoscopic sinonasal surgery and external surgical approaches, the latter should be considered a last resort due to its higher associated morbidity.^455^ For non-medial subperiosteal abscesses, external surgical access is the treatment of choice, since the endonasal endoscopic surgery does not allow complete drainage of the abscess most of the time. It is interesting to note that the endoscopic sinonasal drainage surgery does not seem to add benefit to the external drainage of the non-medial subperiosteal abscess.^457^ In situations in which the surgeon finds feasible to reach the non-medial subperiosteal abscess by endoscopic endonasal route, this access can be performed, provided that the abscess is properly reached and drained.^458^

### Surgical complications

The rates of sinonasal surgical complications resulting from endoscopic approaches have decreased substantially with the dissemination and better training of techniques over the last 4 decades, from 2 %–17 %^417^, ^459^ to less than 1% in the last decade.^460^, ^461^

Complications are generally classified as minor and major (Table 18). Most published case series report that minor complications are the most common.

**Table 18:** Complications of sinonasal surgeries.

Minor complications	Major complications
Toothache	Anosmia
Subcutaneous Orbital Emphysema	CSF leak
Local infection	Orbital Hematoma
Persistent Crusts	Meningitis
Synechiae	Severe Trans or Postoperative Nasal Hemorrhage (Large Vessels)
Epiphora	Intraorbital OR intracranial bleeding
Stenosis of the Natural Drainage Ostium	Visual Changes
	Brain Tissue Trauma
	Death

Cases requiring revisional surgery, particularly those with extensive disease, sinonasal and skull base anatomical alterations, dehiscence areas related to the disease or previous surgeries, or the use of power instrumentation (e.g., microdebriders), are more prone to complications. If any of these complications arise intraoperatively, they should be promptly identified and repaired, sometimes with the support of a multidisciplinary team involving specialists such as neurosurgeons, ophthalmologists, or interventional radiologists. Postoperative complications should be managed according to their severity and impact on the patient.^456^, ^462^, ^463^, ^464^

### Postoperative

#### Postoperative prognosis

The prediction of postoperative outcomes based on clinical and laboratory features has been studied for decades in patients with CRS. The most commonly reported characteristics associated with poorer clinical outcomes (such as quality of life, symptoms, nasal endoscopy, and smell) include worse preoperative CT scores, a history of previous sinonasal surgery, asthma, NSAID intolerance, elevated serum and tissue eosinophilia, and increased levels of eosinophilic cationic protein and eosinophilic protein, among others.^353^, ^446^, ^465^, ^466^, ^467^, ^468^ These characteristics are crucial for aligning patients’ expectations with the likelihood of improvement, emphasizing the importance of treatment adherence and long-term clinical follow-up.

#### Postoperative care

The postoperative care of nasal endoscopic surgery aims to promote adequate mucosal healing, with clearence function recovery as early as possible. This reduces secondary infections, synechiae, and the uncomfortable symptoms for patients, such as nasal obstruction, discharge and unpleasant odor. General guidelines to reduce edema and bleeding include relative rest, with the head of the bed elevated for 24–48 h post procedure, and avoiding blowing the nose in the first few days after surgery. In addition, it is important to inform that nasal obstruction and minor bleeding may occur. Avoiding more intense physical activities in the first 2- to 4-weeks is also important and depends on the extent of the surgery and the addressed structures.

Postoperative follow-up on an outpatient basis is essential to monitor for possible secondary infections, hematomas, and the removal of nonabsorbable splints or hemostats, if necessary, as well as for surgical site debridement.

Clearance impairment persists for 3- to 12-weeks after surgery, and the presence of stagnant blood and mucus can act as a culture medium for microorganisms, perpetuating an inflammatory response. Debridement reduces the chance of colonization by removing secretion, clots, crusts, bone fragments, and adhesions, which can decrease postoperative inflammation and the potential for synechiae and lateralization of the middle turbinate. Debridement improves patients’ postoperative symptoms and the endoscopic appearance of the sinus cavities. Adequate topical anesthesia is essential to reduce patient discomfort, avoiding additional local trauma and the formation of new adhesions.

Postoperative debridement usually starts between 5–10 days after surgery, at weekly intervals or every 2-weeks, varying according to the patient’s symptoms, position of the middle turbinate, crust formation, signs of infection, and the severity of postoperative inflammatory findings.^469^ In a systematic review, postoperative debridement, when compared to non-intervention, reduced the rate of synechiae formation in the postoperative period, with no difference in symptoms or in the quality of life of patients in the long term.^470^ Additionally, studies suggest that aggressive daily debridement is no more effective than weekly or bi-weekly debridement, and it may be more uncomfortable for the patient.^471^, ^472^

Before starting debridement, it is important to alert and reassure the patient about the possibility of presenting a vagal reflex and hypotension, in addition to be prepared to recognizing warning signs such as sweating, skin pallor, dizziness, and even syncope. In these cases, the procedure should be interrupted, the patient should be positioned with the head down and legs elevated, vital signs should be checked, and an ice pack can be applied to the forehead.

**The ABR recommends that postoperative debridement be performed routinely. Their frequency and intensity must be individualized.**

#### Postoperative oral medications

Antibiotics in the immediate postoperative period of nasal endoscopic surgeries is frequent. In a survey conducted in 2018 by the American Academy of Rhinology, most rhinologists (62.3%) routinely used antibiotics in the postoperative period of endoscopic nasal surgeries.^473^ Despite this routine, there is a lack of robust evidence to support the use of antibiotics in the postoperative period. In a meta-analysis published in 2021, Swords *et al*,^474^ analyzed 1045 publications, including 5 randomized controlled trials. The studies did not demonstrate superiority of the use of antibiotics versus placebo on the symptoms reported by the patients or on the endoscopic scores. However, it should be remembered that the studies are heterogeneous in terms of the antibiotic regimens and duration of use. Even when nasal packing or splints are placed, the use of antibiotics is questionable in the literature. Unfortunately, there are no studies comparing the outcomes of patients with nasal packing or splints, with and without prophylactic antibiotics. The studies evaluate the duration of antibiotics, without demonstrating an increase in local infection rates. Toxic shock syndrome after nasal surgery is rare (16.5 cases per 100,000).^475^

**The ABR does not recommend the routine use of antibiotics in the immediate postoperative period following ESS for CRS. In patients showing signs of active infection (e.g., intraoperative purulent discharge), antibiotic treatment should be guided by culture results.**

A meta-analysis on the use of macrolides in the postoperative period did not show improvement in symptoms and quality of life scores (SNOT-22) compared to placebo, but demonstrated the possibility of improvement in endoscopic scores in a subgroup analysis.^476^ Another meta-analysis investigating predictive factors of good response to macrolides in CRS, demonstrated better results in the subgroup without nasal polyps compared to the group with polyps; in half-dose subgroup compared to the very low dose, and in period of 24-weeks compared to 8‒12-weeks.^477^ Bezerra *et al* used macrolide in half-dose in patients with CRSwNP who failed medicalsurgical treatment, and obtained better results in the subgroup with low serum IgE.^478^

Due to the lack of evidence on the use of macrolides in the postoperative period, there is no certainty about their long-term safety, nor about the phenotypic or demographic characteristics related to the prediction of responses to this type of therapy.

The ABR considers the use of low-dose, long-term macrolides as an option in late postoperative CRS patients, especially in those without polyps and low IgE, and with corticosteroid failure.

Oral corticosteroids are commonly prescribed in the immediate postoperative period of patients with CRS due to their anti-inflammatory properties, but they are medications with risk of various side effects. In a 2016 systematic review with meta-analysis,^479^ it was found that systemic corticosteroids in the postoperative period did not improve patients’ symptoms. However, there was an improvement in the endoscopic score and in the recurrence rate of polyps in patients with CRS and nasal polyps. In 2021, Chang *et al*^480^ conducted a randomized clinical trial evaluating postoperative oral corticosteroids in CRS patients without nasal polyps, showing that there was no improvement in symptoms or endoscopic scores when compared to the placebo group. As the studies are generally heterogeneous and with small samples, it suggests that more studies should be conducted to prove the benefit of this strategy.

The ABR recommends that oral corticosteroids in the immediate postoperative period be used judiciously and individualized. In patients with CRScPN, oral corticosteroids can be used as rescue medication in the late postoperative period, with a maximum of two short cycles per year.

#### Nasal lavage with saline solution

Nasal lavage with saline solution plays an important role in the postoperative period of ESS due to its mechanisms of action, such as cleaning mucus and crusts and reducing mucosal edema. For these reasons, it is recommended to improve symptoms and nasal endoscopy outcomes. Nasal lavage should be initiated early, ideally 24–48-h after surgery.^3^, ^4^, ^481^

Saline solutions can vary in terms of tonicity; isotonic (0.9%) or hypertonic (2% or 3%). Hypertonic solutions have greater potential for adverse events such as discomfort and burning. In addition, they can vary in pH and should ideally have a neutral or slightly alkaline pH (pH between 7 and 9), as buffered solutions are better tolerated.^54^, ^482^ There is no consensus regarding the ideal tonicity. One study compared isotonic saline, hypertonic, and lactated ringer’s wash in patients with CRS in the immediate postoperative period of ESS (6-weeks), and observed a significant reduction in SNOT-22 and visual analog scale in patients who used lactated ringer’s wash compared to the other two solutions.^483^ On the other hand, a systematic review with meta-analysis found no differences in symptom and endoscopic scores when comparing lavage with isotonic, hypertonic, and lactated ringer’s saline solution in the postoperative period of ESS.^484^

High-volume nasal lavage promotes greater distribution to the operated paranasal sinuses than nasal spray.^432^ Despite this, there are no data to prove that high-volume nasal lavage is more effective than low volume in the immediate postoperative period. Studies comparing the squeeze bottle with the nasal spray in the early postoperative period of ESS, demonstrated significant improvement in SNOT-22 and nasal endoscopy with both devices.^485^, ^486^ Despite the scarcity of studies, clinical experience shows that high-volume devices (such as the squeeze bottle, the neti pot, and the nozzle) are a good option for postoperative patients with ESS, due to their adequate penetration into the newly operated paranasal sinuses and a more pronounced mechanical action in removing debris. In addition, low-pressure devices (such as the neti pot) have less risk of adverse events such as otalgia and aural fullness. In Brazil, the use of syringes is widespread, and 60 mL or more is considered high-volume. This method provides similar penetration into the paranasal sinuses as the squeeze bottle in the postoperative period, particularly when the middle turbinate is sutured to the nasal septum during surgery (Bolger’s point).^241^

Therefore, nasal lavage with saline should be adopted in the immediate postoperative period of ESS and the device should be chosen individually, being accessible and comfortable to the patient, preferably high-volume and low-pressure devices. The frequency of nasal lavage should be higher in the first postoperative days (usually 4- to 6-times a day) and gradually reduced depending on the intensity of the symptoms. In addition, the patient must be instructed of how to apply the device correctly, ensuring safety and adherence.

**The ABR recommends that nasal lavage with saline solution be routinely used in the postoperative period of ESS, ideally with high volume.**

#### High volume nasal irrigation with saline and corticosteroid

Although the role of high-volume nasal lavage with saline/corticosteroids in controlling CRS in postoperative patients is well-established, promoting long-term improvements in symptoms and nasal endoscopy,^487^, ^488^ few studies have evaluated the effects of this combination in the immediate postoperative period following ESS.

Budesonide and mometasone are the most common used corticosteroids. The clinical and endoscopic superiority of high-volume nasal lavage with 2 mg of mometasone compared to mometasone spray was observed in patients undergoing ESS, followed for 12-months. The endoscopic superiority of high volume saline nasal lavage with 1 mg of budesonide compared to nasal lavage with saline solution alone was evidenced at a 3-month postoperative follow-up of ESS.^488^, ^489^

In Brazil, to circumvent the high cost of commercial formulations of budesonide and mometasone for highvolume lavage, Kosugi *et al* introduced 1% manipulated budesonide in drops, demonstrating significant results in endoscopic and symptoms improvement with this manipulated solution when compared to corticosteroid spray in operated patients, with better results with the daily dose of 1 mg (2 drops/day).^52^, ^490^

**The ABR recommends high-volume saline nasal lavage with corticosteroids in both the immediate and late postoperative periods for patients with CRScPN, while it is optional for other cases. Depending on clinical monitoring, the frequency or dosage may be reduced or replaced with an intranasal corticosteroid spray.**

#### Xilitol

Studies that evaluated the efficacy of 5% xylitol nasal lavage (12 g of xylitol in 240 mL of water) in patients with CRS in late follow-up of ESS, reported better outcomes compared to saline in reducing SNOT-22 and improving symptoms.^491^, ^492^ A more recent Brazilian study compared high-volume saline lavage with xylitol in high-volume lavage associated with saline solution used for 30-days in 52 patients in the immediate postoperative period of ESS, and found significant improvement in pain symptoms and nasal symptoms in the xylitol group (assessed by the visual analog scale, SNOT-22 and NOSE questionnaire [Nasal Obstruction Symptom Evaluation]).^322^

Despite the lack of studies, according to the ABR, the addition of xylitol for high-volume nasal lavage should be considered in the immediate postoperative period of ESS. In CRSsNP (non-type 2), saline nasal lavage with the addition of xylitol may be considered as a long-term treatment option.

#### Intranasal topical antibiotics

Topical nasal antibiotics were no more effective than placebo in improving CRS symptoms and, in addition, caused irritation, epistaxis, and bacterial resistance.^3^, ^4^ A randomized clinical trial^320^ involving patients in the immediate postoperative period of ESS evaluated the use of topical antibiotics compared to oral antibiotics (study group: absorbable sponge soaked in bacitracin; control group: absorbable sponge soaked in saline solution + oral antibiotic). The study found no differences in the rate of postoperative infection, though it did not include a control group without antibiotics.^493^

**The ABR does not recommend the use of topical antibiotics in the immediate postoperative period of ESS.**

#### Intranasal decongestant

Nasal decongestants were not superior to placebo (xylometazoline vs. saline spray) in improving symptom scores (nasal obstruction, rhinorrhea, pain, smell changes, and bleeding) in a study that evaluated 47 patients 10 days postoperatively after ESS.^494^ Due to the well documented risks of using this type of drug, such as rhinitis medicamentosa and rebound phenomenon, topical decongestants should not be routinely used in the postoperative period of ESS. International consensuses recommend against its use.^3^, ^4^

**The ABR does not recommend the use of intranasal decongestants in the immediate postoperative period of ESS.**

#### Other topical substancies

Baby shampoo: it is an antiseptic solution. Studies that looked at the effectiveness of baby shampoo compared to saline, including a direct postoperative study, did not observe differences between treatments, but reported more side effects and discontinuation of treatment with baby shampoo.^495^, ^496^ In addition, baby shampoo caused nasal obstruction and change in smell in healthy individuals.^497^ Therefore, its use in the postoperative period of ESS is not recommended.

Honey: one study compared nasal lavage with the addition of Manuka honey to saline lavage for 30-days postoperatively and found no significant differences in SNOT-22, nasal endoscopy, and bacterial culture between groups.^498^ Due to the low quality of the evidence, the addition of honey to saline lavage is not recommended in the postoperative period of ESS.

Sodium hyaluronate: it is a natural polysaccharide (sodium salt of hyaluronic acid). A randomized clinical trial that evaluated 56 patients with CRS without polyps in the postoperative period of ESS (3- and 6-weeks), comparing nasal lavage with the addition of sodium hyaluronate to nasal lavage with saline solution, found significantly better results in the 3rd week in the NOSE questionnaire and in the symptom score in the sodium hyaluronate group, but found no difference between treatments after 6-weeks.^499^ Thus, despite few studies, the addition of sodium hyaluronate to nasal lavage with saline solution may have a positive effect on the immediate postoperative period of ESS.

Dexpanthenol: is an analog of pantothenic acid with a potential effect on healing. One study looked at the use of dexpanthenol spray compared to saline spray in 50 patients postoperatively (for 6-weeks) and found no significant differences in symptoms and nasal endoscopy between treatments, but there was improvement in clearance and mucociliary with dexpanthenol. Due to the scarcity of studies, there is no recommendation for the use of dexpanthenol in the postoperative period of ESS.^500^

**The ABR does not recommend the addition of these alternative components to high-volume nasal lavage in the immediate postoperative period of ESS.**

#### Corticosteroid-releasing stents

Despite several clinical and surgical options for the treatment of CRSwNP, none has yet achieved complete resolution of the condition, and there remains a percentage of patients who are resistant to all the discussed treatments.^3^, ^4^

The method of administration, the vehicle of the medication, the total cost of treatment, and the potential side effects must always be considered

In view of this scenery, and with the technological advancement of the pharmaceutical industry, a new option has been used in the USA since 2011: slow-release corticosteroids implants. This option is not yet approved by ANVISA in Brazil. In the latest consensus updates, the EPOS2020^4^ and ICAR-RS-2021^3^ refer to the use of topical corticosteroid implants as beneficial for the treatment of CRSwNP, with level 1A of evidence.

Since 2011, Propel® has been cleared by the FDA, since showed efficacy and safety in more than 140 patients evaluated in 11 U.S. centers.^501^, ^502^ Propel® is a stent that differs from other products; as a biodegradable corticosteroid-release device. Over 90-days follow-up, the implants significantly reduced the rate of restenosis/occlusion and the need for postoperative interventions when compared with surgery alone. In addition, other benefits have been reported, such as reduction of polyp formation, synechiae, and edema of the mucous membranes of the paranasal sinuses, playing an important role in the treatment of patients with recurrent CRSwNP after sinus surgery.^503^

More recently, a larger, second-generation sinus implant, with higher corticosteroid concentration and longer drug release duration than its predecessor, was approved by the FDA for CRSNwNP patients with prior ESS, as an alternative to revision surgery due to polyp recurrence: SINUVA™ Sinus Implant received FDA approval in December 2017 for in-office treatment of recurrent CRSwNP.^504^ The safety and efficacy of in-office implantation have been demonstrated by several clinical studies.^505^, ^506^

In 2021, Rivelli *et al*,^507^ a group from Brazil, published a study using biode-gradable polymeric nanofibers containing mometasone furoate as a novel approach for drug delivery to treat CRSwNP by providing controlled steroid administration to the sinonasal mucosa. In vivo tests, showed that the level of inflammation in the mucosa of animals that received the nanofiber with mometasone furoate was lower than those that received the nanofibers without the drug. Histopathological analysis showed that polymeric nanofibers containing mometasone is a safe method when applied topically to the sinonasal mucosa, opening a new horizon in the treatment of CRSwNP.^507^

A systematic review with meta-analysis published in 2024 suggested that implants with corticosteroids in the middle meatus improve ESS outcomes by decreasing rates of adhesion, postoperative medical and surgical interventions, recurrences and inflammation, without having a significant negative impact on the immediate postoperative period. However, more research is needed on the long-term effects.^508^

## Biologics in the treatment of chronic rhinosinusitis

Biological drugs targeting type 2 inflammation have been transforming the treatment for CRSwNP patients. Especially for severe and difficult-to-control cases, these drugs have brought a new paradigm, allowing the effective and safe treatment of extensive diseases that were previously not completely controlled with the conventional strategies like surgery and topical medications. However, the high cost of biologics makes their judicious use to avoid overbur-dening a health system that is already overloaded while ensuring they remain accessible to those who really need it.

Biologics are high molecular weight molecules with complex structures, produced in organisms or cultured living cells. They act on the immune system, comprising several classes, including vaccines, serums, immunoglobulins, monoclonal antibodies, fusion proteins, and human cytokines.^185^, ^509^, ^510^

Currently, in Brazil, three biologics are approved by the National Health Surveillance Agency ‒ ANVISA ‒ for the treatment of CRSwNP (omalizumab, mepolizumab, and dupilumab), and two more are under study with potential for future use. These five drugs will be discussed below:

### Available Biologics for the treatment of CRSwNP

#### Omalizumab

Omalizumab was the first biologic used for type 2 inflammatory diseases, receiving FDA (Federal Drug Administration) approval in 2003. In Brazil, omalizumab was approved by ANVISA for use in CRSwNP in 2021, and in the same year it was incorporated into SUS (via CONITEC) for the treatment of moderate-to-severe persistent allergic asthma uncontrolled with inhaled corticosteroids.

Omalizumab is a humanized anti-IgE monoclonal antibody that works by binding to free circulating IgE, inhibiting its attachment to the high-affinity IgE receptor and reducing free IgE levels. This leads to a secondary decrease in IgE receptor expression on mast cells, basophils, and dendritic cells.^511^

The POLYP1 and POLYP2 studies, phase 3 randomized double-blind, placebo-controlled multicenter clinical trials, achieved the primary endpoints with improvements in Nasal Polyp Score (NPS) and Nasal Congestion Score (NCS) compared to placebo. However, the NPS was statistically significantly different only in POLYP1 trial, where mean change in NPS was from -1.08 on the omalizumab-treated group versus 0.06 for placebo (p < 0.0001).^512^

There is only one randomized, controlled, prospective, blinded clinical study evaluating omalizumab for Allergic Fungal Rhinosinusitis (AFRS) over a 6-month period. All patients in this study underwent endoscopic sinus surgery, which may represent a significant bias. These limitations suggest the need for additional studies with longer followup periods and without adjuvant surgery to establish more robust treatment guidelines for AFRS.^513^, ^514^

The EVEREST trial is the first multicenter, phase 4, randomized (1:1), double-blind, controlled, head-to-head study comparing dupilumab and omalizumab. It is still in the collection data phase and the aim of the study is to compare efficacy and safety of these biologics over 24-weeks of treatment in patients with severe CRSwNP and associated asthma.^515^

The recommended dose of omalizumab varires depending on the IgE concentration and patient weight, ranging from 75 to 600 mg per dose administered every 2- or 4-weeks in adults.

The most common adverse events include headache, dizziness, abdominal pain, arthralgia, and injection site reactions. Less frequent side effects include pharyngitis, laryngeal edema, cough, allergic brocospasm, syncope, diarrhea, nausea, urticaria, angioedema, paresthesia, somnolence, myalgia, systemic lupus erythematosus, idiopathic thrombocytopenia, and anaphylaxis.

**The ABR considers omalizumab a treatment option for patients over 18-years of age with CRSwNP, especially in atopic cases with elevated IgE and persistent sinonasal symptoms after optimized clinical and surgical treatment.**

#### Mepolizumab

Mepolizumab was approved in 2017 by ANVISA as an add-on treatment for the maintenance control of severe eosinophilic asthma in adult patients. In 2021, it was incorporated into the Clinical Protocol and Therapeutic Guidelines (PCDT) as a treatment for severe eosinophilic asthma within the scope of the Unified Health System (SUS). In 2022, ANVISA expanded its approval to include CRSwNP refractory to clinical or surgical treatment. Mepolizumab is a humanized monoclonal antibody (IgG1, kappa), directed to human Interleukin-5 (IL-5) with high affinity and specificity. Mepolizumab inhibits the binding of IL-5 to its receptor on the eosinophil surface thereby inhibiting IL-5 signaling and reducing eosinophil production and survival.^516^

The SYNAPSE study, a phase 3, randomized, doubleblind, placebo-controlled clinical trial, reported a statistically significant improvement in the total nasal polyp score in patients treated with mepolizumab, as well as reduced need for nasal surgery and lower systemic corticosteroid usage.^516^

In 2023, an observational, retrospective, real-world study was conducted at a single center, involving 55 patients with severe CRSwNP treated with mepolizumab. Most patients (89%) had asthma, and 51% had Aspirinexacerbated Respiratory Disease (AERD). The study showed significant improvement in endoscopic nasal polyp scoring, visual analog scale of symptoms, Sinonasal Outcome Test score (SNOT-22), Asthma Control Test (ACT), blood eosinophil counts, and corticosteroid utilization.^517^

For patients with severe CRSwNP associated with elevated serum eosinophil levels, mepolizumab has a favorable safety profile because it can reduce eosinophilia.^518^ There is no defined standard on the number of serum eosinophil counts to be considered, which can range between 1,000–1,500 eosinophils/μL.

Mepolizumab is beneficial for patients with severe CRSwNP and demonstrated efficacy in treating EGPA (eosinophilic granulomatosis with polyangiitis). The MIRRA study (multicenter, double-blind, randomized, placebocontrolled, phase 3 trial) evaluated the efficacy and safety of mepolizumab (300 mg subcutaneous injection every 4-weeks) in combination with oral corticosteroid therapy in patients with relapsed or refractory EGPA. The study results demonstrated that, compared to placebo, mepolizumab treatment significantly reduced eosinophil counts, increased the proportion of patients in remission, and prolonged the duration of remission, reducing relapse rates. In addition, oral corticosteroid doses were successfully reduced in the mepolizumab group, and 18% of patients in the mepolizumab group achieved corticosteroid free remission during weeks 48 and 52.^519^ Based on these findings, mepolizumab was recommended for inducing remission in patients with relapsed or refractory EGPA and maintaining remission without the risk of life-threatening manifestations or organs involvements.^369^

For patients with CRSwNP, the recommended dose of mepolizumab is 100 mg every 4-weeks, while for EGPA, the dose is 300 mg every 4-weeks.

The most common adverse events associated with mepolizumab include headache, myalgia, infections (pharyngitis, lung, or urinary tract), abdominal pain, back pain, fever, eczema, injection site reactions, or systemic allergic reactions. Anaphylaxis is a very rare but potentially serious adverse event and patients should be alerted about this risk.

**The ABR considers mepolizumab an option in adult patients with severe CRSwNP and persistent sinonasal symptoms after medical and surgical treatment. It is also indicated for adult patients with relapsed EGPA or EGPA refractory to oral corticosteroid treatment.**

#### Dupilumab

Dupilumab is a human monoclonal antibody that inhibits the alpha subunit of the Interleukin-4 Receptor alpha (IL-4Rα). By blocking this receptor, it prevents the action of two key cytokines in type 2 inflammation, IL-4 and IL-13, which play critical roles in IgE synthesis, eosinophil release, mucus secretion, and airway remodeling.

Dupilumab was initially approved by ANVISA for the treatment of atopic dermatitis and asthma, and in 2020, it was approved for use in CRSwNP. Two randomized, double-blind, placebo-controlled, phase 3 multicenter studies, LIBERTY NP SINUS-24 and SINUS-52, evaluated the efficacy and safety of dupilumab in 276 and 448 patients, respectively, over 24- and 52-weeks. In both studies, dupilumab significantly reduced Nasal Polyp size (NPE – from 6 to 1), improved SNOT-22 score (from 58 to 11 points), decreased sinus opacification on CT scans, and improved the sense of smell in 1-year of follow-up compared to placebo. Adverse events led to treatment discontinuation in 4% of SINUS-24 patients and 11% of SINUS-52 patients, with arthralgia and persistent eosinophilia being the most common ones.^520^ The results validated the use of dupilumab for patients with refractory CRSwNP, confirming the efficacy and safety of the drug.

The DUPIREAL, multicenter Italian phase IV, real-life study further corroborated the efficacy and safety of Dupilumab. A total of 648 patients were followed up over 12-months, and patients in use of dupilumab demonstrated reductions in nasal polyp size (NPE from 6 to 1), decrease in SNOT-22 scores (from 58 to 11 points), and a significant recovery in the sense of smell (Sniff-in’Sticks scores from 4 to 12). Treatment was discontinued in 26 patients, 20 due to unsatisfactory responses and 6 due to adverse events (persistent hypereosinophilia and arthralgia).^521^

There is no ideal biomarker that guide the choice of biologic, nor are there published head-to-head studies directly comparing them. However, an indirect comparison of randomized clinical trials suggests that dupilumab showed superiority over omalizumab in reducing polyp size, nasal congestion, restoring the sense of smell, and improving symptoms, especially in patients with AERD.^522^ Another indirect comparison between the 3 biologics currently approved for CRSwNP found efficacy in all 3 biologics, with small differences among them.^523^

One question regarding the use of biologics is the possibility of increasing dose intervals in cases of controlled disease. A real-life study in a Dutch prospective cohort followed 131 patients who used dupilumab every 2-Weeks (2 W) for 24-weeks. For those who had a moderate to excellent response, the interval was extended to 4-Weeks (4 W). To compare with the pivotal study, evaluation parameters were similar, with the addition of olfactometry (Sniff-in’Sticks-12) and the Asthma Control Test (TCA) when applicable.

The mean scores of all outcomes showed improvement in both the standard dosage group (2 W) and the extended interval group (4 W).^524^ There is also the need for additional studies to corroborate the practice of increasing dosing intervals in clinically controlled CRSwNP.

Another issue exists in relation to the adjuvant effect of Dupilumab in combination with surgery, or comparative studies between these treatments. A systematic review of 5 randomized controlled trials (n = 1748 patients) showed that both biologics and ESS improved quality of life and symptom outcomes. Over a one-year period, Dupilumab was superior to surgery in improving SNOT-22 scores. ESS was more effective in reducing nasal congestion during the initial 6 months, being surpassed by dupilumab after that. In the evaluation of smell improvement, dupilumab was superior to other biologics and ESS.^524^, ^525^

The recommended dose for dupilumab is 300 mg every 2-weeks. Common adverse events include systemic hypereosinophilia, conjunctivitis, keratitis (which may lead to visual blurring), oral herpes, arthralgia, and injection site reactions. Very uncommon adverse events include facial rash, severe keratitis, dry eye, and hypersensitivity reactions (serum sickness reactions, characterized by fever, urticaria, arthritis, arthralgia, nephritis) anaphylaxis and angioedema.

The hypereosinophilia observed as side effect of dupilumab is usually transient. In a post-hoc analysis,^526^ of CRSwNP studies, there was a transient increase in serum eosinophilia up to 16-weeks of treatment, followed by a subsequent decrease. Levels exceeding 1500 eosinophils/μL during treatment were more frequent in patients with baseline eosinophilia above 500 eosinophils/μL. A similar pattern was observed in studies involving asthma and EGPA. In less than 1% of patients, transient hypereosinophilia was related to severe symptoms, which led to drug discontinuation.

**The ABR considers dupilumab an option for adult patients with CRSwNP with persistent sinonasal symptoms after appropriate medical and surgical treatment.**

### Biologics under study (not yet approved for CRSwNP)

#### Benralizumabe

Benralizumab was the second anti-IL-5 drug approved in Brazil for severe eosinophilic asthma in patients aged 18-years and older. It is a humanized monoclonal antibody (IgG1κ) that targets the alpha subunit of the IL-5 receptor, resulting in apoptosis of eosinophils and basophils via antibody-dependent cytotoxicity diminishing cells formation.^527^

The phase III OSTRO study evaluated the efficacy of benralizumab in patients with CRSwNP who did not improve symptoms after using intranasal corticosteroids for at least 4-weeks and history of oral corticosteroid use and previous surgical treatment.

The dosing regimen included three initial 30 mg subcutaneous doses administered every four weeks, followed by five doses every eight weeks. The final dose was administered at week 48. The mean total nasal polyp score showed a difference of −0.570 between the benralizumab and placebo groups (*p* < 0.001), and the mean biweekly nasal obstruction score difference was −0.270 (*p* = 0.005) at week 40. However, the mean reduction of 5.21 points in the SNOT-22 score at week 40 compared to placebo was not statistically significant (*p* = 0.81). Overall, reductions remained substantial at week 56, eight weeks after the final dose (*p* = 0.02).^528^ Based on these results, the FDA rejected licenseing approval for benralizumab for CRSwNP treatment.

Further real-world data are awaited to determine whether the observed statistical improvements are clinically significant. In addition, a new phase III trial, ORCHID is currently underway to evaluate the safety and efficacy of benralizumab compared to placebo in patients with CRSwNP (clinical-trials.gov, NCT04157335). This trial is expected to be completed in 2025, and will evaluate outcomes over 56-weeks, as opposed to the 40-weeks conducted in OSTRO.^528^ Unlike OSTRO, ORCHID study only includes patients with eosinophil blood counts >2% or ≥150 μL (clinicaltrials.gov, NCT04157335).

In patients with severe eosinophilic asthma and CRSwNP as a comorbidity, benralizumab has been shown efficacy and safety. The ANDHI study, a phase IIIb, randomized, double-blind, placebo-controlled trial, evaluated the impact of benralizumab on HRQoL, exacerbation rates, and lung function, with post-hoc analyses focused on patients with CRSwNP. The study included adults (18–75 years of age) with severe eosinophilic asthma, at least two exacerbations in the previous year despite high doses of inhaled corticosteroid plus additional controllers, with an eosinophil count of at least 150 cells/μL, and an Asthma Control Questionnaire-6 (ACQ-6) score of 1.5 or greater. The primary efficacy endpoint was the annual asthma exacerbation rate, while secondary measures, including SNOT-22, were evaluated only in patients with a clinical diagnosis of CRSwNP.^529^ Among 656 patients, 427 received benralizumab, and 229 received placebo. Benralizumab significantly reduced asthma exacerbation risk compared to placebo and showed a statistically significant improvement in SNOT-22 scores from baseline to week 24.^529^ These results were later validated in realworld studies.^530^, ^531^, ^532^

The most common adverse reactions tp benralizumab include headache, sore throat, fever, injection site reactions, and hypersensitivity reactions (itching and skin irritation). Severe allergic reactions, including anaphylaxis, may also occur.

Benralizumab is not currently indicated for the treatment of CRSwNP but is approved for severe eosinophilic asthma in adult patients.

#### Tezepelumab

Tezepelumab is a human monoclonal antibody that blocks Thymic Stromal Lymphopoietin (TSLP), a cytokine originating from epithelial cells that serves as an inducer of pro-inflammatory pathways. TSLP has been implicated in the pathophysiological processes associated with severe asthma and CRSwNP. Tezepelumab has been evaluated in phase III studies, named NAVIGATOR and SOURCE for patients with asthma.^533^, ^534^

The post hoc analysis of patients with CRSwNP who participated in the NAVIGATOR study demonstrated a mean reduction of 21 points in SNOT-22 scores at week-52 among patients treated with tezepelumab.^534^ A phase III, double-blind, placebo-controlled, randomized study called WAYPOINT is currently ongoing to specifically evaluate the efficacy of tezepelumab for the treatment of CRSwNP (clinicaltrials.gov, NCT04851964).

The most common adverse reactions associated with Tezepelumab include pharyngitis, arthralgia, injection site reactions, and hypersensitivity reactions (itching and skin irritation). More severe allergic reactions may occur, including anaphylaxis.

At present, tezepelumab is not approved for the specific treatment of CRSwNP. It is indicated for the treatment of severe asthma in patients aged 12-years and older.

### When to indicate a biological for CRS?

In 2024, to assist physicians in recommending biologics for CRS, the Brazilian Academy of Rhinology published an updated version of its Guidelines, introducing a score designed and validated by its authors.^185^ The score determines which patients have the most precise indications for biologics and is based on the following criteria:

1 The impact of the disease on the patient’s quality of life.
2 The extent of sinonasal disease.
3 The presence of comorbidities associated with the type 2 inflammation (such as asthma and AERD).
4 The presence of biomarkers indicative of type 2 inflammation

For a patient to be eligible for biologics, two essential conditions must be met:

• The presence of CRSwNP;
• The patient must have undergone appropriate prior surgery (or have absolute contraindication to the surgical procedure).

Once these two criteria are confirmed, the scoring system below (Table 19) should be applied.

**Table 19:** Scoring system for indication of biologics in CRSwNP. Values equal to or greater than 14-points correspond to a possible benefit to the patient.

Variables	Points
*Sub-item - Symptom severity (1)*	
a) SNOT-22 (validated to Portuguese)	
<20	0
20‒50	1
>50	2
b) AVS for nasal obstruction/congestion or rhinorrhea (consider the worst)	
<3	0
3‒7	1
>7	2
c) OlfactoryTest	
<3 (normosmia or mild hyposmia)	0
3‒7 (moderate hyposmia)	1
>7 (severe hyposmia or anosmia)	2
d) Number of previous surgeries	
0	0
1	1
2	2
≥3 or contraindication to surgery	3
e) Use of systemic CE/year	
0	0
1 or 2	1
>2	2
*Sub-item ‒ Extent of the disease*	
f) Extent of polyps (Nasal Polyp Scores ‒ bilateral)	
0	0
1 to 2	1
3 to 4	2
>5	3
g) CT scan Extension (Lund-Mackay ‒ bilateral)	
0 to 4	0
5 to 8	1
9 to 16	2
>16	3
*Sub-item ‒ Comorbidities*	
h) Asthma	
No	0
Mild	1
Moderate/Severe	2
i) NSAID intolerance	
No	0
Yes	2
*Sub-item ‒ Biomarkers*	
j) Serum eosinophil count	
<150	0
150‒300	1
>300	2
k) Tissue eosinophil count	
<10	0
10 to 43	1
>43	2

This scoring system evaluates 11 parameters, assigning scores based on the specific criteria, with a total possible score ranging from 0 to 25 points. During the initial validation process, 58 patients participated (29 with indications for biologics and 29 without), and statistical tests were applied to assess the sensitivity of the scoring system and determine the ideal cut-off to distinguish between these groups. The cut-off score deemed ideal was 14 points (ROC curve with AUC = 0.9828, sensitivity of 0.96 and specificity of 0.93; Cronbach’s alpha of 0.84).

Based on the parameters and the current criteria, patients with a score of 14 or higher may benefit from biologics, while those with a score below 14 are not indicated for their use.

When a biologic is prescribed to treat severe uncontrolled CRS, it is essential to monitor the patient’s response to the medication.

The ABR recommends reevaluation after one and three months of treatment, including a blood count, assessment of adherence, and monitoring for adverse events. In the 6th month of treatment and every 6-months there-after, the effectiveness of the biologic should be reviewed.

The following criteria should be considered: VAS for nasal obstruction and/or SNOT-22, use of oral corticosteroids, reduction of Nasal Polyps (NPS), olfactometry, and asthma control (Table 20). If the patient shows improvement in at least four of the five criteria, the response is considered good. If improvement is observed in up to three criteria, the response is considered moderate, and treatment should be maintained in both cases. However, if none of these criteria are met, the patient is considered non responder.

**Table 20:** Response criteria to treatment with biologics.

1. Improvement in quality of life (SNOT 22): >9 points and/or AVS: <2 points in nasal obstruction
2. Reduction in the use of oral corticosteroids (up to one cycle per semester)
3. Improvement in smell (Up-sit/Connecticut): at least one degree
4. NPS: reduction >2 points
5. Asthma control: absence of exacerbations
Types of responses:
4‒5: good
1‒3: moderate
None: non-responder

For patients with good or moderate response, the recommendation is to continue the treatment. In cases of non-response, the option is to switch to other biologic or consider surgery.^185^

**The ABR recommends that biologics be considered a treatment option for patients with severe CRSwNP who remain uncontrolled after adequate surgery, demonstrate adherence to postoperative treatment, and achieve a score > 14 based on the ABR indication criteria, while always considering cost-effectiveness.**

## Chronic rhinosinusitis in children

### Introduction

It is estimated that in pediatric patients the prevalence of Chronic Rhinosinusitis (CRS) ranges between 2.1% and 4%, and that children aged 10- to 15-years are the most affected.^4^, ^535^

Children’s CRS differ from adults’ CRS in several respects: developing immune system, exposure to the environment of a growing sinus system. In addition, they have a different inflammatory mechanism (adults with CRS without polyps typically manifest a type 1 inflammatory response and polyps type 2, and children with CRS have higher numbers of neutrophils and macrophages and rarely an increase in tissue eosinophils).^320^, ^536^, ^537^

CRS alone is uncommon in children, and in most cases, comorbidities including allergy, cystic fibrosis, primary ciliary dyskinesia, and immunodeficiency should be assessed years.^320^

Although with little evidence, the pathophysiology of paediatric CRS appears to involve both genetic and environmental factors. Thus, in monozygotic twins, both siblings do not always develop polyps, indicating that the occurrence of nasal polyps can be influenced by both genetic and environmental factors.^4^

It is not yet possible to endotypically characterize paediatric CRS, although some studies have shown upregulation of inflammatory markers in sinus tissues and nasal wash products in children with CRS.^4^

Inflammatory cytokines are present in the sinus tissues of children with CRS and are more abundant when there is concomitant asthma. Submucosal glandular hyperplasia appears to be the characteristic phenotype in pediatric CRS and MUC5B, the predominant glandular mucin.^536^

Regarding cellular infiltrate in children with CRS, it was observed that in children under 13-years of age, there is conflicting evidence about the predominance of neutrophilic or eosinophilic inflammation. Studies comparing CRS of adults and children showed that neutrophilic inflammation was more prevalent in children. Increased levels of submucosal lymphocytes, thinner and more intact epithelium, thinner basal membranes, and fewer mucous glands were also observed in children. On the other hand, there are reports of eosinophilic inflammation in children with CRS refractory to appropriate medical treatment. Although the most likely correlation could be the association with allergy and asthma, the authors did not observe statistical significance. The prevalence of nasal polyps was lower when compared to adults, except in pediatric patients with cystic fibrosis. In adolescents, although rare, there are reports of Allergic Fungal Rhinosinusitis (AFRS) and Aspirin-Exacerbated Respiratory Disease (AERD).^538^

### Diagnosis of CRS in children

#### Clinical diagnosis

The clinical diagnosis of CRS in children often overlaps with other common childhood diseases, such as viral infections of the upper respiratory tract, hypertrophy of the pharyngeal tonsils/adenoiditis and allergic rhinitis. The most important signs and symptoms include nasal obstruction/congestion, purulent rhinorrhea (anterior/posterior), facial pain/pressure, and cough.

Other symptoms of CRS include halitosis and inappetence. Unlike adults, in whom hyposmia/anosmia is one of the cardinal symptoms for diagnosis, in children, nocturnal cough is often prominent and may be the most critical symptom reported by the parents.

#### Physical examination

Physical examination in children should include inspection of the nasal cavity and oropharynx, as well as palpation of the paranasal sinuses to check for tenderness. Nasal endoscopy (flexible or rigid) is an objective method that allows the visualization of purulent drainage, mucosal edema, presence of polyps, and anatomical anomalies that may eventually predispose to CRS.^3^, ^4^

#### Imaging exams

The diagnosis of CRS in children is not always easy. Although the combination of symptoms leads to high clinical suspicion, the diagnosis should be confirmed with imaging exams. As in adults, we reinforce the need for at least one imaging study to confirm the diagnosis. However, serial imaging studies should be avoided for treatment or disease monitoring purposes. It is important to remember that for diagnosis of CRS, imaging tests should not be performed during or up to 4-weeks after an acute exacerbation. The main imaging modalities include:

X-Ray of the paranasal sinuses

• It should not be used due to low sensitivity and specificity.

Computed Tomography (CT)

• Indications: Indicated for cases refractory to clinical treatment, suspected complications or anatomical anomalies.^4^
• Advantages: Provides detailed images of nasal anatomy and paranasal sinuses, allowing you to assess the extent of the disease as mucosal edema, bone remodeling, and to plan surgical interventions, when contemplated.^2^
• Limitations: Exposure to ionizing radiation, which should be minimized in children.

Magnetic Resonance Imaging (MRI)

• Indications: Rarely used in the diagnosis of CRS in children.
• Advantages: Does not use ionizing radiation and can help to differentiate between infection, tumors, and inflammation.
• Limitations: Lower availability, higher cost, and need for sedation (young children)

**The ABR recommends that imaging tests should be selective for diagnosis of CRS or complications in children. The routine use of imaging tests to monitor the disease or to assess the response to clinical treatment should be avoided.**

### Diferential diagnosis of CRS in children

Differential diagnosis is a crucial step in the management of CRS in children, as its symptoms may overlap with those of other clinical conditions including:

#### Allergic rhinitis

Symptoms of chronic allergic rhinitis, such as congestion and nasal discharge, can be mistaken with those of CRS. However, the presence of eye itching, sneezing, and seasonal symptoms can help distinguish them.^539^

#### Adenoiditis

Inflammation of the adenoids can cause symptoms like CRS, including nasal obstruction and cough, and hypertrophy of the pharyngeal tonsil is a common cause of chronic nasal congestion in children.^541^ The adenoid plays a significant role in the pathogenesis of CRS, and its surgical removal is usually beneficial for treatment.^540^, ^541^

#### Viral infections

Recurrent viral infections can also cause symptoms like CRS, particularly when the episodes overlap. The presence of acute inflammatory signs and symptoms such as fever and malaise help in the differential diagnosis.^4^

#### Anatomical anomalies

Abnormal structures of the nose or paranasal sinuses, such as a deviated septum or congenital malformations, may occasionally predispose to CRS and should be investigated.

**The ABR recommends that the diagnosis of CRS in children and its differential diagnostic conditions be carefully investigated with history, physical examination, and imaging exams.**

### Predisposing factors of CRS in children

The understanding of the predisposing factors of CRS in children is still unclear, and often controversial. Possible host- and environment-related factors include.

#### Anatomy of the nose and paranasal sinuses

Anatomical differences in children compared to adults, such as narrower ostia and drainage recesses, or anatomical variations such as bullous middle turbinate or septal deviation, do not seem to have the same impact as risk factors for CRS.^542^, ^543^

#### Adenoid hypertrophy

Several studies have shown that hypertrophy of the pharyngeal tonsil, a frequent condition in childhood, can be considered an important predisposing factor for CRS.^544^, ^545^ The involved pathophysiological mechanisms seem to be related not only to the obstructive process, but also to the local presence of biofilm.^546^ There is evidence of improvement of CRS in children after adenoidectomy.

#### Viral infections

Children have a high number of Upper Respiratory Tract Infections (URTIs). The mucosal edema, production of secretion and mucus retention, resulting from recurrent infections, could favor the development of CRS. However, studies do not show robust evidence that these viral infections would be predisposing factors for CRS in children.^547^, ^548^

#### Allergic rhinitis

Allergic Rhinitis (AR), commonly associated with nasal mucosal edema and reduced mucociliary clearance, is also a controversial predisposing factor for CRS in children. Studies show that the prevalence of sensitization to aeroallergens in children with CRS is high^549^, ^550^; however, the prevalence of CRS in atopic and non-atopic children is similar.

To date, there is no evidence of a clear relationship between AR and CRS in children.^551^

#### Asthma

Asthma is a clinical condition often associated with CRS. A study evaluating 4044 children with CRS identified that 18.1% of these children had associated asthma.^552^ Other studies have observed asthma exacerbation associated with CRS exacerbation. Despite the strong correlation between asthma and CRS, this association is not yet fully clarified.

#### Passive smoking

Active and passive exposure to cigarette smoke interferes with mucociliary clearance and epithelial regeneration. Thus, smoking, even if passive, has been considered a risk factor for CRS in children.

A systematic review evaluated and evidenced a strong correlation between exposure to cigarette smoke and the prevalence of CSR.^553^

Some studies have evaluated the outcomes of CRS surgery in the presence of passive and active smoking in patients under 18-years of age. Children exposed to cigarette smoke had a higher rate of surgical revision, worse SNOT scores, and lack of symptom improvement after surgery.^554^

Despite this evidence, more studies are necessary to determine whether smoking is in fact causal factor of CRS in children.

#### Gastroesophageal reflux disease

Gastroesophageal Reflux Disease (GERD) has been implicated as a causative factor of CRS in pediatric patients. The probable pathophysiological mechanism would be associated with inflammation in the ostia of the paranasal sinuses and alteration of mucociliary clearance. Some studies show that the diagnosis of CRS is more prevalent in children with GERD. Despite these findings, these studies have limitations such as a retrospective design and problems in the characterization of the sample.^555^, ^556^

#### Imunodeficiencies

Children with recurrent CRS or rhinosinusitis should be evaluated for possible primary immunodeficiencies. Studies show an increase in the prevalence of immunodeficiencies in children with CRS who do not respond to conventional treatment. Deficiencies of IgA, IgG and subclasses and poor serological switching to the pneumococcal vaccine were identified as possible predisposing factors.^537^, ^557^, ^558^

#### Cystic fibrosis

Cystic Fibrosis (CF) is an autosomal recessive disease caused by mutations in the CFTR gene leading to thickening of mucus, nasal polyps, and predisposing airway infections. The main pathogens found in the paranasal sinuses of these patients include *P. aureginosas* and S*. aureus*. Children with CRS and nasal polyps have a high prevalence of CF, reaching 100%. Thus, it is mandatory to investigate CF in all pediatric patients with CRS and nasal polyps.^559^, ^560^

#### Primary ciliar dyskinesia

Primary Ciliary Dyskinesia (PCD) is an autosomal recessive disease. Patients have defects in the mucociliary beat impacting the adequate mucociliary clearance. About 50% of patients with PCD have frequent rhinosinusitis and otitis, situs inversus totalis, and infertility. Symptoms of CRS are highly prevalent, with frequent purulent rhinorrhea and reduced lung function. Nasal polyps are less frequent than in CF. The diagnosis of PCD is often suspected by the presence of CRS associated with lung diseases such as bronchiectasis, as well as situs inversus totalis.^314^, ^561^

Several factors predisposing to or associated with CRS in children have been described, most of them without robust evidence (e.g., allergy, recurrent URI, smoking, GER, and asthma).

**The ABR recommends careful anamnesis, aiming not only to rule out the possible involved factors, but also to detect diseases frequently associated with CRS and often undiagnosed (e.g., PCD, CF and immunodeficiencies).**

### Medical treatment of CRS in children

Childhood CRS is a complex multifactorial inflammatory process and not just a persistent bacterial infection. Drug treatment aims to control inflammation, which predisposes the child to nasal congestion and obstruction, reducing the incidence of infections.^562^

Despite the lack of robust scientific evidence, it is not uncommon in daily practice to include antibiotics, intranasal corticosteroids, and saline nasal washes (with or without additives) as part of the treatment of CRS in children.

#### Antibiotics

Existing studies show that treating CRS in children with antibiotics for both short periods (up to 4-weeks) and longer periods (12‒15-weeks), as well as therapies including local or intravenous antibiotics are not justifiable.^4^

A study using macrolide subdoses for a long period resulted in 63% of satisfactory results, the authors suggest that it was due to a possible anti-inflammatory effect of the drug.^563^ According to some researchers, culturing material from the paranasal sinuses in CRS could help in the selection of the antimicrobial therapy. Microorganisms are usually *S aureus* and Gram-negative anaerobic bacteria, but most studies are based on empirical treatment.^562^, ^563^

**The ABR does NOT recommend the treatment of CRS in children with antibiotics, either orally in short or long treatments, combined with nasal solutions, or intravenously.**

#### Oral corticosteroids

The use of corticosteroids with gradual dose reduction in combination with antibiotics (antibiotics alone are not justifiable) appears to be more effective in CRS in children compared to placebo.

**The ABR does NOT recommend the use of oral corticosteroids in the treatment of CRS in children.**

#### Intranasal corticosteroids

Regarding intranasal corticosteroids, although there are no studies showing high levels of scientific evidence, it is believed that it can be used as part of the medical treatment in view of its potential anti-inflammatory effect and safety profile.^4^

**Therefore, the ABR recommends the use of intranasal corticosteroids for the treatment of CRS in children.**

#### Nasal saline solution

As with intranasal corticosteroids, studies on the use of nasal saline in CRS in children are scarce, and the level of evidence is not high. However, based on the benefit that it has been demonstrated in the adult population, it is usually recommended.

**The ABR recommends nasal saline solutions in the treatment of CRS in children.**

#### Immunomodulators

There is at least one study in children with CRS demonstrating that bacterial lysate can reduce the frequency and minimize the intensity of acute episodes, demonstrating a prophylactic effect for acute exacerbations of CRS in the pediatric population.^564^

**The ABR considers the use of immunomodulators in the treatment of CRS in children to be optional.**

### Surgical treatment of CRS in children

Surgical treatment is an alternative when there is no positive response after exhaustive drug therapy, that usually includes nasal washing with saline solution, intranasal corticosteroids spray; careful evaluation and treatment of possible comorbidities and, when necessary oral antibiotics in acute exacerbations. There are several surgical treatment modalities.

#### Adenoidectomy

Recommended as first-line surgical treatment, it has shown benefit mainly in children up to 6-years old, but it is believed that it can be effective even in older children, up to 12-years old.^565^ The pharyngeal tonsil is attributed the role of a reservoir of bacterial biofilm, also interfering with nasal physiology in patients with symptoms of nasal obstruction.^541^, ^566^

A recent study (2024) reports a high success rate in patients between 6- and 12-years of age, with 68% of patients experiencing remission of CRS symptoms after surgery.^567^ A meta-analysis of 9 studies of children aged 4- to 7-years showed improvement in CRS in 70% of cases.^546^ In general, the revision rate for adenoidectomy is very low (1.9%).^546^, ^568^ On the other hand, a 1999 nonrandomized study showed that after 6-months of follow-up, adenoidectomy alone had a success rate of 47%, while adenoidectomy associated with endoscopic endonasal sinus surgery had a success rate of 77%.^569^

The differential diagnosis between CRS and chronic adenoiditis prior to surgery should depend on objective measures such as nasal endoscopy or CT, since there is a significant overlap of the symptoms of these two clinical conditions.

There is a discrepancy between subjective measures and objective endoscopic findings in children with adenoiditis or CRS. Physical examination results may not reflect the impact of CRS on children’s quality of life.^570^ Among children with highly consistent symptoms of CRS, a Lund-Mackay score of 5 or higher can be considered a “positive” diagnosis for CRS with a high positive predictive value, sensitivity of 86% and specificity of 85%, whereas a CT score of less than 2 would present a high negative predictive value for the absence of chronic rhinosinusitis, probably indicating only the presence of isolated adenoiditis.^571^ Another retrospective study showed that in pediatric patients with Lund-Mackay scores greater than 5, the addition of maxillary sinus lavage at the time of adenoidectomy improved clinical symptoms up to one year after the procedure. Adenoidectomy alone was effective in 65% of patients with a Lund-Mackay score lower than 5, and 43% in those with a score greater than 5.^572^

**The ABR recommends adenoidectomy with no associated sinusectomy in children without underlying diseases with a surgical indication for the treatment of CRS.**

#### Endoscopic Sinus Surgery (ESS)

In two systematic reviews from 2013, the success rate was 82% in one, and 71%‒100% in the other, both with a low complication rate: 1.4% and 0.6%, respectively. However, most of these data were retrospective.^573^, ^574^ Ramadan *et al* observed that the use of corticosteroids at the beginning of the surgical treatment may reduce the need for a revision surgery.^575^ Younis, in a review of the available data, concluded that revisional surgery would not be necessary in most children after endoscopic sinus surgery.^576^

Initial concerns about the possible adverse effects of ESS on facial growth were alleviated by a long-term followup study by Bothwell *et al*^577^ that showed no impact of ESS on qualitative and quantitative parameters of pediatric facial growth. The follow-up was conducted for 10-years after surgery.^576^, ^577^ Regarding balloon sinuplasty, there are no prospective studies demonstrating its efficacy.^3^

Despite the surgical recommendations in pediatric CRS, it is important to remember that most children have spontaneous remission over the years.

**The ABR recommends that surgical interventions, when indicated, should be directed to the simplest procedures such as adenoidectomy and occasionally sinus irrigation, which have good success rates. The ABR also recommends that ESS be preferably indicated in children with comorbidities such as cystic fibrosis, primary ciliary dyskinesia, and immunodeficiencies. Surgical technique and extension of the procedure should be consistent and appropriate to the underlying disease.**

## Fungal rhinosinusitis

Fungi can be found in the nasal mucus of almost all healthy and diseased sinuses. However, there are several forms of sinus disease associated with fungi as pathogens. In these situations, rather than the fungi determining the disease process, it is usually the immune status of the host that determines the clinical presentation.^4^ Fungal Rhinosinusitis (FRS) can be divided into invasive or noninvasive rhinosinusitis, depending on the penetration of the fungus into the sinonasal mucosa. Invasive FRS is divided into acute and chronic, according to the speed of symptom onset; and non-invasive FRS can be classified into Fungal Ball (FB) and Allergic Fungal Rhinosinusitis (AFRS).^4^, ^578^

### Fungal ball

The fungal ball is a dense mass composed of fungal hyphae that accumulate inside the paranasal sinus. It occurs mainly in immunocompetent individuals and is distinguished from other fungal rhinosinusitis by not invading adjacent tissues. Usually, the development of this fungal mass is due to the inability of the sinonasal mucosa to eliminate inhaled fungi, which end up proliferating inside the paranasal sinus.^579^, ^580^, ^581^

The prevalence of fungal balls in the paranasal sinuses has increased in recent decades. This condition primarily affects middle-aged and older women, and studies report a female predominance of approximately 1.9:1.^582^ The incidence of fungal balls in patients who underwent endoscopic sinus surgery was reported to be around 5.4%.^580^, ^583^ The most frequently affected sinuses are the maxillary, followed by the sphenoid sinus.

Several factors can predispose the development of a fungal ball inside the sinus cavities. One of the main risk factors is a previous endodontic treatment, especially in the upper teeth, due to the possible penetration of dental materials into the maxillary sinus, creating a friendly environment to fungal growth.^580^ The aging of the population has also contributed, since the clearance and mucociliary decreases with the aging process, resulting in inadequate drainage, increasing the risk of fungal ball development.^582^, ^584^ Anatomical anomalies, such as nasal septum deviation and presence of a pneumatized middle turbinate, have also been associated with fungal ball formation.^585^, ^586^ Although most cases of fungal balls occur in immunocompetent individuals, conditions that involve immunosuppression, such as diabetes mellitus and immunosuppressants, may increase the susceptibility to its development.^587^, ^588^ Other related factors are bacterial or viral superinfections that can exacerbate the inflammatory process, providing additional nutrients for fungal growth.^589^, ^590^ Even after treatment of the fungal ball, factors such as chronic inflammation and mucosal remodeling can lead to persistence of symptoms.^591^

The diagnosis of a fungal ball is made through imaging studies and histopathological evaluation. Computed Tomography (CT) is essential, often detecting hyperdense areas within the affected sinus due to the accumulation of heavy metals in the fungal hyphae. Another common finding on CT scans is microcalcifications within the lesion.^581^, ^584^ Magnetic Resonance Imaging (MRI) complements CT, showing hypointense signal intensity on T1- and T2-weighted sequences, with contrast-enhancement of the mucosa.^585^ The identification of fungal hyphae through histopathological evaluation confirms the diagnosis.^585^, ^586^

Microbiological analyses, although useful, have low sensitivity, given the difficulty of growing fungi in culture.^587^, ^588^

The combination of these methods can accurately differentiate the fungal ball from other forms of rhinosinusitis, particularly the ones of invasive nature.^579^

The gold standard treatment is endoscopic surgery which predominantly consists of complete removal of the fungal debris from the paranasal sinuses.^581^, ^584^ Studies have shown that performing maxillary antrostomy is highly effective, ensuring adequate ventilation and drainage of the affected sinus and preventing from mucus accumulation with subsequent fungal growth.^584^ In some cases, an extended endoscopic approach may be necessary to access hard-to-reach areas and ensure complete removal of fungal debris.^585^ Recurrence may occur, particularly if the normal sinus function is not reestablished, requiring postoperative monitoring and ongoing management. Combining sinonasal saline irrigation with intranasal corticosteroids can reduce postoperative inflammation and promote faster healing.^585^, ^587^

**The ABR recommends surgical treatment for fungal balls.**

### Allergic fungal rhinosinusitis

Allergic Fungal Rhinosinusitis (AFRS) is a subtype of chronic rhinosinusitis that presents a type 2 immune response, characterized by chronic inflammation of the sinonasal mucosa associated with type I hypersensitivity to fungi.^4^ Compared to other types of Chronic Rhinosinusitis with Nasal Polyps (CRSwNP), AFRS is distinguished by the presence of fungi in the nasal secretion, thick eosinophilic mucin, usually darkened and with the appearance of peanut butter, with no signs of tissue invasion, unilateral or bilateral paranasal sinus opacification and remodeling with heterogeneity in secretion density observed on CT, and type I hypersensitivity to fungi.^592^

AFRS is a non-invasive form of fungal rhinosinusitis that usually affects younger adults, often under 30-years of age, and is more prevalent among women. In addition, it is more common in groups of lower socioeconomic power and individuals who live in warmer and more humid climates.^593^ Atopy is a predominant feature in patients with AFSR, and the presence of concomitant allergic diseases, such as allergic rhinitis and childhood-onset asthma, is very frequent. Although the association with asthma is common, it is less frequent than with other forms of CRSwNP.^594^ AFRS accounts for 5%–10% of CRS cases.^4^

A peculiarity of of AFRS in comparison to other types 2 CRS, is the association with an environmental factor, as the presence of fungi, which are known to induce a type 2 immune response. Exposure to environments with fungal spores leads to germination and immunogenic invasion, causing epithelial barrier dysfunction and release of cytokines IL-25, IL-33, and TSLP. This results in compensatory overstimulation of the type 2 immune response, perpetuating the inflammation and formation of nasal polyps.^593^, ^594^

Study by Den Beste *et al* revealed that the transepithelial resistance of cells in AFRS patients is lower compared to cells from healthy controls, indicating an altered mucosal barrier.^595^ In addition, exposure to type 2 cytokines such as IL-4 and IL-13 may further reduce this resistance.^596^

Patients with AFRS usually present with nasal congestion, olfactory dysfunction, thick darkened discharge, characteristic of eosinophilic mucin, and nasal polyps that can be visualized on nasal endoscopy or anterior rhinoscopy. This mucin is the product of an allergic hypersensitivity reaction to fungi, distinct from a simple overgrowth of mycelial elements, as seen in fungal balls. In severe cases, visual changes or even facial deformities may occur due to the progression of the disease. In general, relatively mild symptoms contrast with significant radiological findings, which may delay the diagnosis allowing further progression of the disease.

CT is the radiological exam of choice in the investigation of AFRS and shows opacified and expanded paranasal sinuses with heterogeneous densities.

Images may reveal a “dual-density signal”, which consists of highly attenuated sinus contents in contrast to the hypodense inflamed mucosa. In advanced cases, CT may show bone erosion and demineralization of the sinus walls.

Magnetic Resonance Imaging (MRI) shows mucosal edema with hyposignal on T2-weighted sequences, due to the presence of protein-rich eosinophilic mucin with low water content.

Laboratory findings of RSFA is characterized by elevated levels of total IgE (>500 IU/mL) and elevated levels of fungal-specific IgE. Peripheral eosinophilia may be present, although it is less common. Histopathological analysis confirms the absence of tissue invasion by the fungus, in addition to inflammatory cells on a background of amorphous eosinophilic mucin and necrotic cellular debris. Charcot-Leyden crystals, derived from the degranulation of eosinophils, can also be observed.^4^, ^593^, ^597^

Major Bent-Kuhn diagnostic criteria for AFRS can be seen in Table 21. The clinical criteria for rhinosinusitis (nasal polyps, eosinophilic mucin, and characteristic CT/MRI) are commonly found in AFRS. On the other hand, confirmation exams for the presence of the fungus and allergy to the fungus can often come as false negatives.^578^, ^592^

**Table 21:** AFRS diagnostic criteria.

1. Type I hypersensitivity (IgE-mediated)
2. Nasal polyps
3. Characteristic CT or MRI
4. Eosinophilic mucin without tissue invasion
5. Direct examination or histological positive staining for fungi

To increase diagnostic positivity, the minor Bent-Kuhn criteria may be considered: asthma, unilateral disease, serum eosinophilia, positive culture for fungi, and bone erosion.^592^

Currently, the recommended treatment for the management of AFRS is ESS, associated with intranasal and, occasionally, oral corticosteroids. The main goal of surgery is to remove eosinophilic mucus and nasal polyps, which are primarily responsible for perpetuating chronic inflammation in the sinuses. The surgery also allows the opening of the sinus cavities, facilitating the washing with saline solution and corticosteroid. However, the rate of revision surgery for patients with AFRS is high, requiring repeat surgery in about 28.7% of cases, compared to 18.6% in patients with chronic rhinosinusitis with nasal polyps (CRSwNP)^446^ (Table 21).

Corticosteroids play a key role in the postoperative management of AFRS. Historically, oral corticosteroids have been widely used and, although effective, the significant risks associated with their chronic or recurrent use, discourage their routine prescription, except in severe and refractory cases. Recently, the popularization of high volume saline nasal washing and the possibility to dilute corticosteroids in it, has made the administration of this medication easier and more effective. Therefore, optimal management of patients with AFRS includes endoscopic sinus surgery, followed by high-volume saline plus corticosteroid nasal lavage. Courses of oral corticosteroids should be reserved only for severe and exacerbating cases.

There aren’t many studies about other therapeutic classes, but they may represent new alternatives in the future. Intranasal and oral antifungals are discouraged, since AFRS is not an infection. And indeed, controlled clinical trials indicate that both oral and intranasal antifungals offer no significant benefit in the postoperative management when compared to corticosteroids.

However, recent research on topical Antimicrobial Peptides (AMPs) has shown a significant reduction of the nasal bacterial load and inflammatory markers, suggesting a possible new alternative to be explored.^598^ As for allergen immunotherapy, there are no randomized controlled trials evaluating its efficacy in AFRS, although some observational studies indicate a short-term benefit on symptoms.^599^, ^600^ Finally, therapy with biologics such as dupilumab, omalizumab, and mepolizumab has been established in the treatment of severe and refractory CRSwNP. These agents inhibit inflammatory mediators of type 2 immune response and could theoretically be effective in controlling AFRS as well. However, to date, no pivotal studies on biologics for the treatment of CRSwNP included patients with AFRS.^597^ Currently, some ongoing studies are evaluating the effect of dupilumab in this specific population (LIBERTY–AFRS-AI study, clinicaltrials.gov, NCT04684524).

**The ABR recommends surgery as treatment for patients with RSFA, followed by postoperative medical management.**

### Acute invasive fungal rhinosinusitis

Acute Invasive Fungal Rhinosinusitis (AIFRS) is defined as an invasion of fungal hyphae to the sinonasal epithelium, bone, and vessels resulting in thrombosis, tissue infarction, and necrosis.^4^, ^601^ The most frequently fungal species related to AIFRS are Aspergillus (69%), followed by Zygomycetes (Rhizopus, Mucor, Rhizomucor) (22%), Cryptococcus (4%) and Fusarium (2%).^578^, ^601^ AIFRS usually affects immunocompromised individuals, and the underlying disease and the degree of immunosuppression are more important than the type of fungus alone.^602^ The main risk patients group is the one with hematological malignant diseases, particularly those who have undergone bone marrow transplantation and those with severe neutropenia (absolute neutrophil count <500 mm^3^ and/or functional neutropenia). Prolonged severe neutropenia (<500 mm^3^ for more than 10-days) also increases the predisposition to invasive fungal disease in the nose and sinuses. Poorly controlled diabetes, chronic use of corticosteroids, acquired immunodeficiency and chemotherapy treatments are other risk groups of patients. Among these factors, diabetes (50%) and hematological malignancy (40%) account for 90% of the reported immunosuppression.^4^, ^578^, ^601^ More recently, COVID-19 has also become a major risk factor for the development of AIFRS.^603^, ^604^

The initial clinical history may be nonspecific.^601^, ^605^ The clinical presentation may include rhinorrhea, nasal obstruction, facial pain or pressure, and fever. Unilateral symptoms increase the diagnostic suspicion.^601^, ^605^ Facial edema is the most common clinical presentation (41%–65% of cases), but it usually occurs in more advanced cases.^601^ Fever is also highly prevalent (41%–67% of cases), and febrile neutropenia may be a warning sign for AIFRS in immunosuppressed patients.^601^, ^602^ Symptoms related to greater extent of the disease, in later cases, include ophthalmoplegia, proptosis, vision loss, ptosis, seizures, focal neurological deficits, or mental confusion.^4^, ^601^ Involvement of the III, IV, and VI cranial nerves may indicate cavernous sinus involvement.^605^

Physical examination findings depend on the stage of the disease at presentation. In the early stages, subtle changes in the nasal mucosa can be found, such as a pale or edematous nasal mucosa. As the disease progresses and neurovascular invasion worsens, the pale nasal mucosa becomes more avascular and turn out to be darker and necrotic. This mucosa will then ulcerate, forming a thick eschar or crust.^605^, ^606^ Necrosis of the nasal mucosa is the most consistent suggestive finding of AIFRS on physical examination. The middle turbinate is usually the most affected region.^578^, ^602^, ^606^ Similar findings can be seen in the nasal septum, sometimes causing septal perforation.^578^, ^602^, ^606^ Signs and symptoms related to the progression of invasive disease can appear quickly and worsen in few hours.^605^, ^606^

Unilateral disease in radiological findings is typical.^4^ Paranasal sinuses CT without contrast should always be performed as a screening. Initially, the most relevant characteristics include significant unilateral thickening of the nasal cavity mucosa (80%–91% of cases) and a predilection for unilateral periantral involvement (22%–38%). Other more specific, but later, findings are destruction of bone walls (bone erosion), orbital spread, and intracranial invasion.^578^, ^601^

If intracranial or orbital involvement is suspected, Magnetic Resonance Imaging (MRI) is the exam of choice. On MRI, the devitalized and necrotic mucosa of the AIFRS appears as a focal area with no diffusion of gadolinium contrast, known as LoCE (Lack of Contrast Enhancement).^601^ For diagnostic purposes, absence of contrast enhancement on magnetic resonance imaging (74 %–86 %) is more sensitive than on CT (69%).^4^, ^601^ Therefore, in cases of doubt regarding the extent of the disease, magnetic resonance imaging can be a tool to visualize necrotic areas suspected of angioinvasion and be useful for primary surgical planning or reinterventions.

When AIFRS is suspected, it is essential to perform nasal endoscopy and early biopsy of the affected site.^578^, ^606^ Direct fungal exam and fungal culture should be routinely requested. Considering the importance of early diagnosis for better prognosis, the biopsy must be referred for anatomopathological and frozen section evaluation.^578^, ^606^ The accuracy of the frozen section biopsy is high (83% sensitivity and 98% specificity).^607^ Histopathology of AIFRS shows submucosal hyphae-like formations with angiocentric invasion and tissue necrosis with few inflammatory cells. The biopsy should be adequate to provide material for analyzis, containing not only necrotic tissue. Culture does not have high sensitivity (51%–67%), but it is essential to direct the choice of antifungal therapy.^601^

**If AIFRS is suspected, biopsy should be performed as early as possible, and the material sent for anatomopathological and for frozen section biopsy examination.**

Once the diagnosis is confirmed, treatment should be carried out as early as possible. A multidisciplinary approach is extremely important, and the integration between clinical specialties (Infectious Diseases, Hematology, etc.) and surgical specialties (Otorhinolaryngology, Neurosurgery and Ophthalmology) is fundamental.^605^ Early diagnosis and treatment are essential to improve the survival of patients with AIFRS; it is known that longer time between diagnosis and treatment and greater extent of the lesion at diagnosis are important negative prognostic factors.^4^, ^601^, ^602^, ^605^^,^^606^ Treatment should be based on 3 basic principles:

1 Administration of antifungal
2 Surgical debridement of the necrotic tissue, and
3 Reversal or improvement of the individual’s immunosuppressive condition.^578^

Surgical debridement is very important in the treatment of AIFRS. All pale or necrotic tissue should be surgically removed until bleeding margins are obtained.^578^ Mortality rates range between 20% and 80%, and studies have shown increased survival when early diagnosis and surgical resection are accomplished.^601^, ^602^, ^608^ In many patients, multiple procedures may be necessary to control the extent of the disease. Endoscopic access is preferred in less extensive cases, but external access may be essential in cases of extrasinus involvement.

Early initiation of systemic antifungal therapy has shown to improve patient survival.^601^, ^608^ Liposomal amphotericin B should be administered empirically when invasive fungal rhinosinusitis is initially suspected. Once invasive aspergillosis is confirmed, the antifungal of choice is Voriconazole due to its effectiveness against Aspergillus. Intravenous therapy is generally recommended until effective clinical response and maximum reversal of the immunosuppression condition, which may last for weeks or months. Duration of treatment will depend on individual response, clinical and radiological improvement. Participation of the Infectious Diseases team is essential.^578^, ^601^

Overall survival for patients is higher: 1) In diabetic patients (survival rates are twice as high as those with severe neutropenia, due to the greater possibility of reversal and control of the predisposition to fungal invasion)^578^, ^601^; 2) When the interval between diagnosis and treatment is short (survival rate increases from 57% to 83% when the interval is less than 7-days between diagnosis and treatment);^601^, ^606^ 3) In patients with only sinonasal involvement (especially if unilateral, without involvement of the nasal septum).^601^, ^602^

**The ABR recommends that decompensated diabetic patients presenting sinonasal symptoms and those with febrile neutropenia should be screened for AIFRS by serial nasal endoscopy. Suspected cases should be diagnosed through biopsy (frozen section when available) and submitted to early surgical treatment.**

### Chronic invasive fungal rhinosinusitis

Fungal invasiveness of the sinonasal mucosa can also occur chronically and indolently. There are two distinct forms, Chronic Invasive Fungal Rhinosinusitis (CIFRS) and Granulomatous Invasive Fungal Rhinosinusitis (GIFRS).^609^ CIFRS is more common in diabetic or immunocompromised patients. The predominant symptoms are rhinorrhea (16%), nasal obstruction (18.5%), congestion, fever, facial pain (28.8%) and headache (31.9%). On the other hand, GIFRS manifests more often in immunocompetent patients, and the main symptom is proptosis (31.5% of cases) and orbital apex syndrome; no patient with GIFRS had fever according to the systematic review of Bahethi *et al*.^609^ In both diseases the findings on CT are mucosal thickening and sinonasal opacification, sclerosis or bone erosion, and mass effect with extrasinus extension. Extension to the orbit and pterygoid region are common, and in these cases, Magnetic Resonance Imaging (MRI) is indicated, showing a hypointense mass on T1- or LoCE sequences, or even septate hyperintensity on T2-weighted sequences, with absence of contrast enhancement.^609^

In both diseases, the main agent is Aspergillus (40%–60% of cases). As for AIFRS, treatment is based on endoscopic sinus surgery associated with systemic antifungal, either voriconazole (when *Aspergillus* is confirmed) or amphotericin. The extent of surgery depends on the extent of the disease, but in early to moderate cases, conservative endoscopic sinus surgery has been preferred to radical surgery and, has been indicated even in some cases with intracranial extension. In most cases, several procedures for surgical debridement are necessary, and clinical treatment can be as long as 6 to 9-months on average.^609^ Mortality rate is higher in patients with CIFRS (12%) than in those with GIFRS (2%), probably due to the patient’s underlying conditions.

## Conflicts of interest

The authors declare no conflicts of interest.
